# Supplementary material for: IrW nanochannel support enabling ultrastable electrocatalytic oxygen evolution at 2 A cm−2 in acidic media
Source: Nat Commun. 2021 Jun 10;12:3540. doi: 10.1038/s41467-021-23907-1 (PMC8192761; doi:10.1038/s41467-021-23907-1)
Supplement: Supplementary file 1 — Supplementary Information [file 41467_2021_23907_MOESM1_ESM.pdf]

Supplementary Information

IrW nanochannel support enabling ultrastable electrocatalytic oxygen evolution at 2 A·cm^{-2} in acidic media

Rui Li[1,4,6], Haiyun Wang[2,6], Fei Hu[3,6]*, K.C. Chan[4]*, Xiongjun Liu[5], Zhaoping Lu[5], Jing Wang[5], Zhibin Li[5], Longjiao Zeng[3], Yuanyuan Li[3], Xiaojun Wu[2]* & Yujie Xiong[2]*

[1]Northwestern Polytechnical University, Xi'an 710072, P. R. China

[2]Hefei National Laboratory for Physical Sciences at the Microscale iChEM (Collaborative Innovation Center of Chemistry for Energy Materials), and School of Chemistry and Materials Science, University of Science and Technology of China, Hefei 230026, P. R. China

[3]School of Materials Science and Energy Engineering, and Guangdong Key Laboratory for Hydrogen Energy Technologies, Foshan University, Foshan 528000, P. R. China

[4]Advanced Manufacturing Technology Research Centre, Department of Industrial and Systems Engineering, The Hong Kong Polytechnic University, Kowloon 999077, Hong Kong, China

[5]Beijing Advanced Innovation Center for Materials Genome Engineering, State Key Laboratory for Advanced Metals and Materials, University of Science and Technology Beijing, Beijing 100083, China

[6]These authors contributed equally: Rui Li, Haiyun Wang, and Fei Hu.;

*Corresponding author: E-mail: mfhufei@fosu.edu.cn (F.H.); kc.chan@polyu.edu.hk (K.C.); xjwu@ustc.edu.cn (X.W.); yjxiong@ustc.edu.cn (Y.X.)

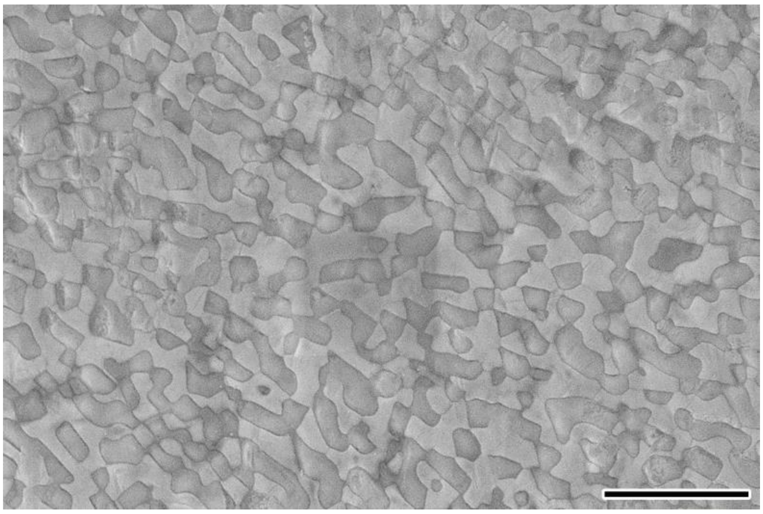

Supplementary Fig. 1 SEM image of the W-Ir-B alloy surface with an atomic ratio of 60:20:20. Scale bar: 1 μm.

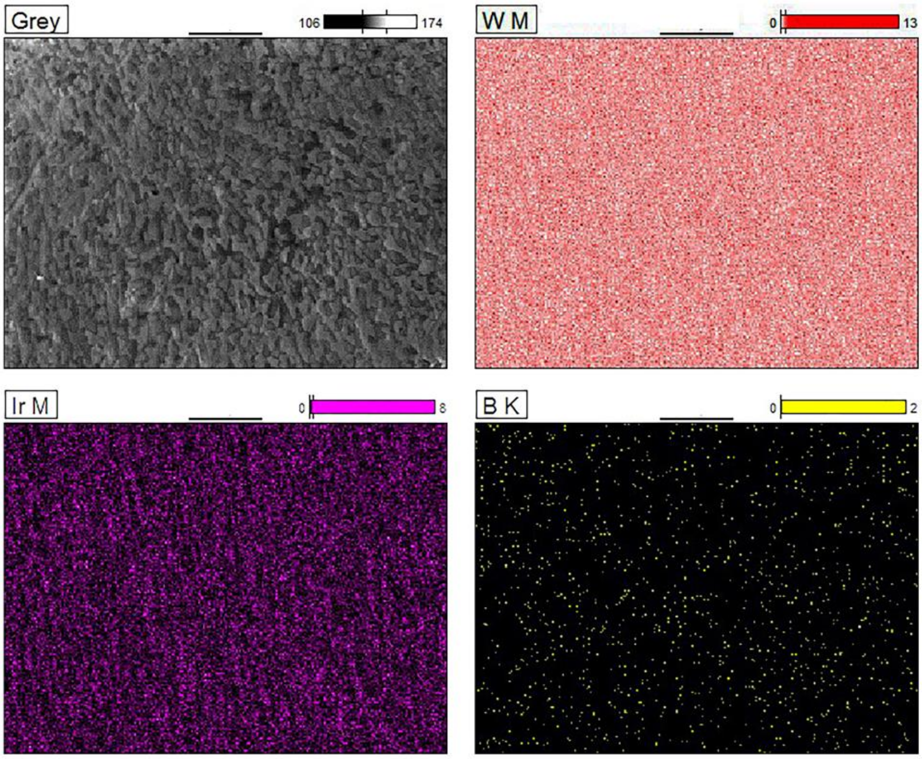

Supplementary Fig. 2 SEM images and EDS mapping profiles of the W-Ir-B alloy surface with an atomic ratio of 60:20:20. Scale bar: 1 μm.

a

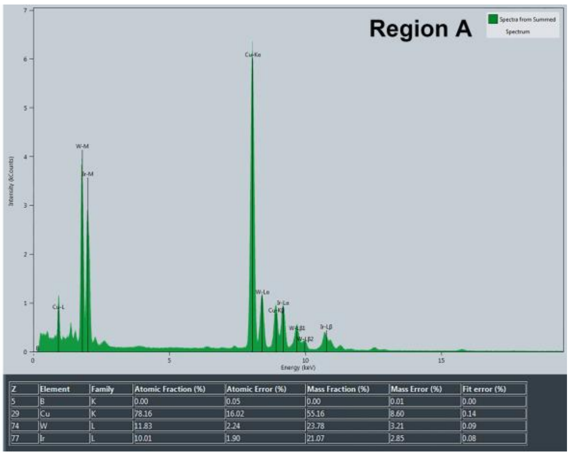

Element	***Line Type***	***k Factor***	***k Factor type***	***Absorption Correction***	***Wt%***	***Wt% Sigma***	***Atomic %***
B	K series	7.492		1.00	0.00	0.00	0.00
W	L series	2.186		1.00	50.32	0.59	51.43
Ir	L series	2.240		1.00	49.68	0.59	48.57
Total:					100.00		100.00

b

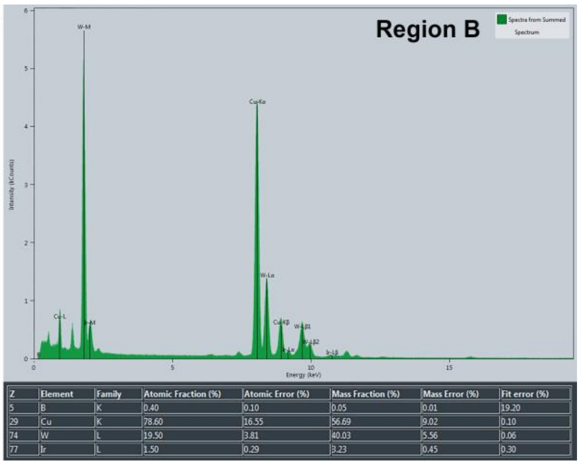

Element	***Line Type***	***k Factor***	***k Factor type***	***Absorption Correction***	***Wt%***	***Wt% Sigma***	***Atomic %***
B	K series	7.492		1.00	2.38	1.01	29.32
W	L series	2.186		1.00	94.72	1.17	68.67
Ir	L series	2.240		1.00	2.90	0.69	2.01
Total:					100.00		100.00

Supplementary Fig. 3 TEM EDS analysis of (**a**) region A and (**b**) region B in Fig. 1b.

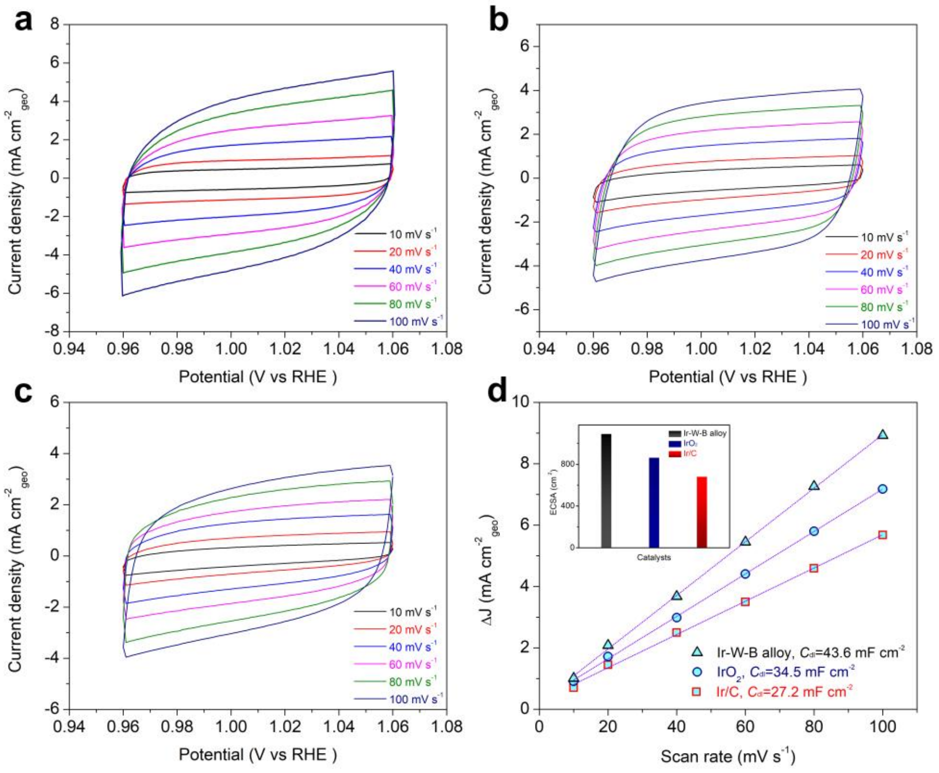

Supplementary Fig. 4 Cyclic voltammograms (CVs) of the (**a**) Ir-W-B alloy, (**b**) IrO_2, and (**c**) Ir/C catalysts, respectively. The CV curves were performed at different scan rates (10, 20, 40, 60, 80, and 100 mV s^{-1}) from 0.96 to 1.06 V vs RHE. (**d**) The plots of current densities against scan rates. Δj is the difference between anodic and cathodic current density at a potential of 1.01 V vs RHE. The slope of the fitting line is twice of the electrochemical double-layer capacitance, which is proportional to the ECSA of the catalysts. The ECSA value (inset in Fig. 4d) was calculated based on the equation of ECSA=$C_{\mathrm{dl}}/C_{\mathrm{s}}$, where C_{dl} is the electrochemical double-layer capacitance and C_s is the specific capacitance. The C_S for a flat surface was generally found to be in the range of 20 to 60 μF cm^{-2}. In this work, we assumed a value of 40 μF cm^{-2}.

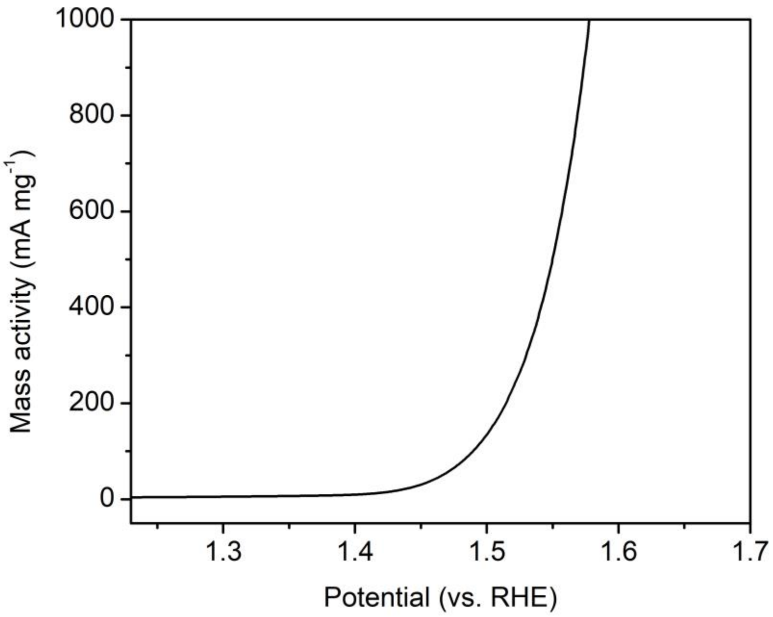

Supplementary Fig. 5 The Ir mass normalized current density vs. potential of the Ir-W-B alloy catalyst.

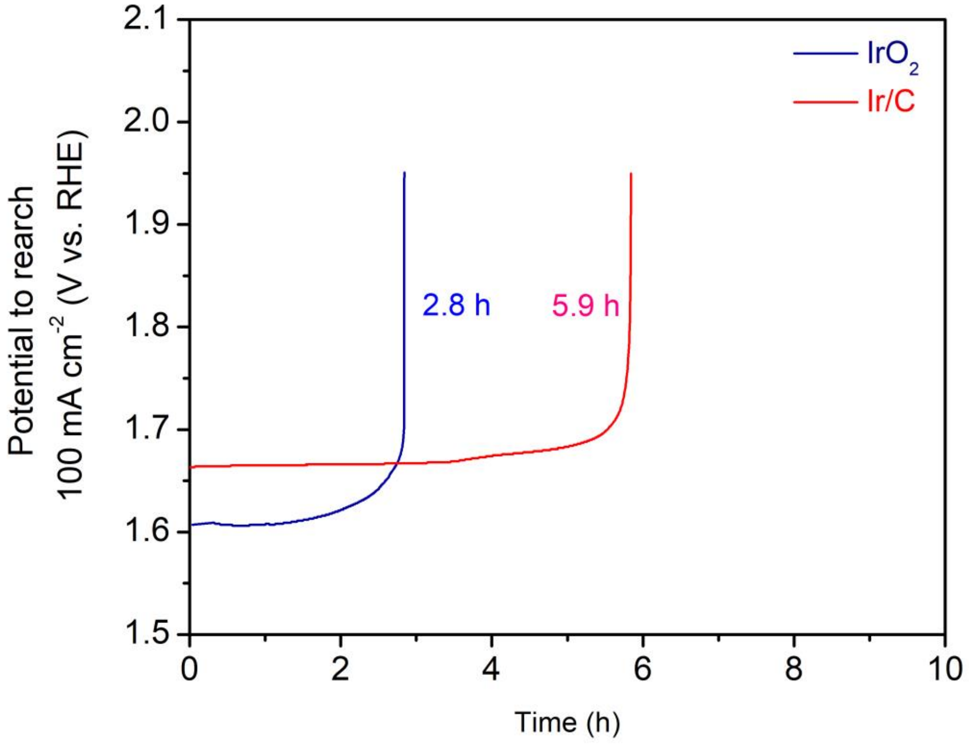

Supplementary Fig. 6 Chronopotentiometry curves of commercial IrO_2 and Ir/C catalysts in 0.5 M H_2SO_4 electrolyte at a current density of 100 mA cm^{-2}_{geo}. The efficacy loss is mainly due to the catalyst detachment resulting from the dissolution of the carbon support and the drastic attack of the continuous O2 bubbles.

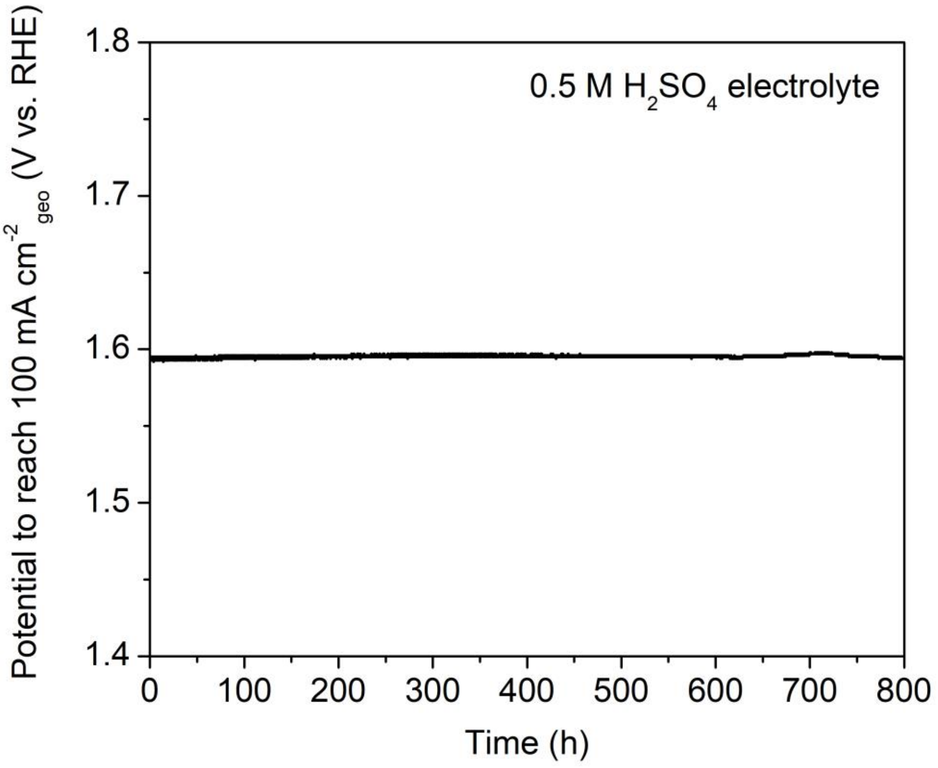

Supplementary Fig. 7 Durability test of the W-Ir-B alloy catalyst in 0.5 M H_2SO_4 electrolyte at a current density of 100 mA cm$^{-2}_{geo}$.

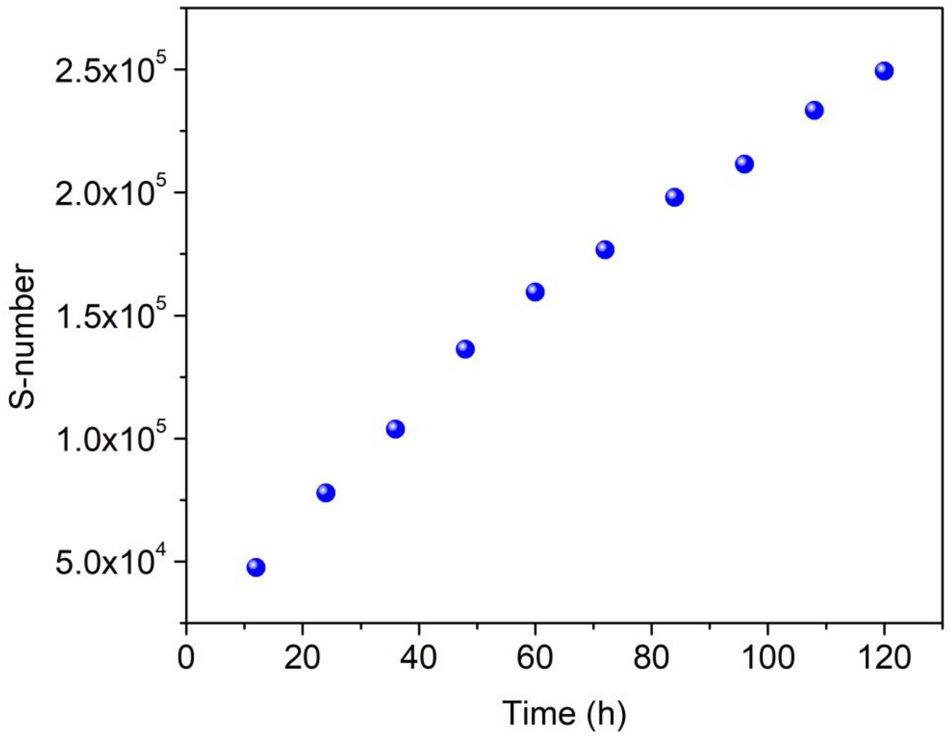

Supplementary Fig. 8 Calculated S-number during the 120-h acidic OER under the current density of 100 mA cm^{-2}_{geo}.

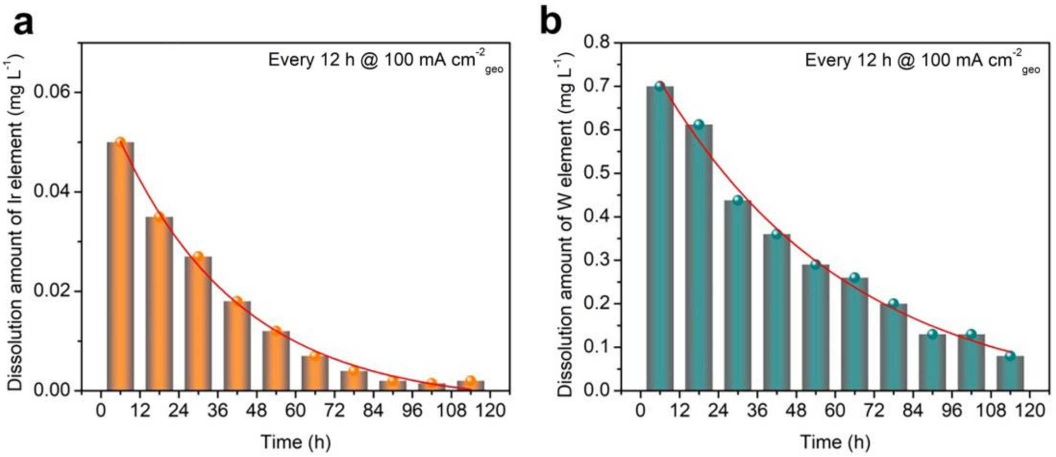

Supplementary Fig. 9 Dissolution amount variation of the (**a**) Ir and (**b**) W elements for every 12 h during the 120-h acidic OER test.

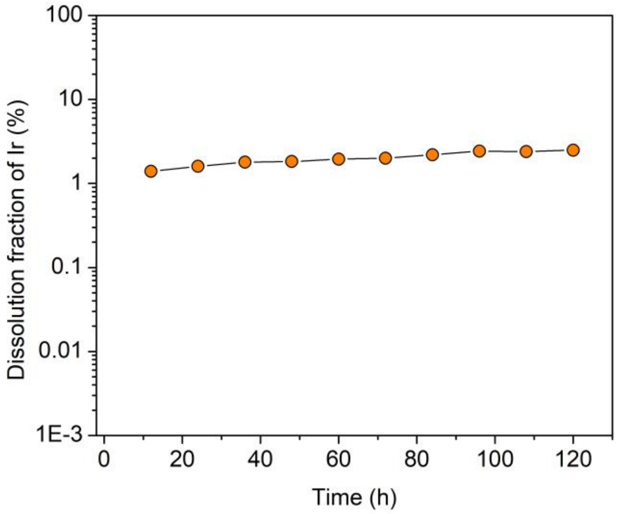

Supplementary Fig. 10 Dissolution of Ir element in comparison with the initial surface Ir mass during the 120-h acidic OER test at 100 mA cm^{-2}_{geo}.

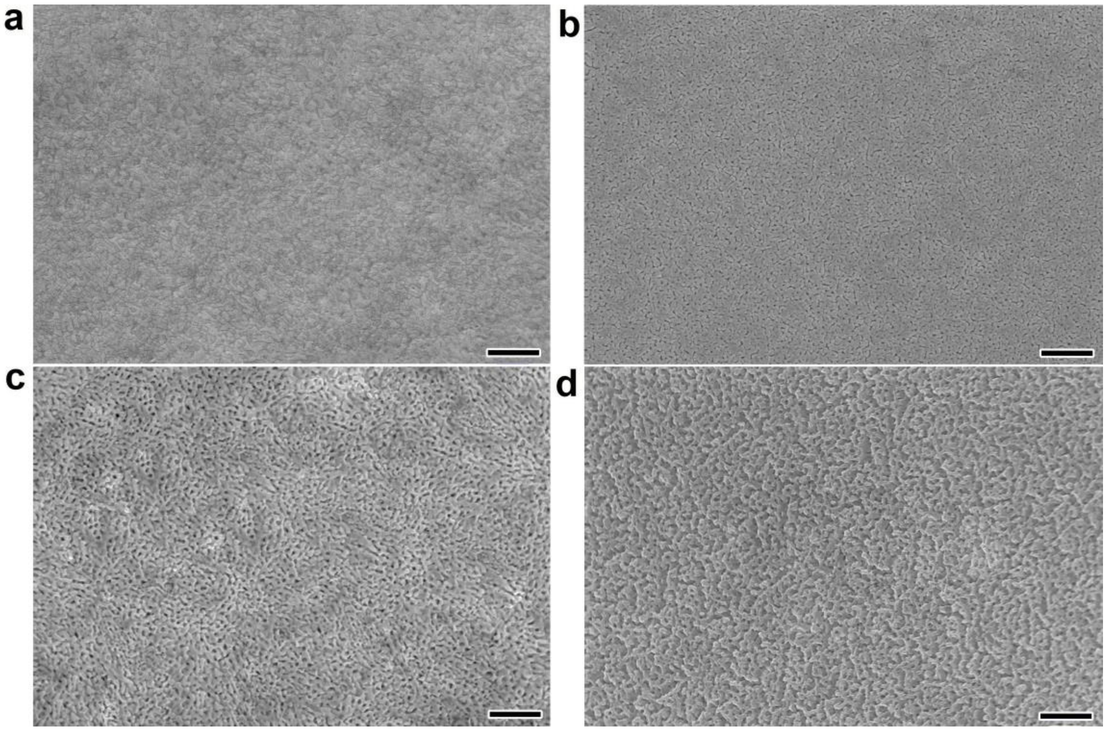

Supplementary Fig. 11 Surface structure evolution of the W-Ir-B alloy catalyst during the acidic OER: (**a**) 0 min; (**b**) 5 min; (**c**) 10 min; (**d**) 30 min. Scale bar: 1 μm.

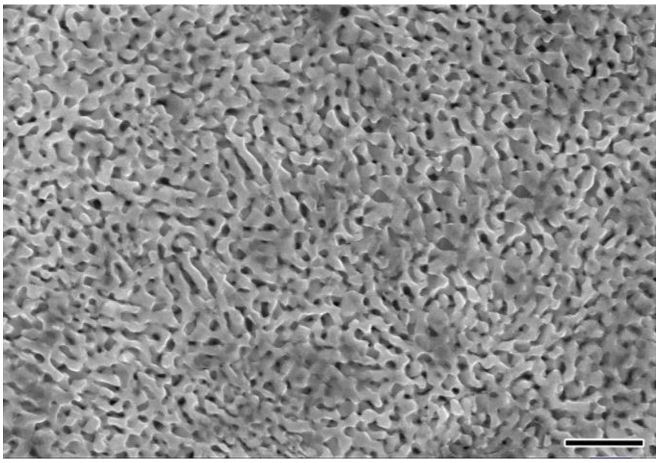

Supplementary Fig. 12 Top-view SEM images of the W-Ir-B alloy catalyst after the 2-h OER test. Scale bar: 500 nm.

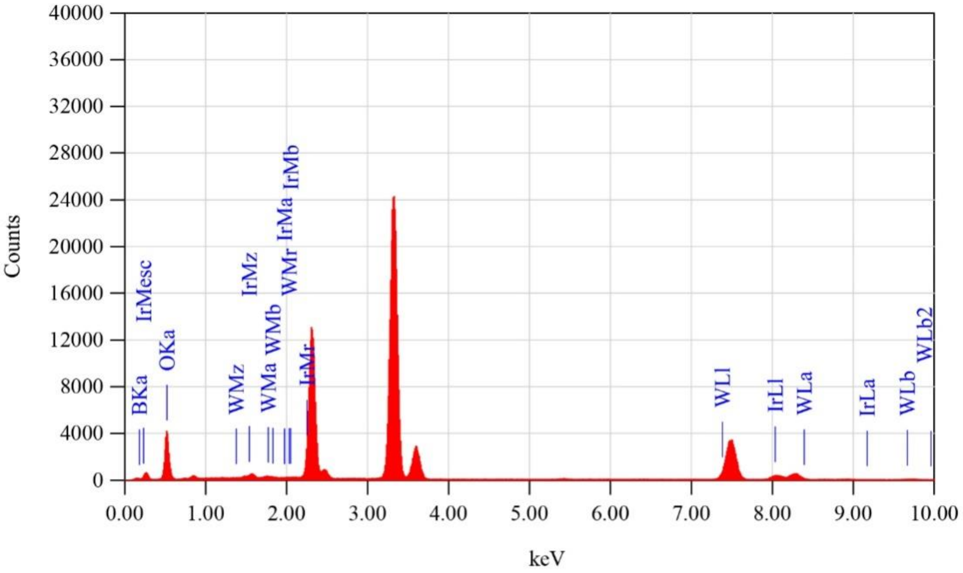

ZAF Method Standardless Quantitative Analysis
Fitting Coefficient : 0.9403

Element	(keV)	Mass%	Sigma	Atom%	Compound	Mass%	Cation	K
B K		ND		ND				
O K	0.525	35.68	0.23	86.90				52.4715
W M	1.774	6.19	0.83	1.31				5.5957
Ir M	1.977	58.12	2.02	11.78				41.9327
Total		100.00		100.00				

Supplementary Fig. 13 TEM EDS analysis for the surface active oxides.

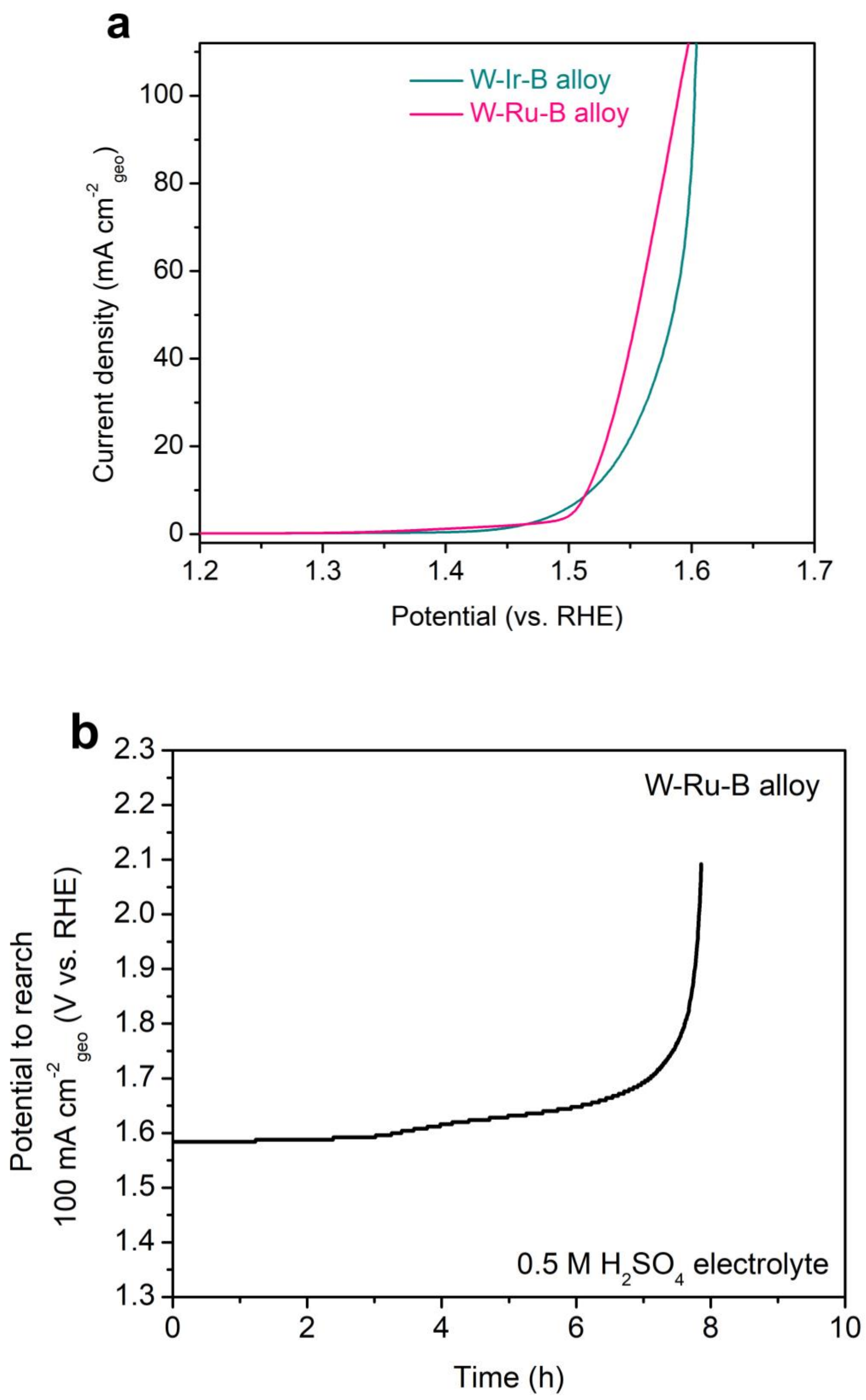

Supplementary Fig. 14 (**a**) OER polarization curves of the W-Ir-B and W-Ru-B alloy catalysts in 0.5 M H_2SO_4 electrolyte. (**b**) Chronopotentiometry curves of the W-Ru-B alloy catalyst at a current density of 100 mA cm^{-2}_{geo}.

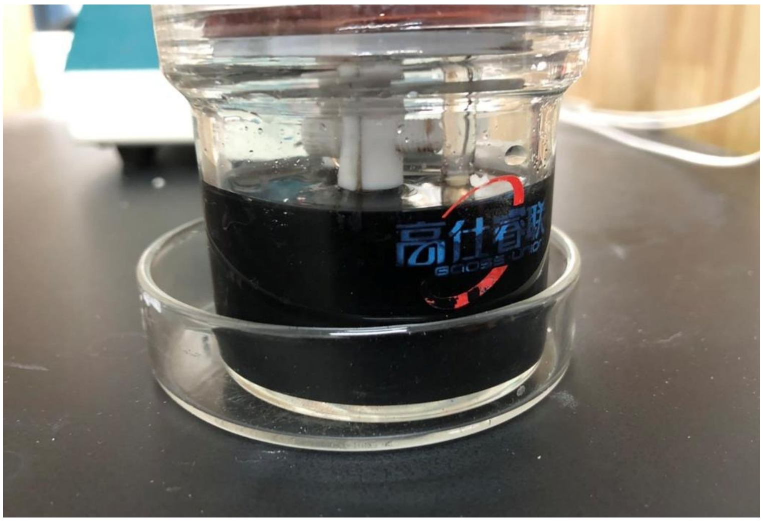

Supplementary Fig. 15 Electrolyte color of the W-Ru-B alloy catalyst after the 6-h OER durability test at the current density of 100 mA cm^{-2}_{geo}.

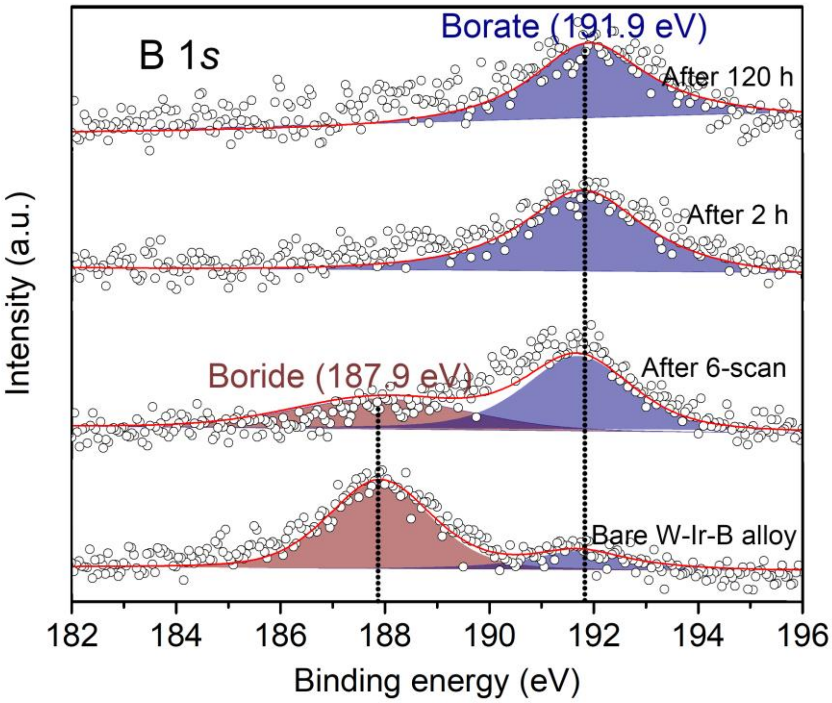

Supplementary Fig. 16 XPS analyses for B 1*s* of the bare W-Ir-B alloy catalyst, the OER activated alloy catalyst, the alloy catalyst after 2-h OER test, and the alloy catalyst after 120-h OER test.

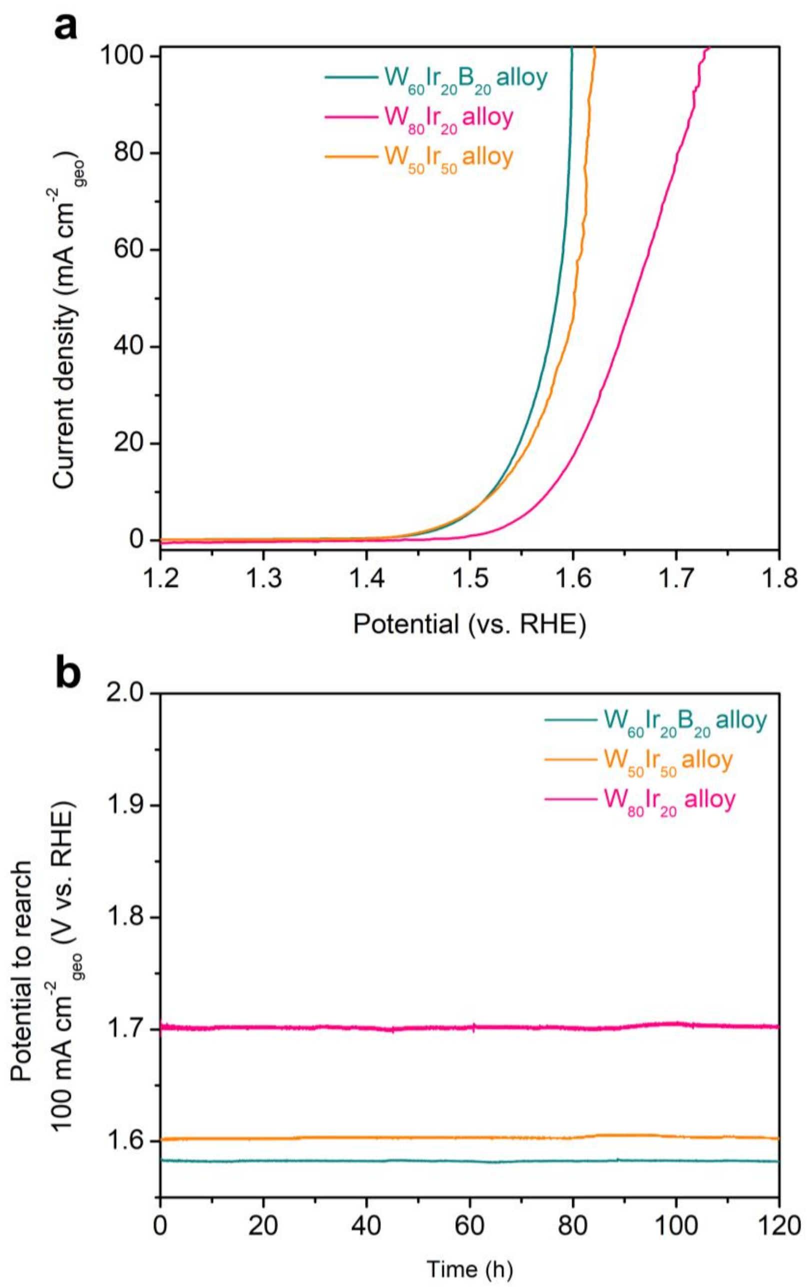

Supplementary Fig. 17 (**a**) OER polarization curves of the $W_{60}Ir_{20}B_{20}$, $W_{80}Ir_{20}$, and $W_{50}Ir_{50}$ alloy catalysts in 0.5 M H_2SO_4 electrolyte. (**b**) 120-h OER durability tests of the three alloy catalysts at a current density of 100 mA $cm^{-2}{}_{geo}$.

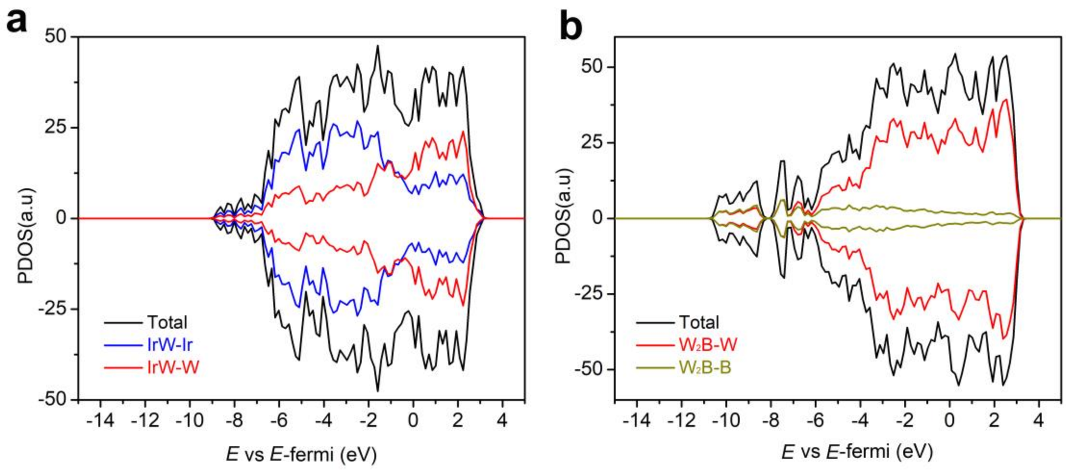

Supplementary Fig. 18 Computed partial density of states (PDOS) of (**a**) the IrW (002) surface and (**b**) the W_2B (211) surface.

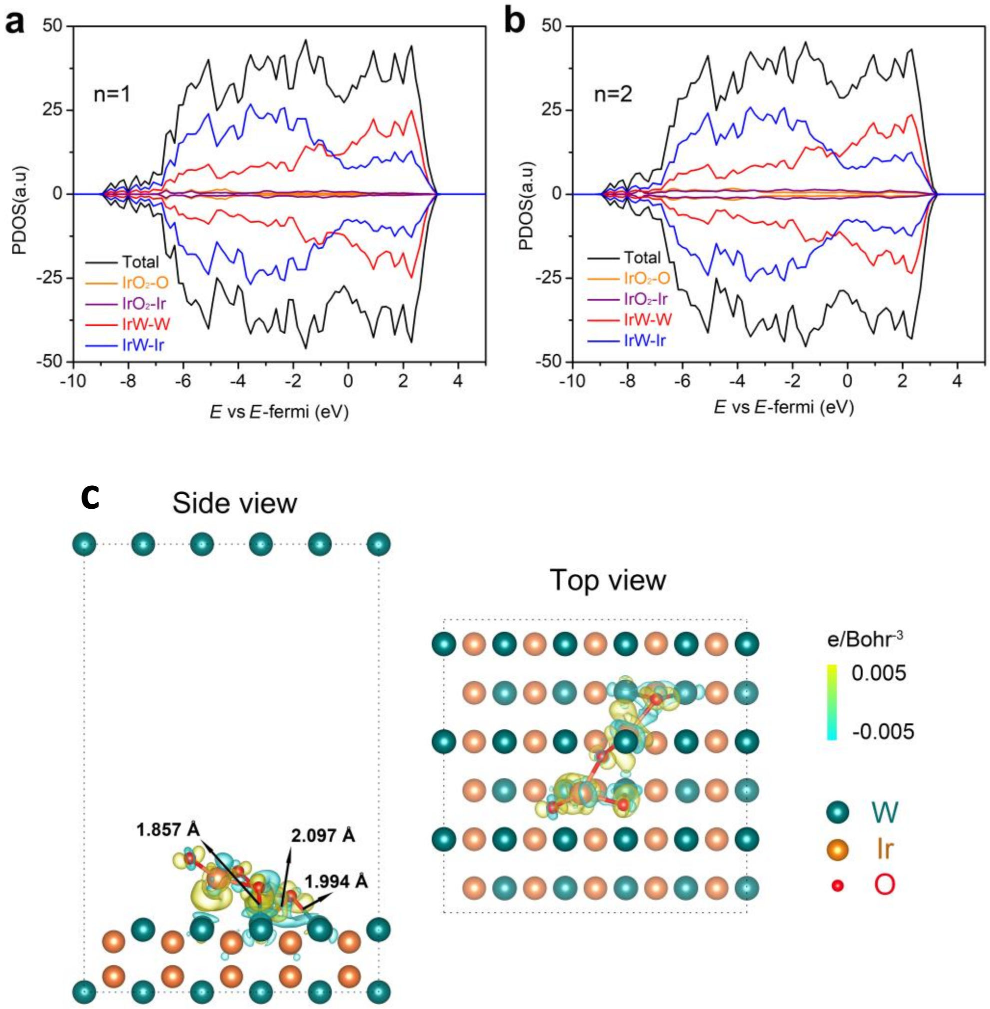

Supplementary Fig. 19 Computed PDOS of The $(IrO_2)_n$ clusters adsorbed on the IrW (002) surface: (**a**) n=1; (**b**) n=2. (**c**) Computed differential charge densities of the $(IrO_2)_2$ clusters adsorbed to the IrW (002) surface. Yellow and blue bubbles represent the positive and negative charge with an iso-value of 0.005 e Å^{-3}, respectively.

The calculation formula of the differential charge density is:

$$\Delta\rho = \rho_{AB} - \rho_A - \rho_B$$

ρ_A represents the charge density of the optimized structure after the IrO_2 adsorption on the IrW (002) surface, ρ_A and ρ_B represent the charge density of the IrW (002) surface and the IrO_2 clusters, respectively. By subtracting them, we can get the charge density change after the IrO_2 adsorption on the IrW (002) surface.

Supplementary Table 1 Comparison of overpotential and mass activity of the Ir-W-B alloy catalyst with previously reported Ir-based catalysts in acidic media.

Materials	Ir loading ($\mu g\ cm^{-2}$)	Overpotential@ 10 mA $cm^{-2}{}_{geo}$ (mV)	J_m@1.53 V vs. RHE (mA $mg_{Ir}{}^{-1}$)	Ref.
Ir-W-B alloy	78.9	291	518	This work
$IrNiO_x$	10.2	/	676	1
IrO_x	10.2	/	325	
La_2LiIrO_6	250 (catalyst)	/	<100	2
$SrIrO_3$	/	~290	/	3
Ba_2NdIrO_6	15 (catalyst)	380	/	4
IrO_x-network	10	~330	817±119	5
Li-IrO_x	50 (catalyst)	300	100	6
Pt-Ir-Pd nanocage	16.8	372	200	7
IrO_2 nanoneedles	250	313	~55	8
IrNiCu nanoframe	20	300	460	9
6H-$SrIrO_3$	52.7	248	75	10

Supplementary Table 2 Comparison of the OER performance and stability for the recently reported electrocatalysts in acidic media.

Materials	substrate	Electrolyte	Overpotential at 10 mA cm^{-2}_{geo} (mV)	Tafel slope (mV dec^{-1})	Test condition	Stability (h)	Ref.
$W_{60}Ir_{20}B_{20}$	Self-supported	0.5 M H_2SO_4	291	78	100 mA cm^{-2}_{geo}	>800	This work
$IrO_x/SrIrO_3$	$SrTiO_3$	0.5 M H_2SO_4	270-290	/	10 mA cm^{-2}_{geo}	30	3
Ir nanosheet	Glassy carbon	0.5 M H_2SO_4	240	49	10 mA cm^{-2}_{geo}	>8	11
Co_3O_4@C	Carbon paper	0.5 M H_2SO_4	370	82	100 mA cm^{-2}	86.8	12
IrOOH Nanosheet	Ti platelet	0.1 M $HClO_4$	344	58	10 mA cm^{-2}_{geo}	<6	13
Nanoporous $Ir_{70}Ni_{15}Co_{15}$	Self-supported	0.1 M $HClO_4$	220	44.1	10 mA cm^{-2}_{geo}	10	14
$Ir_{73.9}W_{26.1}$ ND	Glassy carbon	0.1 M $HClO_4$	300	56.6	10 mA cm^{-2}_{geo}	8	15
Ir/Co_4N	Glassy carbon	0.5 M H_2SO_4	310	62	10 mA cm^{-2}_{geo}	2	16
IrNi nanocluster	Glassy carbon	0.5 M H_2SO_4	280	/	10 mA cm^{-2}_{geo}	10	17
Ir_6Ag_9 nanotubes	Glassy carbon	0.5 M H_2SO_4	285	61.1	5 mA cm^{-2}_{geo}	36	18
Ir single atoms	Glassy carbon	0.5 M H_2SO_4	250	58.2	10 mA cm^{-2}_{geo}	12	19
Nanoporous AlNiCoIrMo	Self-supported	0. 5 M H_2SO_4	233	55.2	10 mA cm^{-2}_{geo}	50	20
NiIr Nanocage	Glassy carbon	0.05 M H_2SO_4	301	46.6	1 mA cm^{-2}_{geo}	10	21
Ir@IrO_x	Glassy carbon	0.05 M H_2SO_4	282	69.1	10 mA cm^{-2}_{geo}	24	22
IrO_2/CNT	Glassy carbon	0.5 M H_2SO_4	293	67	10 mA cm^{-2}_{geo}	10	23
IrO_x	carbon cloth	0.5 M H_2SO_4	185	35.5	50 mA cm^{-2}_{geo}	50	24
Ir/Graphite foam	Self-supported	0.5 M H_2SO_4	290	46	10 mA cm^{-2}_{geo}	10	25
$Ir_{0.5}$W-900R	Glassy carbon	0.1 M $HClO_4$	290~300	42	10 mA cm^{-2}_{geo}	100	26

Reference

1. Nong, H.N. *et al.* A unique oxygen ligand environment facilitates water oxidation in hole-doped $IrNiO_x$ core-shell electrocatalysts. *Nat. Catal.* **1**, 841-851 (2018).
2. Grimaud, A. *et al.* Activation of surface oxygen sites on an iridium-based model catalyst for the oxygen evolution reaction. *Nat. Energy* **2**, 1-10 (2016).
3. Seitz, L.C. *et al.* A highly active and stable $IrO_x/SrIrO_3$ catalyst for the oxygen evolution reaction. *Science* **353**, 1011-1014 (2016).
4. Diaz-Morales, O. *et al.* Iridium-based double perovskites for efficient water oxidation in acid media. *Nat. Commun.* **7**, 1-6 (2016).
5. Jensen, A.W. *et al.* Self-supported nanostructured iridium-based networks as highly active electrocatalysts for oxygen evolution in acidic media. *J.Mater. Chem. A* **8**, 1066-1071 (2020).
6. Gao, J. *et al.* Breaking long-range order in iridium oxide by alkali ion for efficient water oxidation. *J. Am. Chem. Soc.* **141**, 3014-3023 (2019).
7. Zhu, J. *et al.* Pt-Ir-Pd Trimetallic nanocages as a dual catalyst for efficient Ooxygen reduction and evolution reactions in acidic media. *Adv. Energy Mater.* **10**, 1904114 (2020).
8. Lim, J. *et al.* Ultrathin IrO_2 nanoneedles for electrochemical water oxidation. *Adv. Funct. Mater.* **28**, 1704796 (2018).
9. Park, J., Sa, Y.J., Baik, H., Kwon, T., Joo, S.H. & Lee, K. Iridium-based multimetallic nanoframe@nanoframe structure: An efficient and robust electrocatalyst toward oxygen evolution reaction. *ACS Nano* **11**, 5500-5509 (2017).
10. Yang, L. *et al.* Efficient oxygen evolution electrocatalysis in acid by a perovskite with face-sharing IrO_6 octahedral dimers. *Nat. Commun.* **9**, 1-9 (2018).
11. Jiang, B. *et al.* Mesoporous metallic iridium nanosheets. *J. Am. Chem. Soc.* **140**, 12434-12441 (2018).
12. Yang, X. *et al.* Highly acid-durable carbon coated Co_3O_4 nanoarrays as efficient oxygen evolution electrocatalysts. *Nano Energy* **25**, 42-50 (2016).
13. Weber, D. *et al.* IrOOH nanosheets as acid stable electrocatalysts for the oxygen evolution reaction. *J. Mater. Chem. A* **6**, 21558-21566 (2018).

14. Zhao, Y. *et al.* 3D nanoporous iridium-based alloy microwires for efficient oxygen evolution in acidic media. *Nano Energy* **59**, 146-153, (2019).
15. Lv, F. *et al.* Iridium-tungsten alloy nanodendrites as pH-universal water-splitting electrocatalysts. *ACS Central Sci.* **4**, 1244-1252 (2018).
16. Tackett, B.M. *et al.* Reducing iridium loading in oxygen evolution reaction electrocatalysts using core-shell particles with nitride cores. *ACS Catal.* **8**, 2615-2621 (2018).
17. Pi, Y., Shao, Q., Wang, P., Guo, J. & Huang, X. General formation of monodisperse IrM (M= Ni, Co, Fe) bimetallic nanoclusters as bifunctional electrocatalysts for acidic overall water splitting. *Adv. Funct. Mater.* **27**, 1700886 (2017).
18. Zhu, M., Shao, Q., Qian, Y. & Huang， X. Superior overall water splitting electrocatalysis in acidic conditions enabled by bimetallic Ir-Ag nanotubes. *Nano Energy* **56**, 330-337 (2019).
19. Luo, F. *et al.* Robust and stable acidic overall water splitting on Ir single atoms. *Nano Lett.* **20**, 2120-2128 (2020).
20. Jin, Z. *et al.* Nanoporous Al-Ni-Co-Ir-Mo high-entropy alloy for record-high water splitting activity in acidic environments. *Small* **15**, 1904180 (2019).
21. Wang, C., Sui, Y.M., Xu, M., Liu, C., Xiao, G.J. & Zou, B. Synthesis of Ni-Ir nanocages with improved electrocatalytic performance for the oxygen evolution reaction. *ACS Sustainable Chem. Eng.* **5**, 9787-9792 (2017).
22. Shan, J. *et al.* Charge-redistribution-enhanced nanocrystalline Ru@IrO_x electrocatalysts for oxygen evolution in acidic media. *Chem* **5**, 445-459 (2019).
23. Guan, J., Li, D., Si, R., Miao, S., Zhang, F. & Li, C. Synthesis and demonstration of subnanometric iridium oxide as highly efficient and robust water oxidation catalyst. *ACS Catal.* **7**, 5983-5986 (2017).
24. Jiang, H. *et al.* Self-supported iridium oxide nanostructures for electrocatalytic water oxidation in acidic media. *J. Phys. Chem. C* **124**, 2-8 (2019).
25. Zhang, J. *et al.* Iridium nanoparticles anchored on 3D graphite foam as a bifunctional electrocatalyst for excellent overall water splitting in acidic solution. *Nano Energy* **40**, 27-33 (2017).

26. Gao, J., Huang, X., Cai, W., Wang, Q., Jia, C. & Liu, B. Rational design of an iridium-tungsten composite with an iridium-rich surface for acidic water oxidation. *ACS Appl. Mater. Interfaces* **12**, 25991-26001 (2020).
