# Supplementary material for: IrW nanochannel support enabling ultrastable electrocatalytic oxygen evolution at 2 A cm−2 in acidic media
Source: Nat Commun. 2021 Jun 10;12:3540. doi: 10.1038/s41467-021-23907-1 (PMC8192761; doi:10.1038/s41467-021-23907-1)
Supplement: Supplementary file 2 — Source Data [file 41467_2021_23907_MOESM2_ESM.zip › Manuscript-clean.pdf]

IrW nanochannel support enabling ultrastable electrocatalytic oxygen evolution at 2 $A \cdot cm^{-2}$ in acidic media

Rui Li[1,4,6], Haiyun Wang[2,6], Fei Hu[3,6]*, K.C. Chan[4]*, Xiongjun Liu[5], Zhaoping Lu[5], Jing Wang[5], Zhibin Li[5], Longjiao Zeng[3], Yuanyuan Li[3], Xiaojun Wu[2]* & Yujie Xiong[2]*

[1]Northwestern Polytechnical University, Xi'an 710072, P. R. China

[2]Hefei National Laboratory for Physical Sciences at the Microscale iChEM (Collaborative Innovation Center of Chemistry for Energy Materials), and School of Chemistry and Materials Science, University of Science and Technology of China, Hefei 230026, P. R. China

[3]School of Materials Science and Energy Engineering, and Guangdong Key Laboratory for Hydrogen Energy Technologies, Foshan University, Foshan 528000, P. R. China

[4]Advanced Manufacturing Technology Research Centre, Department of Industrial and Systems Engineering, The Hong Kong Polytechnic University, Kowloon 999077, Hong Kong, China

[5]Beijing Advanced Innovation Center for Materials Genome Engineering, State Key Laboratory for Advanced Metals and Materials, University of Science and Technology Beijing, Beijing 100083, China

[6]These authors contributed equally: Rui Li, Haiyun Wang, and Fei Hu.

*Corresponding author: E-mail: mfhufei@fosu.edu.cn (F.H.); kc.chan@polyu.edu.hk (K.C.); xjwu@ustc.edu.cn (X.W.); yjxiong@ustc.edu.cn (Y.X.)

Abstract

A grand challenge for proton exchange membrane electrolyzers is the rational design of oxygen evolution reaction electrocatalysts to balance activity and stability. Here, we report a support-stabilized catalyst, the activated ~200 nm-depth IrW nanochannel that achieves the current density of 2 A cm^{-2} at an overpotential of only ~497 mV and maintains ultrastable gas evolution at 100 mA cm^{-2} at least 800 h with a negligible degradation rate of ~4 μV h^{-1}. Structure analyses combined with theoretical calculations indicate that the IrW support alters the charge distribution of surface $(IrO_2)_n$ clusters and effectively confines the cluster size within 4 (n⩽4). Such support-stabilizing effect prevents the surface Ir from agglomeration and retains a thin layer of electrocatalytically active IrO_2 clusters on surface, realizing a win-win strategy for ultrahigh OER activity and stability. This work would open up an opportunity for engineering suitable catalysts for robust proton exchange membrane-based electrolyzers.

Introduction

Proton exchange membrane (PEM) water electrolyzers have attracted growing attention owing to high current density, excellent voltage efficiency, and ultrahigh gas purity[1-3], which are perfectly 0~100% adaptive for transforming and storing renewable energy sources such as solar, wind, and hydro energy. Although green hydrogen is deemed as a vital part of next-generation energy sources[4, 5], the large-scale implementation and commercialization of PEM water electrolyzers in hydrogen production are severely limited by delicate catalysts[6, 7]. In particular, anodic oxygen evolution reaction (OER) electrocatalysts severely suffer from their low stability in acid media[8, 9]. To facilitate the practical industrial application of PEM electrolyzers, it is imperative to develop highly active OER electrocatalysts that can operate robustly in strongly acidic media. However, OER activity and stability are often in a dilemma in electrocatalyst design.

Up to now, iridium (Ir) has been recognized as the best OER catalytic material in acid media in terms of activity and stability[10]. For this reason, great efforts have been devoted to the design and synthesis of nanostructured Ir-based electrocatalysts for acidic OER, such as IrO_2/carbon nanotubes, IrAg nanotubes, IrRu nanoclusters, and IrW nanodendrites[11-16]. Yet the reported OER catalysts can hardly meet the strict criteria for industrial application–high current densities over 2 A $cm^{-2}{}_{geo}$ at low overpotentials (≤500 mV)[17, 18]. The most recently reported stable Ir-based nanostructured catalysts tend to lose efficiency after 120 h at the current density of 10 mA $cm^{-2}{}_{geo}$ under the acidic environment. As a matter of fact, the dissolution of Ir-based catalysts is unavoidable and even severe for nanosized particles as normalized by specific surface area. The choice of support is highly important for PEM electrolyzer from the perspective of engineering, but is long unfocused for catalyst design. In 2016, P. Strasser et al. have revealed a support-stabilizing effect for the contribution to the

corrosion stability of Ir catalyst in acidic water splitting, in which the electronic interaction between catalyst and support can effectively suppress the growth of higher-valent Ir oxides and the subsequent dissolution[19]. The catalyst-support interactions have been investigated on various substrates such as carbon, Ni foam, TiO_x, and antimony-doped tin oxide (ATO)[20-24]. However, carbon and Ni can hardly be used in practice as they would be oxidized at relatively low anodic potentials. Even for the ATO support materials, it may become unstable under harsh acidic OER conditions[25]. A right choice of support should be resistant to corrosion and electronically tunable. As such, we decide to focus on the high-valence metal W for investigating catalyst-support interactions that may balance the OER activity and stability in acidic media[15, 26].

In this work, to elucidate the support-stabilizing effect from bulk materials, we intentionally design a biphasic IrW-W_2B (W-Ir-B) alloy. By continuously undergoing selective corrosion on W_2B, an electrocatalytically active IrW nanochannel layer with ~200 nm depth is emerged as a highly efficient and ultrastable electrocatalyst for acidic OER. The supported catalyst yields a stable, high current density of 2 A cm^{-2}_{geo} with a low overpotential of ~497 mV, which can maintain at least 800 h at 100 mA cm^{-2}_{geo} with a negligible degradation rate of ~4 μV h^{-1}, surpassing the state-of-the-art specification (14 μV h^{-1}) in strongly acidic media (pH=0)[27]. As indicated by our density functional theory (DFT) calculations, the impressive catalyst-support interactions in the IrW nanochannels can alter the charge distribution of surface Ir and O atoms [i.e., $(IrO_2)_n$ clusters] and confine the cluster size of $(IrO_2)_n$. As a result, such an effect prevents the Ir atoms from accumulating more O atoms to form soluble high-valence state counterparts, as well as retain a thin layer of electrocatalytically active IrO_2 clusters on the substrate. This finding provides opportunities for designing an acidic OER electrocatalyst with high activity and long-term stability at industrial large current

densities for PEM-based electrolyzer.

Results

Material fabrication and characterizations. The biphasic alloy was facilely fabricated by a water-cooling copper mold casting method under a high-purity argon atmosphere. Fig. 1a shows the X-ray diffraction (XRD) pattern of the rapidly solidified W-Ir-B alloy rod (atomic ratio, 60:20:20) with a diameter of 1 mm, a characteristic of a two-phase alloy containing the crystalline phases of orthorhombic IrW and tetragonal W_2B. In agreement with the XRD characterization, scanning electron microscopy (SEM) image and energy-dispersive X-ray spectroscopy (EDS) mapping profiles also reveal the diphasic surface of the W-Ir-B alloy sample(Supplementary Figs. 1, 2). The crystallinity and elemental distribution of the W-Ir-B alloy were further characterized by transmission electron microscopy (TEM) and scanning transmission electron microscopy (STEM) element mapping. TEM image (Fig. 1b) shows that the separated crystalline phases present a bi-continuous and inter-connected structure in the alloy, mainly resulting from the high cooling rate during melting. As revealed by EDS analysis (Supplementary Fig. 3), region A and region B corresponds to the IrW phase and the W-rich phase, respectively. The corresponding selected area electron diffraction (SAED, Fig. 1c) further confirms that the two regions are orthorhombic IrW and tetragonal W_2B, respectively. In fact, the two crystalline phases can be identified by the contrast difference in their high-angle annular diffraction field (HAADF) STEM images. The elemental mapping (Fig. 1d) reveals the distribution of the constituent elements in the selected areas. The line-scanning analysis in Fig. 1e also demonstrates the different compositions of these two regions, further demonstrating the phase separation of the produced W-Ir-B alloy. Moreover, the high-resolution TEM (HRTEM)

image (Fig. 1f) shows clear lattice fringes for the IrW and W_2B phases with a well-bonded interface.

Evaluation of oxygen evolution activity and stability. Upon forming the IrW-W_2B biphasic structure, we evaluated the OER catalytic activity and stability of the structure as a catalyst in acidic media. The prepared catalyst was investigated using a three-electrode system in 0.5 M H_2SO_4 aqueous electrolyte. For comparison, commercial powdery IrO_2 and Ir/C (20 wt%) catalysts were also tested under the same conditions. The catalytic activity and stability of the electrodes toward OER were first recorded by linear sweep voltammetry (LSV) and cyclic voltammetry (CV). Fig. 2a shows the linear polarization curves of each catalyst before and after 5,000 cycles. The initial W-Ir-B alloy (i.e., IrW-W_2B biphasic structure) catalyst exhibits superior OER activity with a low overpotential of ~291 mV at the current density of 10 mA cm^{-2}_{geo} (current normalized to the geometric surface area of the electrode), which is ~11 mV and ~23 mV lower than those of commercial IrO_2 and Ir/C catalysts, respectively. After a 5,000-cycle CV test, the W-Ir-B alloy catalyst presents a nearly unchanged linear polarization curve. In sharp contrast, positive shifts are observed for the IrO_2 and Ir/C catalysts, demonstrating the superior activity and stability of the produced W-Ir-B alloy catalyst. The corresponding Tafel slopes are further analyzed as shown in Fig. 2b. The W-Ir-B alloy sample exhibits a Tafel slope of ~78 mV dec^{-1}, similar to commercial IrO_2 (82 mV dec^{-1}) and lower than that of Ir/C (93 mV dec^{-1}). After cycling, the activity of the W-Ir-B alloy catalyst remains constant while the IrO_2 and Ir/C catalysts show a markedly increase of the Tafel slope to 101 and 116 mV dec^{-1}, respectively, further manifesting the stable OER performance of the W-Ir-B alloy catalyst under acidic condition. Additionally, electrochemically active areas (ECSAs) of these catalysts are calculated

from double-layer capacitance according to the CV in a non-Faradaic region (Supplementary Fig. 4). The Ir-W-B alloy catalyst exhibits higher ECSA than the commercial catalysts, demonstrating that the alloy catalyst can effectively expose more active sites to improve the electrocatalytic activity and thus the OER performance, consistent with the LSV results. The specific activity normalized to the surface Ir mass in Supplementary Fig. 5 further estimates the intrinsic activity of the W-Ir-B alloy catalyst. Specifically, the alloy catalyst can reach an Ir mass-normalized current of 518 mA mg_{Ir}^{-1} with a surface Ir loading of ~78.9 μg cm^{-2} at an overpotential of 300 mV, which is higher or comparable to the state-of-the-art Ir-based materials reported to date (Supplementary Table 1), further confirming the high OER activity.

To further compare the long-term OER stability of the different catalysts in acid media, *V-t* relationships are recorded by chronopotentiometry measurements at 100 mA cm^{-2}_{geo}. As shown in Fig. 2c, the W-Ir-B alloy catalyst can hold a nearly constant potential for at least 120 h (recorded every 24 h) at the current density of 100 mA cm^{-2}_{geo}. For the commercial IrO_2 and Ir/C (Supplementary Fig. 6), the efficacy loss can be due to the catalyst detachment resulting from the dissolution of the carbon support and the drastic attack of the continuous O_2 bubbles. This result also proves the superiority of the self-supported W-Ir-B alloy catalyst operated under a harsh acidic environment and it can directly serve as a promising electrode for a robust PEM-based water electrolyzer. The similar CVs taken periodically during the 120-h test further indicate the regeneration of activity, namely electro-stability, in the acidic electrolyte (inset in Fig .2c). It should be noted that even after an 800-h continuous OER test, the W-Ir-B alloy catalyst can also keep an ultrastable activity (Supplementary Fig. 7). Supplementary Table 2 summarizes the stability data of the W-Ir-B alloy catalyst, in comparison with the previously reported Ir-based OER catalysts in the acid electrolytes.

To our best knowledge, the produced W-Ir-B alloy catalyst is one of the most stable OER catalysts in acidic media, and its activity with an overpotential lower than 300 mV at 10 mA cm^{-2}_{geo} also belongs to the top-performing OER catalyst group. In Fig. 2d, the activity and stability for the above three catalysts were further evaluated from the polarization curves before and after CV cycling. At the current density of 10 and 100 mA cm^{-2}_{geo}, the W-Ir-B alloy catalyst exhibits the lowest activity decay (i.e., 0.006% and 0.019% versus the initial state, respectively) after the 5,000 CV cycles as compared with commercial IrO_2 and Ir/C catalysts. Moreover, according to the chronopotentiometry test result, the W-Ir-B alloy catalyst processes a negligible degradation rate of ~4 μV h^{-1} in overpotential during the 800-h OER test at 100 mA cm^{-2}_{geo}. The obtained value is much lower than the state-of-the-art specification of degradation rate (14 μV h^{-1}) for PEM electrolyzers in practical application[27], strongly demonstrating the superiority of the active and ultrastable W-Ir-B alloy catalyst for PEM electrolyzer. To exclude the influence of the amount of involved active sites, surface area or catalyst loading, we also introduce a metric of "stability number (S-number, defined as the ratio between the amount of evolved oxygen and the amount of dissolved Ir[28])" to estimate the technological relevance of the OER catalysts, providing an illustrative comparison for the stability of various reported materials. Based on the dissolution amount of the Ir element according to the inductively coupled plasma-optical emission spectrometer (ICP-OES) data as well as the measured and calculated value of evolved oxygen volume[29], the S-number variation for the produced Ir-W-B alloy catalyst under the current density of 100 mA cm^{-2}_{geo} (potential: ~1.58 V vs. RHE) during the 120-h acidic OER process is calculated. As shown in Supplementary Fig. 8, during the long-term acidic electrolysis, the S-number of the alloy catalyst increases as the reaction time prolongs, resulting from the ultralow dissolution rate of the Ir. The S-

numbers of the alloy catalyst during the 120 h-OER are calculated to be in the range of 5×10^4 to 2.49×10^5, which are comparable or superior to the recently reported IrO_2-based catalysts such as $Ir_{0.7}Sn_{0.3}O_x$ nanoparticles (1.6×10^5)[30], Ir or Ir oxides ($1\sim9.2\times10^5$)[28], $IrO_2@TiO_2$ nanoparticles (1.0×10^4)[31], and $Ir_{0.5}Ti_{0.5}O_x$ (1.5×10^6)[32]. It is also noted that considering the high current density and the super-long duration time (i.e., 120 h), the stability of the alloy catalyst during OER is further highlighted, demonstrating that the produced biphasic alloy can be used as a promising catalyst for acidic OER with both high activity and excellent stability. Toward industrial applications, more strict criteria should be considered. For this reason, we further investigated the OER activity and stability of the W-Ir-B alloy catalyst under larger current densities. Fig. 2e presents the overpotential variations at different current densities ranging from 500 mA cm^{-2}_{geo} to 2 A cm^{-2}_{geo} during a 12-h OER stability test. It turns out that the overpotentials keep stable but oscillate as the increase of the current densities due to the delayed diffusion. Notably, as the current density increases up to 2 A cm^{-2}_{geo}, the overpotential still maintains a low value of ~497 mV (inset in Fig. 2e), which further demonstrates the excellent stability and activity of the catalyst with great potential for application in PEM water electrolyzer under industrial conditions.

In-depth understanding of acidic OER stability. Given the excellent OER stability, a puzzle naturally arises whether any part of the W-Ir-B alloy plays a sacrificing role in ensuring the electrochemical stability of the surface active sites. To elucidate the puzzle, we probed the dissolution behavior of the catalyst during the acidic OER, which employed an ICP-OES to quantify the element dissolution into the H_2SO_4 electrolyte (Fig. 2f). The measurement indicates that trace amounts of Ir are dissolved (up to 0.11 mg L^{-1}) in the acidic electrolyte along with the 120-h stability test, one-order magnitude

lower than the W leaching (up to 3.25 mg L^{-1}) into the electrolyte. Moreover, the Ir and W are dissolved continuously as a logarithmic function of the reaction time (Supplementary Fig. 9), showing the self-stabilization of the alloy catalyst. Supplementary Fig. 10 also presents the dissolution amount of the Ir in comparison with the initial Ir mass. Only 2.49% Ir is dissolved from the surface during the 120-h OER test, further demonstrating the stability. These results exhibit a win-win strategy for electrocatalytic activity and stability, which be ascribed to the catalyst-support interactions.

Prior to our discussion on the catalyst-support interactions, we have to confirm whether the W_2B phase in biphasic W-Ir-B alloy can be corroded during OER to form IrW nanochannels as we designed. To this end, X-ray photoelectron spectroscopy (XPS) concentration-depth profiling and electron microscopy observation were performed to investigate the variation of the composition and microstructure of the catalyst surface after a long-term oxygen production. Fig. 3a depicts the XPS sputter depth profiles of Ir, W, B, and O for the W-Ir-B alloy before and after the OER catalysis (2 h and 120 h). For the as-prepared alloy catalyst, the constituent element contents are constant across the entire sputter depth. Even after 120-h acidic electrocatalysis, the leaching amounts of the constituent elements are not that significant, demonstrating that the generated Ir oxide-based active sites are rather stable during the long-term acidic electrocatalysis. Specifically, the increase of O content from ~20 to ~60 at% reflects the formation of abundant active Ir-based oxides. The profile also indicates that a thin layer of ~200 nm (marked by a red dotted rectangle) is both active and stable for the acidic OER, in accordance with the dissolution behavior in Fig. 2f. Such a finding was further proven by the SEM characterization in Fig. 3b. The surface and cross-sectional images of the alloy catalyst after the 120-h acidic OER present a nanoporous channel structure with

a thickness of ~200 nm. Looking into the specific size, we can recognize that the bi-continuous channel structure possesses an average width of ~100 nm, approximately in correspondence to the separated phase of IrW in the bare W-Ir-B alloy (Supplementary Fig. 1). As demonstrated by the surface evolution of the W-Ir-B sample (Supplementary Fig. 11), the interconnected W_2B phase with lower electrocatalytic activity (see Fig. 2a) is preferentially dissolved in the acidic electrolyte during the incipient electrochemical reaction, resulting in the formation of an IrW channel structure with nanopores. After the 120-h long-term acidic OER, the nanochannel structure is almost unchanged in comparison with the 2-h reacted one (Supplementary Fig. 12), further demonstrating the electrochemical stability. To gain more information for the active oxides on surface after the long-term OER, we collected the TEM image and the SAED pattern on the surface oxides (Fig. 3c), showing a polycrystalline channel structure with a width of ~100 nm. STEM elemental mapping and EDS analysis (Fig. 3d and Supplementary Fig. 13) further reveal a uniform distribution of Ir, W, and O elements. These observations confirm that the Ir oxide species are rooted on the IrW nanochannels. The Ir-O layer formed on surface can serve as the dominant active site, playing an important role in the activity and stability of the catalyst in the subsequent OER electrocatalysis[33].

It is worth mentioning that the choice of elemental compositions is critical to the OER stability as alloy catalysts are inherently stable in acidic electrolytes. As we know, Ru is more OER active but less stable than Ir, which in turn affects the OER stability of alloy catalysts. For comparison, a W-Ru-B alloy rod electrode with the same configuration (diameter, composition, fabrication method, etc.) was used as an electrode for OER in 0.5 M H_2SO_4 electrolyte. The W-Ir-B alloy catalyst shows slightly lower OER activity but significantly higher stability than that of the W-Ru-B alloy catalyst (<6 h) in acidic media (Supplementary Fig. 14). The W-Ru-B alloy electrode is

gradually dissolved during the long-term OER test, which finally turns the H_2SO_4 electrolyte brownish-black (Supplementary Fig. 15). This also infers that improving catalyst stability is a more challenging task than achieving electrocatalytic activity for PEM application.

Given the importance of elemental compositions, it is imperative to further assess the element contribution during the acidic OER. To this end, the surface chemical states and valence status of the constituent elements were investigated by high-resolution XPS. Fig. 4a shows the Ir 4*f* spectra of the bare W-Ir-B alloy surface, the activated surface (formed during 6-CV scans), the catalyst surfaces after 2-h and 120-h OER chronoamperometric test. The Ir 4*f* peaks of the bare W-Ir-B alloy surface are centered around 60.9 ($4f_{7/2}$) and 63.9 eV ($4f_{5/2}$), corresponding to the metallic Ir. Notably, trace Ir is oxidized on the alloy surface inevitably. It is also recognized that the Ir 4*f* binding energies are positively shifted with 0.1~0.2 eV in comparison with the standard Ir element[34], resulting from the high electronegativity difference between W and Ir[35, 36], which may favor the electrocatalytic activity of Ir sites. After the alloy electrode is anodically treated, a significant change is observed for the Ir XPS spectra. The surface metallic Ir tends to form Ir oxides during the anodic polarization. Specifically, the oxidation state of the Ir can be assigned to the Ir^{IV} and Ir^{III} species along with their respective satellites[37], which are located at 61.8 eV (64.8 eV) and 62.2 eV (65.2 eV), respectively. The formation of the intermediate Ir^{III} may undergo two pathways[38]: the metallic Ir^0 is directly dissolved to form soluble Ir^{III} and further IrO_2, or transforms into Ir^{III} through the decomposition of $Ir^{IV}O_2OH$ with the release of O_2 and formation of surface $HIr^{III}O_2$ intermediates. Similar features have been observed in the Ir-Ni[6], Ir-Sn[39], and Ir-Ti[32] systems. Moreover, the presence of the electronic defect-liked Ir^{III} intermediates with lower coordination numbers can also contribute to the

electrocatalytic activity of the alloy catalyst[37, 40], resulting in an enhanced efficiency toward OER. After the 2-h OER test in the acidic electrolyte, the metallic Ir on the surface is completely transformed into the Ir^{IV} and Ir^{III}. Moreover, A significant change in the peak area of the Ir^{IV} and Ir^{III} species can be observed in comparison with the anodically treated electrode after 6 scans and 2 h as a large amount of stable Ir^{IV} is formed during the OER process[38]. Remarkably, in the next 120-h OER test, there are no obvious changes in the binding energies and the peak areas of the Ir^{IV} and Ir^{III} species, demonstrating that the active Ir oxides formed in the IrW nanochannels are rather stable during the long-term stability test, consistent with the ICP-OES data.

As a matter of fact, various pathways have been reported for the Ir degradation during OER in literature. As outlined in a recent review[41], the dissolution of Ir species could be detected by *in-situ* measurement of ^{18}O-labeled samples. Moreover, Casalongue et al. observed Ir^{V} surface species by *in-situ* ambient-pressure XPS[35]; however, Pfeifer et al. pointed out that it should be Ir^{III} species[37, 42]. In parallel, Minguzzi et al. reported the presence of Ir^{III} and Ir^{V} species based on X-ray absorption near edge structure spectroscopy (XANES)[43]. While Kasian et al. proposed that the $Ir^{III}OOH$ directly contributes to the oxygen evolution from the lattice by using ^{16}O-containing oxides[40], the degradation of Ir to a higher oxidation state (>IV) has been observed based on the *in-situ* XANES studies of Ir films[44], RuIr oxides[45], and Ruthenate-Iridate pyrochlores[46]. Furthermore, as indicated by Kasian et al., the dissolution of Ir is related to the potential; as the potential is sufficiently high, further oxidation of Ir^{V} or Ir^{VI} is preferable[38]. Thus the pathways proposed for the OER-triggered dissolution of Ir in literature are mainly the formation of soluble Ir^{III} or Ir(>IV) species. According to our XPS spectra and ICP-OES result (Supplementary Fig. 11), the oxidation and dissolution of Ir are inevitable, especially at the initial OER stage. To

enable the long-term stability of the catalyst, it is imperative to form a stable structure that hinders the further formation of the unstable Ir^{III} or Ir^{VI} species. In our case, the observed IrW nanochannel-support layer with specific particle size is an ideal configuration to achieve such a goal. We then characterize the W 4*f* spectra during OER (Fig. 4b). The XPS-peak-differentiation result reveals the coexistence of metallic W^0 and W^{VI}, demonstrating that the metallic W^0 state becomes oxidized (WO_3) during the OER process. The hypervalent oxidation of W can adsorb redundant O atoms during electrocatalysis and prevent active Ir oxides from further oxidizing to higher valence (e.g., the unstable Ir^{VI} species) and subsequently dissolving in acid. Fig. 4c further shows the O1*s* XPS spectra, increasing intensities from the growth of oxygen species are observed during the acidic OER electrocatalysis. The fitting of the O 1*s* spectra reveals up to 4 different oxygen species of the catalyst after the OER stability tests. The peaks at 532.8 and 531.5 eV can be assigned to adsorbed water and hydroxyl groups, respectively[6]. For distinguishing the lattice oxygen that binds to different metals, the species at 530.8 eV can be recognized to the W-O whereas the peak appearing around 530.0 eV should be assigned to the Ir-O[16, 47]. In this state, the W elements with lower electronegativity are expected to bind oxygen intermediates at the very start, and then the *in-situ* formed IrW support can effectively suppress the further electron loss and the oxidation of surface active Ir oxides to soluble Ir, an intermediate blaming for catalytic deterioration[48].

Although W_2B is not electrocatalytically active for OER (Fig. 2a), it is worthy of affirming the contribution of the B element to OER performance. The detected B 1*s* XPS spectra can be deconvoluted to two peaks at around 187.9 and 191.8 eV (Supplementary Fig. 16). The former is ascribed to the interaction of B with W while the latter can be assigned to the boron-oxo species[49, 50]. Along with the acidic

electrocatalysis process, the B elements with low-valence tend to be oxidized so that most of them are dissolved. Nevertheless, the residual electron-deficient B in the alloy substrate could generate a certain affinity to surface metal atoms to provide extra binding sites such as metal-B-O bonds[51, 52], which also contributes to the superior OER activity. For comparison, we also prepared two W-Ir catalysts with different Ir contents for acidic OER activity and stability evaluation (Supplementary Fig. 17), which shows that the W-Ir-B alloy catalyst exhibits an optimal OER performance with the minimum usage of 20 at%. These results demonstrate that the addition of B makes a credible contribution to reducing the Ir content and positively promoting the electrocatalytic activity. More importantly, our theoretical calculation indicates that the work functions of IrW and W_2B surface are 4.467 and 3.915 eV, respectively (Supplementary Fig. 18). Both the Ir and W contribute to the electron density of IrW (002) surface around the Fermi energy level, while the electron density of W_2B (211) surface is mainly offered by W atoms. The large number of electrons near the Fermi level indicates that both the IrW (002) surface and the W_2B (211) surface are metallic, allowing efficient electron transfer. As such, the introduction of B is essential to form a biphasic alloy structure, in which the phase W_2B with lower work function tends to lose electron[53] and can be preferentially dissolved during the acidic OER, thereby forming the IrW nanochannel structure. It should also be noted that in comparison with previously reported glassy carbon-supported IrW catalysts directly which are fabricated by wet chemistry methods[15, 16, 26], the IrW nanochannels with free-standing structure *in-situ* formed through the corrosion of the biphasic IrW-W_2B (W-Ir-B) alloy during the acidic OER process exhibit unique stability in acidic media except for the comparable activity. Such long-term stability under harsh acidic condition is strongly dependent on the specific particle size of the supported IrW (i.e., 200 nm thickness). The good balance for activity

and stability of the IrW nanochannel support reveals its potential and reliability for application in PEM electrolyzer from the perspective of engineering.

Theoretical investigation. Upon acquiring the key information, we are now in a position to understand the support effect of the IrW nanochannels by theoretical calculations. Firstly, to study the interfacial electron-transfer process, we simulated the differential charge densities of the IrO_2 clusters adsorption on the IrW (002) surface (Fig. 5a and Supplementary Fig. 19). With the support of the IrW, the charge distribution of the Ir and O atoms is altered. Specifically, Ir atoms lose electrons while O atoms gain electrons. As a result, the accumulation of positive charges at Ir atoms could promote the formation of the catalytically active Ir-O intermediates[54, 55], thereby boosting the OER performance. We further investigated the binding structures of Ir-O intermediates by simulating the adsorption of the $(IrO_2)_n$ (n=1, 2, 4…) clusters on the IrW support. For the IrO_2 (n=1) cluster, the binding energies of the adsorbed O-Ir at different sites are calculated to be -6.03, -6.17 and -5.50 eV, weaker than that of the single IrO_2 molecule (-6.91 eV) (Fig. 5b). This suggests that the bond strength of O-Ir is reduced by the IrW substrate, due to the electronic interaction between the catalyst and support[19]. When n=2, a similar result was obtained; the binding energies of the IrW-supported O-Ir at different sites are mostly lower than those of free $(IrO_2)_2$ (Fig. 5c). However, the O-Ir bonds tend to be broken when the cluster size further increases to n=4 (Fig. 5d). Combined with the differential charge density maps, the binding energies indicate that the IrW support can prevent the surface Ir atoms from accumulating more O atoms to form unstable Ir^{III} or Ir^{VI} species as well as retain a thin layer of electrocatalytically active IrO_2 clusters, thereby maintaining both high activity and stability in the acidic catalytic process. Such a support-stabilizing effect can perfectly

work in long term as the IrW is thick enough (at least 200 nm) to balance activity and stability.

Discussion

In summary, we have developed a biphasic W-Ir-B alloy catalyst for high-performance acidic OER in light of the support-stabilizing effect in PEM electrolyzers. The as-prepared catalyst with *in-situ* formed IrW nanochannels exhibits a low and stable overpotential of ~497 mV to achieve a high current density of 2 A cm^{-2}_{geo}, which maintains at least 800 h with negligible overpotential degradation at 100 mA cm^{-2}_{geo}. Through surface structure analyses and DFT calculations, we have revealed that the IrW support can firm the surface adsorbed active IrO_2, preventing the Ir from accumulating more O and leveling the valence state. Essentially, this catalyst-support interaction retains a thin layer of electrocatalytically active IrO_2 clusters layer, performing excellent activity and ultra-stability toward the acidic OER. This work not only provides a win-win alternative for industrial PEM-based electrolyzer with high activity and stability, but also enlightens the further exploration and design of high-efficiency electrocatalysts based on support-stabilizing effect for other energy conversion applications.

Methods

Material fabrication. The W-Ir-B alloy ingots with different components were prepared by arc-melting high-purity elements (> 99.9 wt%) under a Ti-gettered argon atmosphere. The alloy ingots were re-melted at least 5 times to ensure chemical homogeneity. Then the W-Ir-B cylindrical rods with a diameter of 1 mm were fabricated by drop-casting of alloy melt into a copper mold under a high purity argon atmosphere.

Structural characterizations. Phase identifications of the as-cast samples were determined by X-ray diffraction (XRD, Rigaku DMAX-RB-12KW, Cu-Kα). Microstructures and chemical compositions of the samples were characterized using scanning electron microscopy (SEM, Zeiss Auriga, Germany) equipped with an energy dispersive X-ray spectrometer (EDS) and transmission electron microscope (TEM, Tecnai G2 F20). X-ray photoelectron spectroscopy (XPS, AXIS-ULTRA-DLD, Kratos) with an Al Kα (mono, 1486.6 eV) anode at an energy level of 150 W in a vacuum of 10^{-7} Pa was employed to investigate the surface chemical states and binding energies of the samples. Individual spectrums were corrected with the reference to C 1*s* binding energy of eV and then deconvoluted using Gaussian fitting. The contents of various elements including W, Ir, and B were determined by inductively coupled plasma-optical emission spectrometer (ICP-OES, Prodigy, Leeman).

Electrochemical measurements. The electrochemical tests were performed using an electrochemical workstation (CHI 660D) with a typical three-electrode system at room temperature, where a standard Ag/AgCl electrode, a Pt plate, and the W-Ir-B alloy rod with a fixed size of Φ 1 mm × 1 cm were used as the reference electrode, counter electrode, and working electrode, respectively. For the contrastive Ir/C (20 wt%) and IrO_2 working electrodes, the Ir/C and IrO_2 catalyst inks were loaded on a glassy carbon electrode (GCE), respectively. The overall catalyst loading amount was about 0.25 mg cm^{-2}. All the electrochemical measurements were performed in an acidic electrolyte of 0.5 M H_2SO_4 aqueous solution. Linear scan voltammetry (LSV) was conducted at a scan rate of 1 mV s^{-1} and corrected for *iR* losses (according to the solution resistance). Before each LSV test, the catalyst electrodes were conducted with numbers of cyclic

voltammetry (CV) cycles until a stable CV curve was obtained. For convenience, all potentials in this study were converted to RHE by the Nernst equation of $E_{RHE} = E_{Ag/AgCl} + 0.197 + 0.0591 \times pH$ and the overpotential (η) for OER was calculated using the equation of $\eta = E_{RHE} - 1.23$. The electrochemical impedance spectroscopy was collected at 1.4 V vs RHE from 10^5 to 0.1 Hz with an amplitude of 10 mV for the calculation of the solution resistance. The stability performances for the catalysts were recorded by chronopotentiometry measurements, where constant current densities in the range of 10 mA cm^{-2}_{geo} to 2 A cm^{-2}_{geo} were provided. Electrochemical double-layer capacitance measurements were used to determine the electrochemically active surface areas (ECSAs) of the electrocatalysts at non-faradaic overpotentials. By plotting the difference of current density against the scan rate, a linear trend was observed. The slope of the fitting line is equal to twice of the double-layer capacitance, which is proportional to the ECSA of the catalysts. The amount of produced oxygen was measured in real-time by a NeoFox Sport Oxygen Sensor (Ocean Optics).

Theoretical calculation. The XRD results show that the diffraction angles of the IrW (002) surface and the W_2B (211) surface are close to each other. Therefore, we constructed the IrW (002) surface and W_2B (211) surface based on the optimized bulk lattice parameters. All geometric optimizations and density of states calculations were performed based on the density functional theory (DFT) method with the Vienna Ab-initio Simulation Package (VASP)[56, 57]. The exchange-correlation energy was described by the generalized gradient approximation (GGA) proposed by Perdew, Burke, and Ernzerhof (PBE) with a cut-off energy of 500 eV[58-60]. The IrW (002) substrate was constructed with a 5×3 supercell, in which the dimensional parameters of a and b directions are 13.47 Å and 13.94 Å, respectively. The W_2B (211) substrate was

constructed with a 2×3 supercell, in which the dimensional parameters of a and b directions are 18.36 Å and 14.73Å, respectively. The Brillouin zone of IrW (002) and W_2B (211) were sampled by Gamma centered 3 × 3 × 1 and 2 × 2 × 1 k-points, respectively. A vacuum layer of 20 Å was adopted in the z-direction to avoid the interaction between periodic layers. The van der Waals interactions were described using the empirical correction in Grimme's scheme[61]. The convergence criterion for the energy and the maximum force for the optimization was set to 10^{-5} eV and 0.05 eV $Å^{-1}$, respectively. The substrates of both IrW (002) surface and W_2B (211) surface consist of two layers. The bottom layer of the surface models was kept fixed to hold the characteristics of realistic surfaces, while the atoms of the top layer of the unit cells were allowed to be fully relaxed during the geometry optimizations. For the $(IrO_2)_n$ cluster, the equation for calculating the binding energy of the O atom is $E_b = E(Ir_nO_{2n-1}) + E(O) - E(Ir_nO_{2n})$, where $E(Ir_nO_{2n})$ is the total energy of the optimized $(IrO_2)_n$ cluster, $E(Ir_nO_{2n-1})$ represents the energy of $(IrO_2)_n$ cluster after losing one O atom, and E(O) represents the energy of O atom. For the $(IrO_2)_n$ cluster adsorbed on IrW (002) substrate, the calculation formula is $E_b = E(substrate@Ir_nO_{2n}) - E(substrate@Ir_nO_{2n-1}) - E(O)$, where $E(substrate@Ir_nO_{2n})$ is the total energy of the optimized $(IrO_2)_n$ cluster adsorbed on the IrW (002) substrate, $E(substrate@Ir_nO_{2n-1})$ represents the energy of an $(IrO_2)_n$ cluster after adsorbed on the substrate and losing an O atom, and E(O) represents the energy of O atom[62, 63].

Data availability

Source data are provided with this paper.

References

1. Faustini, M. *et al.* Hierarchically structured ultraporous iridium-based materials: A novel catalyst architecture for proton exchange membrane water electrolyzers. *Adv. Energy Mater.* **9**, 1802136 (2019).
2. Carmo, M. *et al.* PEM water electrolysis: Innovative approaches towards catalyst separation, recovery and recycling. *Int. J. Hydrogen Energy* **44**, 3450-3455 (2019).
3. Oener, S.Z., Foster, M.J. & Boettcher, S.W. Accelerating water dissociation in bipolar membranes and for electrocatalysis. *Science* **369** 1099-1103 (2020).
4. Kumar, S.S. & Himabindu, V. Hydrogen production by PEM water electrolysis-A review. *Mater. Sci. Energy Technol.* **2**, 442-454 (2019).
5. Hosseini, S.E. & Wahid, M.A. Hydrogen from solar energy, a clean energy carrier from a sustainable source of energy. *Int. J. Energy Res.* **44**, 4110-4131 (2020).
6. Reier, T. *et al.* Molecular insight in structure and activity of highly efficient, low-Ir Ir–Ni oxide catalysts for electrochemical water splitting (OER). *J. Am. Chem. Soc.* **137**, 13031-13040 (2015).
7. Ioroi, T., Siroma, Z., Yamazaki, S. & Yasuda, K. Electrocatalysts for PEM fuel cells. *Adv. Energy Mater.* **9**, 1801284 (2019).
8. Shan, J., Zheng, Y., Shi, B., Davey, K. & Qiao, S.Z. Regulating electrocatalysts via surface and interface engineering for acidic water electrooxidation. *ACS Energy Lett.* **4**, 2719-2730 (2019).
9. Shi, Z., Wang, X., Ge, J., Liu, C. & Xing, W. Fundamental understanding on acidic oxygen evolution reaction: Mechanism study and state-of-the-art catalysts. *Nanoscale* **12** 13249-13275 (2020).
10. Reier, T., Oezaslan, M. & Strasser, P. Electrocatalytic oxygen evolution reaction (OER) on Ru, Ir, and Pt catalysts: A comparative study of nanoparticles and bulk materials. *ACS Catal.* **2**, 1765-1772 (2012).
11. Wen, X., Bai, L., Li, M. & Guan, J. Ultrafine iridium oxide supported on carbon nanotubes for efficient catalysis of oxygen evolution and oxygen reduction reactions. *Mater. Today Energy* **10**, 153-160 (2018).
12. Zhu, M., Shao, Q., Qian, Y. & Huang, X. Superior overall water splitting electrocatalysis in acidic conditions enabled by bimetallic Ir-Ag nanotubes.

Nano Energy **56**, 330-337 (2019).

13. Xu, J. *et al.* Strong electronic coupling between ultrafine iridium–ruthenium nanoclusters and conductive, acid-stable tellurium nanoparticle support for efficient and durable oxygen evolution in acidic and neutral media. *ACS Catal.* **10**, 3571-3579 (2020).
14. Kwon, T. *et al.* Cobalt assisted synthesis of IrCu hollow octahedral nanocages as highly active electrocatalysts toward oxygen evolution reaction. *Adv. Funct. Mater.* **27**, 1604688 (2017).
15. Fu, L., Hu, X., Li, Y., Cheng, G. & Luo, W. IrW nanobranches as an advanced electrocatalyst for pH-universal overall water splitting. *Nanoscale* **11**, 8898-8905 (2019).
16. Gao, J., Huang, X., Cai, W., Wang, Q., Jia, C. & Liu, B. Rational design of an iridium-tungsten composite with an iridium-rich surface for acidic water oxidation. *ACS Appl. Mater. Interfaces* **12**, 25991-26001 (2020).
17. Lu, X. & Zhao, C. Electrodeposition of hierarchically structured three-dimensional nickel-iron electrodes for efficient oxygen evolution at high current densities. *Nat. Commun.* **6**, 6616 (2015).
18. Yu, X., Wang, M., Gong, X., Guo, Z., Wang, Z. & Jiao, S. Self-supporting porous CoP-based films with phase-separation structure for ultrastable overall water electrolysis at large current density. *Adv. Energy Mater.* **8**, 1802445 (2018).
19. Oh, H.S. *et al.* Electrochemical catalyst-support effects and their stabilizing role for IrO_x nanoparticle catalysts during the oxygen evolution reaction. *J. Am. Chem. Soc.* **138**, 12552-12563 (2016).
20. Wu, X. *et al.* Metal-support interaction boosted electrocatalysis of ultrasmall iridium nanoparticles supported on nitrogen doped graphene for highly efficient water electrolysis in acidic and alkaline media. *Nano Energy* **62**, 117-126 (2019).
21. Ohno, H., Nohara, S., Kakinuma, K., Uchida, M. & Uchida, H. Effect of electronic conductivities of iridium oxide/doped SnO_2 oxygen-evolving catalysts on the polarization properties in proton exchange membrane water electrolysis. *Catalysts* **9**, 74 (2019).
22. Wang, L. *et al.* Nanostructured Ir-supported on Ti_4O_7 as a cost-effective anode for proton exchange membrane (PEM) electrolyzers. *Phys. Chem. Chem. Phys.* **18**, 4487-4495 (2016).
23. Karimi, F. & Peppley, B.A. Metal carbide and oxide supports for iridium-based

oxygen evolution reaction electrocatalysts for polymer-electrolyte-membrane water electrolysis. *Electrochim. Acta* **246**, 654-670 (2017).

24. Hartig-Weiss, A. *et al.* Iridium oxide catalyst supported on antimony-doped tin oxide for high oxygen evolution reaction activity in acidic media. *ACS Appl. Nano Mater.* **3**, 2185-2196 (2020).
25. Geiger, S., Kasian, O., Mingers, A.M., Mayrhofer, K.J.J. & Cherevko, S. Stability limits of tin-based electrocatalyst supports. *Sci. Rep.* **7**, 4595 (2017).
26. Lv, F. *et al.* Iridium-tungsten alloy nanodendrites as pH-universal water-splitting electrocatalysts. *ACS Central Sci.* **4**, 1244-1252 (2018).
27. Spoeri, C., Kwan, J.T.H., Bonakdarpour, A., Wilkinson, D.P. & Strasser, P. The stability challenges of oxygen evolving catalysts: towards a common fundamental understanding and mitigation of catalyst degradation. *Angew. Chem. Int. Edit.* **56**, 5994-6021 (2017).
28. Geiger, S. *et al.* The stability number as a metric for electrocatalyst stability benchmarking. *Nat. Catal.* **1**, 508-515 (2018).
29. Hu, F. *et al.* Amorphous metallic NiFeP: A conductive bulk material achieving high activity for oxygen evolution reaction in both alkaline and acidic media. *Adv. Mater.* **29**, 1606570 (2017).
30. Kasian, O., Geiger, S., Mayrhofer, K.J. & Cherevko, S. Electrochemical on line ICP-MS in electrocatalysis research. *Chem. Rec.* **19**, 2130-2142 (2019).
31. Van-Pham, C. *et al.* IrO_2 coated TiO_2 core-shell microparticles advance performance of low loading proton exchange membrane water electrolyzers. *Appl. Catal. B: Environ.* **269**, 118762 (2020).
32. Kasian, O. *et al.* Stabilization of an iridium oxygen evolution catalyst by titanium oxides. *J. Phys. Energy* (2020).
33. Li, T. *et al.* Atomic-scale insights into surface species of electrocatalysts in three dimensions. *Nat. Catal.* **1**, 300-305 (2018).
34. Chastain, J., King Jr, R.C., Handbook of X-ray photoelectron spectroscopy. *Perkin-Elmer Corporation* **40**, 221 (1992).
35. Sanchez-Casalongue, H.G., Ng, M.L., Kaya, S., Friebel, D., Ogasawara, H. & Nilsson, A. In situ observation of surface species on iridium oxide nanoparticles during the oxygen evolution reaction. *Angew. Chem.* **126**, 7297-7300 (2014).
36. Wang, D. *et al.* Electronic behaviour of Au-Pt alloys and the 4f binding energy shift anomaly in Au bimetallics-X-ray spectroscopy studies. *AIP Adv.* **8**, 065210

(2018).

37. Pfeifer, V. *et al.* The electronic structure of iridium oxide electrodes active in water splitting. *Phys. Chem. Chem. Phys.* **18**, 2292-2296 (2016).

38. Kasian, O., Grote, J.P., Geiger, S., Cherevko, S. & Mayrhofer, K.J. The common intermediates of oxygen evolution and dissolution reactions during water electrolysis on iridium. *Angew. Chem. Int. Edit.* **57**, 2488-2491 (2018).

39. Kasian, O. *et al.* Using instability of a non-stoichiometric mixed oxide oxygen evolution catalyst as a tool to improve its electrocatalytic performance. *Electrocatalysis* **9**, 139-145 (2018).

40. Kasian, O. *et al.* Degradation of iridium oxides via oxygen evolution from the lattice: correlating atomic scale structure with reaction mechanisms. *Energ. Environ. Sci.* **12**, 3548-3555 (2019).

41. Reier, T., Nong, H.N., Teschner, D., Schlögl, R. & Strasser, P. Electrocatalytic oxygen evolution reaction in acidic environments-reaction mechanisms and catalysts. *Adv. Energy Mater.* **7**, 1601275 (2017).

42. Pfeifer, V. *et al.* The electronic structure of iridium and its oxides. *Surf. Interface Anal.* **48**, 261-273 (2016).

43. Minguzzi, A. *et al.* Observing the oxidation state turnover in heterogeneous iridium-based water oxidation catalysts. *Chem. Sci.* **5**, 3591-3597 (2014).

44. Danilovic, N. *et al.* Activity-stability trends for the oxygen evolution reaction on monometallic oxides in acidic environments. *J. Phys. Chem. Lett.* **5**, 2474-2478 (2014).

45. Danilovic, N. *et al.* Using surface segregation to design stable Ru-Ir oxides for the oxygen evolution reaction in acidic environments. *Angew. Chem. Int. Edit.* **53**, 14016-14021 (2014).

46. Sardar, K. *et al.* Water-splitting electrocatalysis in acid conditions using ruthenate-iridate pyrochlores. *Angew. Chem. Int. Edit.* **53**, 10960-10964 (2014).

47. Abbott, DF. *et al.* Iridium oxide for the oxygen evolution reaction: correlation between particle size, morphology, and the surface hydroxo layer from operando XAS. *Chem. Mater.* **28**, 6591-6604 (2016).

48. Jovanovič, P. *et al.* Electrochemical dissolution of iridium and iridium oxide particles in acidic media: transmission electron microscopy, electrochemical flow cell coupled to inductively coupled plasma mass spectrometry, and X-ray absorption spectroscopy study. *J. Am. Chem. Soc.* **139**, 12837-12846 (2017).

49. Li, H. *et al.* Earth-abundant iron diboride (FeB_2) nanoparticles as highly active bifunctional electrocatalysts for overall water splitting. *Adv. Energy Mater.* **7**, 1700513 (2017).
50. Hu, F. *et al.* Designing highly efficient and long-term durable electrocatalyst for oxygen evolution by coupling B and P into amorphous porous NiFe-based material. **15**, *Small* 1901020 (2019).
51. Qiao, Y. *et al.* Boron-rich environment boosting ruthenium boride on B, N doped carbon outperforms platinum for hydrogen evolution reaction in a universal pH range. *Nano Energy*, **75**, 104881 (2020).
52. Zhang, L. *et al.* One-pot synthesized boron-doped RhFe alloy with enhanced catalytic performance for hydrogen evolution reaction. *Appl. Catal. B: Environ.* **230**, 58-64 (2018).
53. Lang, N. & Kohn, W. Theory of metal surfaces: work function. *Phys. Rev. B* **3**, 1215 (1971).
54. Shan, J. *et al.* Charge-redistribution-enhanced nanocrystalline Ru@IrO_x electrocatalysts for oxygen evolution in acidic media. *Chem* **5**, 445-459 (2019).
55. Görlin, M. *et al.* Tracking catalyst redox states and reaction dynamics in Ni-Fe oxyhydroxide oxygen evolution reaction electrocatalysts: the role of catalyst support and electrolyte pH. *J. Am. Chem. Soc.* **139**, 2070-2082 (2017).
56. Kresse, G. & Furthmüller, J. Efficient iterative schemes for ab initio total-energy calculations using a plane-wave basis set. *Phys. Rev. B* **54**, 11169 (1996).
57. Hafner, J. Ab-initio simulations of materials using VASP: Density-functional theory and beyond. *J. Comput. Chem.* **29**, 2044-2078 (2008).
58. Perdew, J.P., Burke, K. & Ernzerhof, M. Generalized gradient approximation made simple. *Phys. Rev. Lett.* **77**, 3865 (1996).
59. Blöchl, P.E. Projector augmented-wave method. *Phys. Rev. B* **50**, 17953 (1994).
60. Kresse, G. & Joubert, D. From ultrasoft pseudopotentials to the projector augmented-wave method. *Phys. Rev. B* **59**, 1758 (1999).
61. Grimme, S. Semiempirical GGA-type density functional constructed with a long-range dispersion correction. *J. Comput. Chem.* **27**, 1787-1799 (2006).
62. Zhao, C.X., Zhang, G.X., Gao, W. & Jiang, Q. Single metal atoms regulated flexibly by a 2D InSe substrate for CO_2 reduction electrocatalysts. *J. Mater. Chem. A* **7**, 8210-8217 (2019).
63. Tang, Y., Yang, Z. & Dai, X. Trapping of metal atoms in the defects on graphene.

J Chem. Phys. **135**, 224704 (2011).

Acknowledgements

The authors acknowledge financial support from the National Key R&D Program of China (2017YFA0207301, 2018YFA0208603), NSFC (21725102, 91961106, 22078059, 21802103, 21890751), NSF of Guangdong Province of China (2019A1515011788), Project of Educational Commission of Guangdong Province of China (2020ZDZX2050), and Hong Kong Polytechnic University Postdoctoral Fellowships Scheme (No. 1-YW3D).

Author contributions

Y.X., F.H., K.C.C., and X.W. supervised and conceived this project. R.L. carried out materials fabrication, XRD/SEM/TEM/XPS characterizations, and electrochemical measurements. Y.X., F.H., K.C.C., X.W., and R.L. analyzed the results and wrote the paper. H.W. performed the DFT calculations and computational models. J.W., Z.Li, L.Z., and Y.L. contributed to the electrochemical measurements. X.L. and Z.Lu revised the manuscript and triggered helpful discussion. All authors discussed the results and commended the manuscript.

Competing interests

The authors declare no competing interests.

Figures

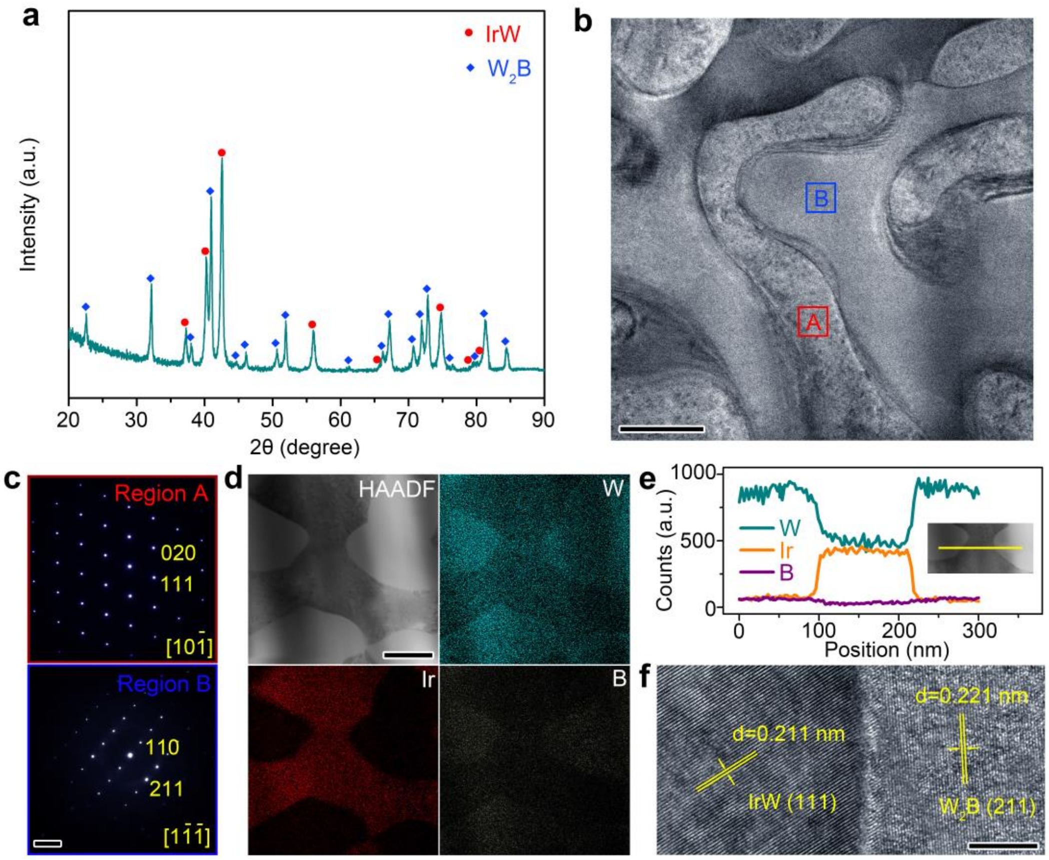

Fig. 1 Characterizations of the W-Ir-B alloy catalyst (atomic ratio, 60:20:20). a XRD patterns of the rapidly solidified alloy rod with a diameter of 1 mm. **b** TEM image of the W-Ir-B alloy. **c** SAED patterns of the different regions marked in Fig. 1b. Region A: IrW phase (red solid box); Region B: W_2B phase (blue solid box). **d** HAADF-STEM image and the corresponding EDS elemental mapping of the W-Ir-B alloy catalyst. **e** STEM-EDS line-scanning profile of the separated phases of the W-Ir-B alloy catalyst. The inset shows the selected area for EDS line-scanning analysis. **f** HRTEM image of the phase-separated W-Ir-B alloy. Scale bar: **b** 100 nm; **c** 5 1/nm; **d** 100 nm; **f** 5 nm.

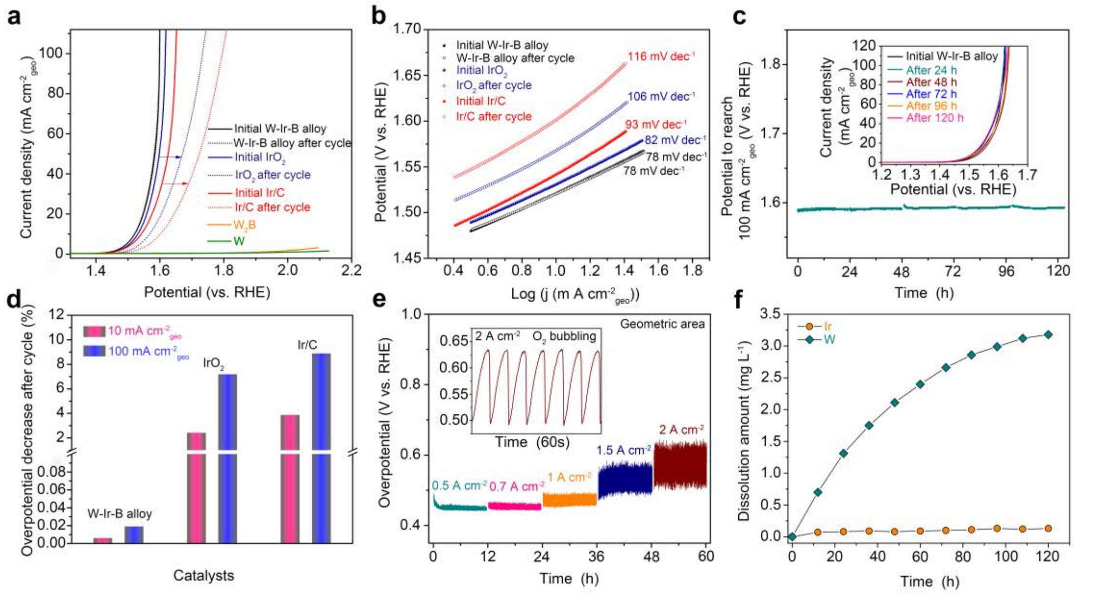

Fig. 2 OER activity and stability comparison. a Linear polarization curves of the W-Ir-B alloy, commercially IrO_2, and Ir/C catalysts before and after 5,000 cycles in 0.5 M H_2SO_4 electrolyte. The LSV curves of the pure W and the W_2B alloy are also included. **b** Tafel slopes of the electrocatalysts before and after 5,000 OER cycles. **c** Chronopotentiometry curves of the W-Ir-B alloy catalyst in 0.5 M H_2SO_4 electrolyte at a current density of 100 mA cm^{-2}_{geo} for 120 h. The inset shows the LSV curves taken periodically during the test (every 24 h). **d** Activity degradations for the three catalysts at the current densities of 10 and 100 mA cm^{-2}_{geo}, respectively. **e** Overpotential variations of the W-Ir-B alloy catalyst at the current densities in the range of 500 mA cm^{-2}_{geo} to 2 A cm^{-2}_{geo}. The inset shows the voltage fluctuation caused by the O_2 bubbling. **f** Dissolution amount variation of Ir and W elements during the 120-h acidic OER test at 100 mA cm^{-2}_{geo}.

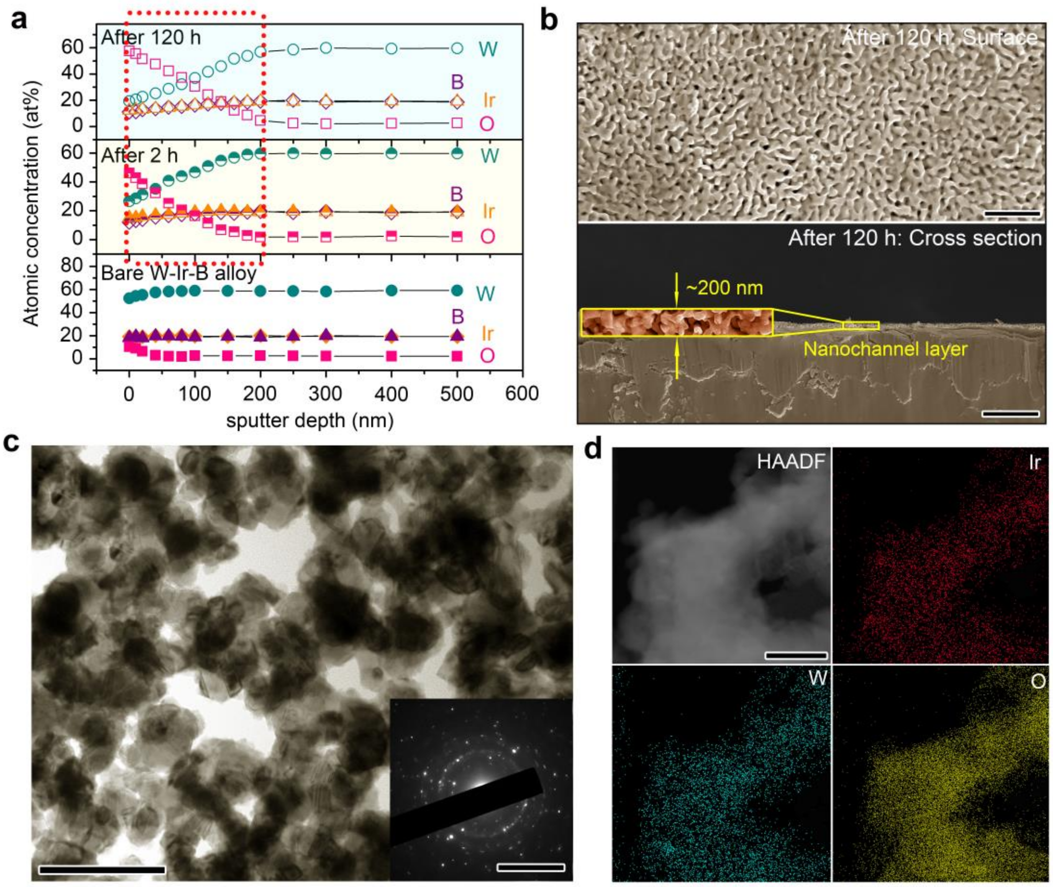

Fig. 3 Surface active structure formed during the acidic OER and the characterizations. a XPS sputter depth profile measurements of the bare W-Ir-B alloy catalyst, the alloy catalyst after 2-h OER test, and the alloy catalyst after 120-h OER test. The red dotted box indicates the depth of the surface active thin layer. **b** Surface and cross-sectional SEM images of the W-Ir-B alloy catalyst after the 120-h acidic OER test. The inset shows the magnified cross-section image of the nanochannel structure (yellow solid box). **c** TEM image of the surface active oxides formed during the 120-h acidic OER. The inset shows the SAED pattern. **d** STEM elemental mapping of the surface nanochannel structure. Scale bar: **b** 500 nm (top), 1 μm (down); **c** 200 nm, 5 1/nm (inset); **d** 100 nm.

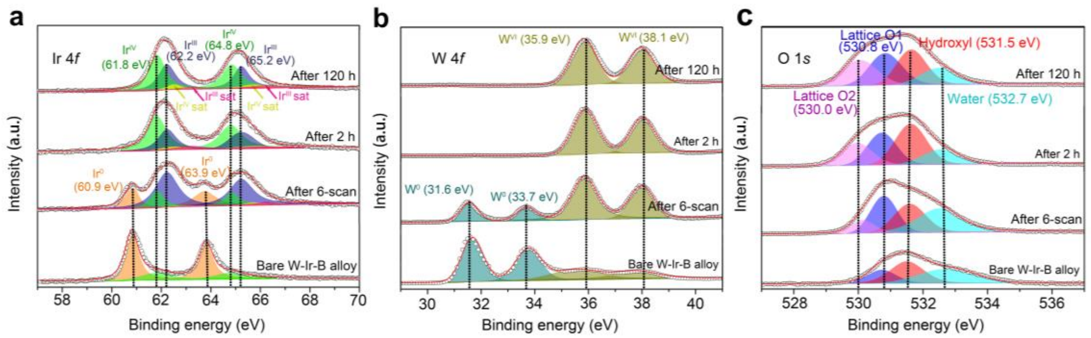

Fig. 4 XPS analyses for the surface structure during the acidic OER electrocatalysis. **a** Ir 4*f*, **b** W 4*f*, and **c** O 1*s* deconvoluted core-level peaks of the bare W-Ir-B alloy catalyst, the OER activated alloy catalyst, the alloy catalyst after 2-h OER test, and the alloy catalyst after 120-h OER test, respectively.

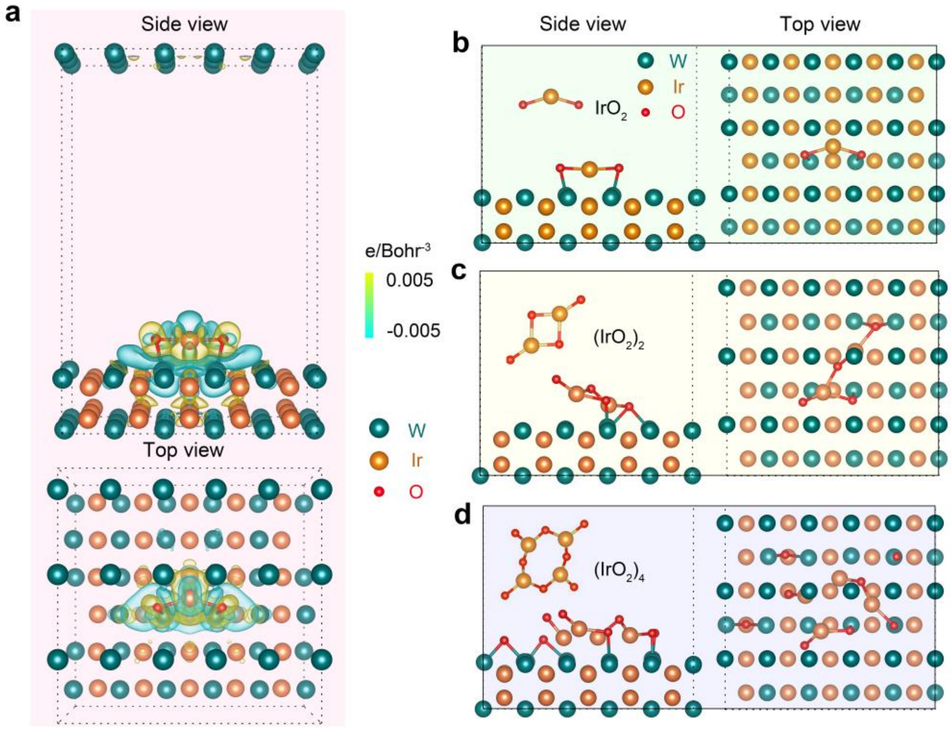

Fig. 5 Theoretical calculations for the catalyst-support interactions. a Computed differential charge density of the IrO_2 cluster adsorbed to the IrW (002) surface. Yellow and blue bubbles represent the positive and negative charges with an iso-value of 0.005 e Å^{-3}, respectively. **b-d** Optimized structures of the $(IrO_2)_n$ (n=1, 2, and 4) clusters adsorbed to the IrW (002) surface, respectively.
