# Supplementary material for: IrW nanochannel support enabling ultrastable electrocatalytic oxygen evolution at 2 A cm−2 in acidic media
Source: Nat Commun. 2021 Jun 10;12:3540. doi: 10.1038/s41467-021-23907-1 (PMC8192761; doi:10.1038/s41467-021-23907-1)
Supplement: Supplementary file 2 — Source Data [file 41467_2021_23907_MOESM2_ESM.zip › Summary (300 characters).docx]

Although electrocatalytic water splitting can generate renewable fuels, it is challenging to find water oxidation catalysts that are stable in acid at high current densities. Here, authors explore IrW as oxygen evolution electrocatalysts maintaining high current densities for hundreds of hours.
