# Supplementary material for: IrW nanochannel support enabling ultrastable electrocatalytic oxygen evolution at 2 A cm−2 in acidic media
Source: Nat Commun. 2021 Jun 10;12:3540. doi: 10.1038/s41467-021-23907-1 (PMC8192761; doi:10.1038/s41467-021-23907-1)
Supplement: Supplementary file 2 — Source Data [file 41467_2021_23907_MOESM2_ESM.zip › Supplementary Information.docx]

**Supplementary Information**

IrW nanochannel support enabling ultrastable electrocatalytic oxygen evolution at 2 A∙cm^-2^ in acidic media

Rui Li^1,4,6^, Haiyun Wang^2,6^, Fei Hu^3,6^*, K.C. Chan^4^*, Xiongjun Liu^5^, Zhaoping Lu^5^, Jing Wang^5^, Zhibin Li^5^, Longjiao Zeng^3^, Yuanyuan Li^3^, Xiaojun Wu^2^* & Yujie Xiong^2^*

^1^Northwestern Polytechnical University, Xi'an 710072, P. R. China

^2^Hefei National Laboratory for Physical Sciences at the Microscale iChEM (Collaborative Innovation Center of Chemistry for Energy Materials), and School of Chemistry and Materials Science, University of Science and Technology of China, Hefei 230026, P. R. China

^3^School of Materials Science and Energy Engineering, and Guangdong Key Laboratory for Hydrogen Energy Technologies, Foshan University, Foshan 528000, P. R. China

^4^Advanced Manufacturing Technology Research Centre, Department of Industrial and Systems Engineering, The Hong Kong Polytechnic University, Kowloon 999077, Hong Kong, China

^5^Beijing Advanced Innovation Center for Materials Genome Engineering, State Key Laboratory for Advanced Metals and Materials, University of Science and Technology Beijing, Beijing 100083, China

^6^These authors contributed equally: Rui Li, Haiyun Wang, and Fei Hu.;

*Corresponding author: E-mail: [mfhufei@fosu.edu.cn](mailto:mfhufei@fosu.edu.cn) (F.H.); [kc.chan@polyu.edu.hk](mailto:kc.chan@polyu.edu.hk) (K.C.); [xjwu@ustc.edu.cn](mailto:xjwu@ustc.edu.cn) (X.W.); [yjxiong@ustc.edu.cn](mailto:yjxiong@ustc.edu.cn) (Y.X.)


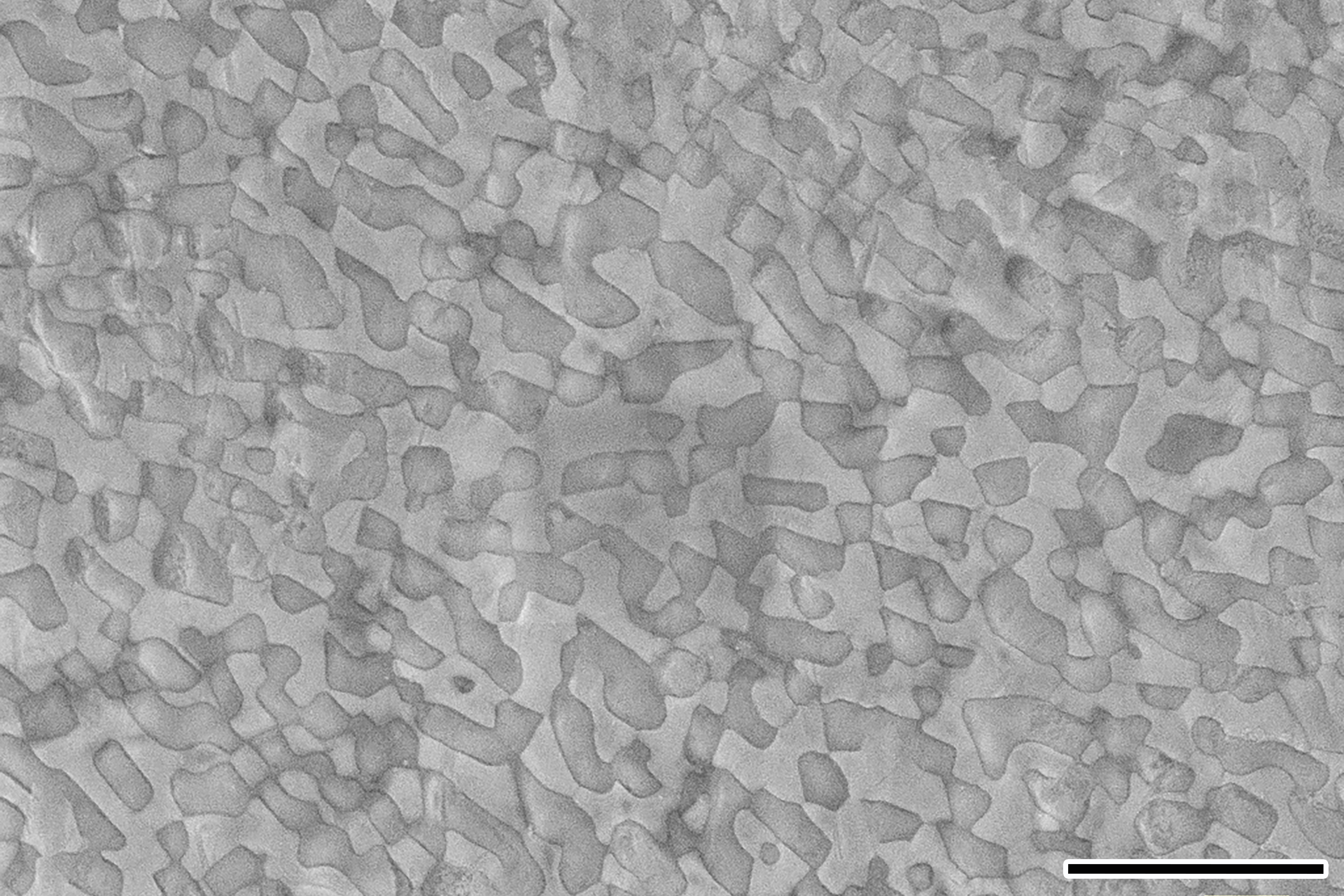


**Supplementary Fig. 1** SEM image of the W-Ir-B alloy surface with an atomic ratio of 60:20:20. Scale bar: 1 μm.


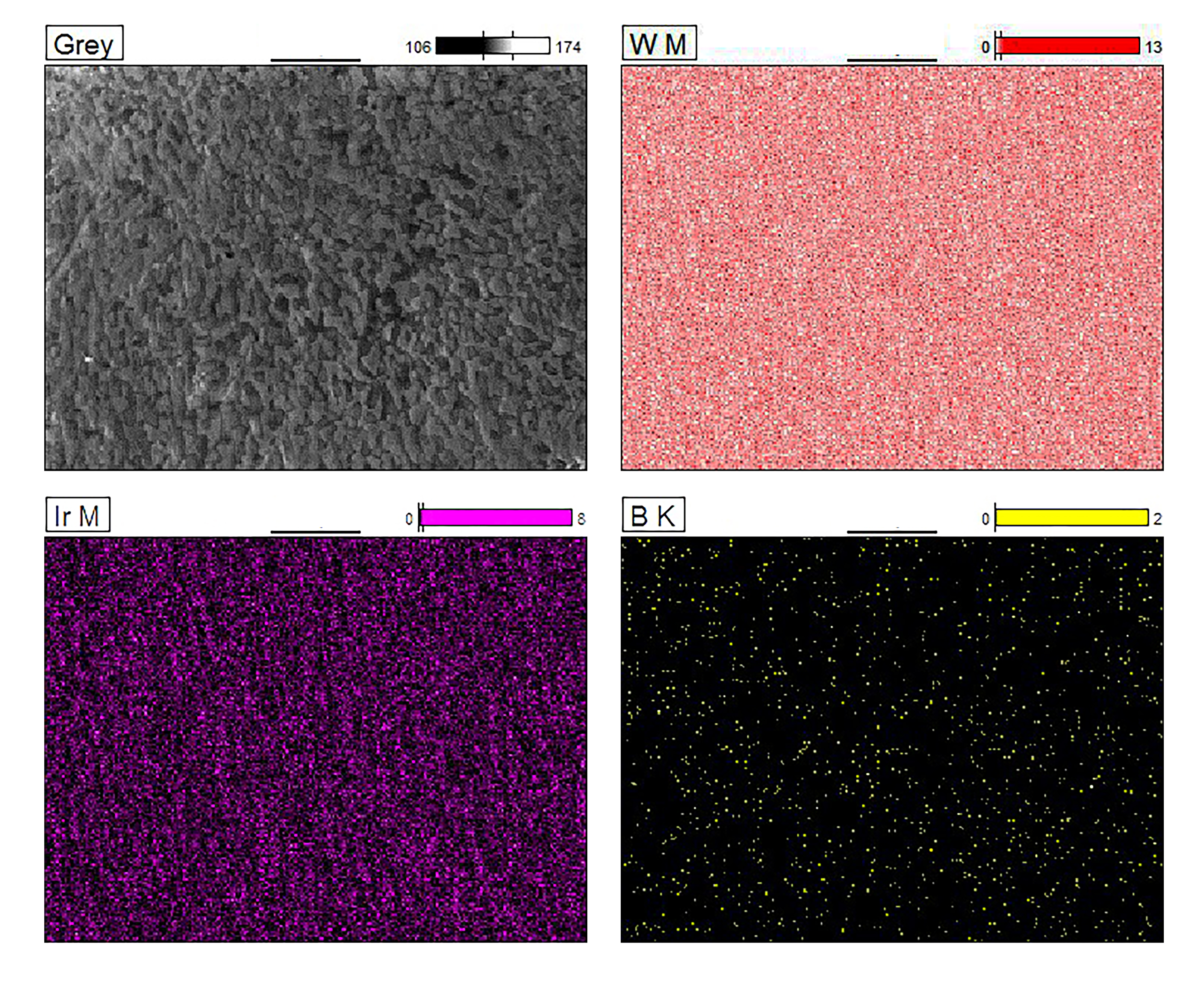


**Supplementary Fig. 2** SEM images and EDS mapping profiles of the W-Ir-B alloy surface with an atomic ratio of 60:20:20. Scale bar: 1 μm.


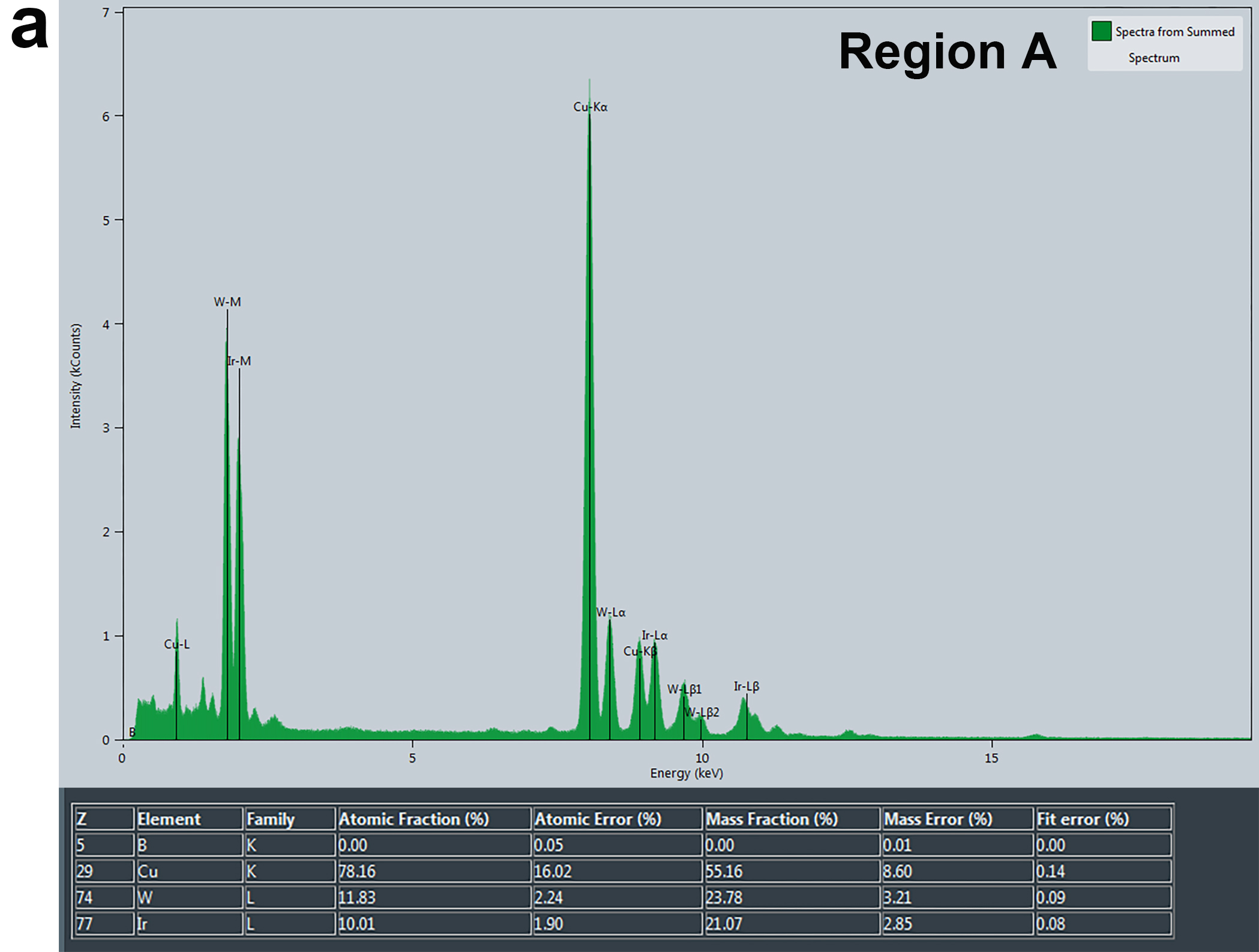


| *Element* | *Line Type* | *k Factor* | *k Factor type* | *Absorption Correction* | *Wt%* | *Wt% Sigma* | *Atomic %* |
| --- | --- | --- | --- | --- | --- | --- | --- |
| *B* | K series | 7.492 |  | 1.00 | 0.00 | 0.00 | 0.00 |
| *W* | L series | 2.186 |  | 1.00 | 50.32 | 0.59 | 51.43 |
| *Ir* | L series | 2.240 |  | 1.00 | 49.68 | 0.59 | 48.57 |
| *Total:* |  |  |  |  | 100.00 |  | 100.00 |


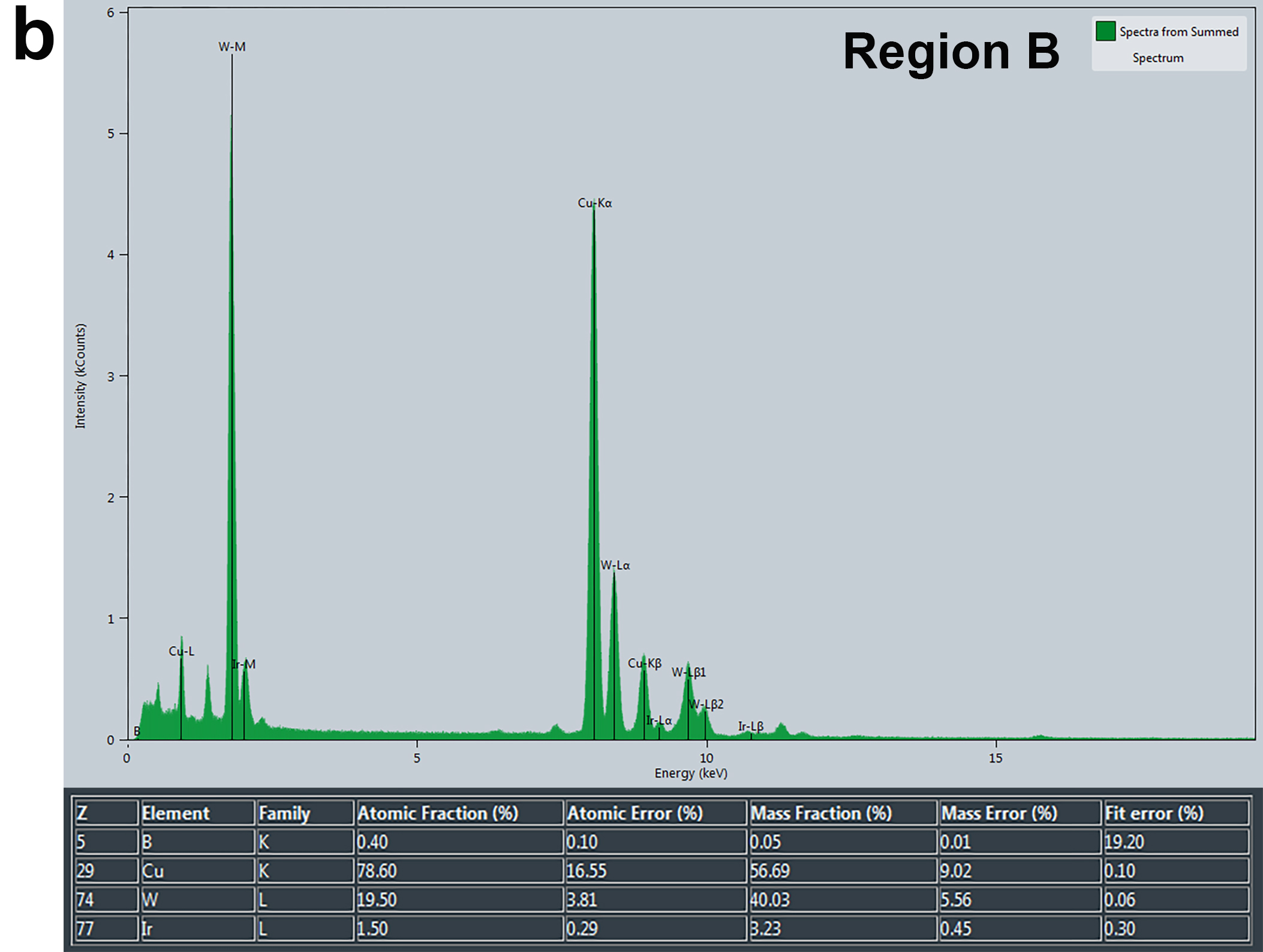


| *Element* | *Line Type* | *k Factor* | *k Factor type* | *Absorption Correction* | *Wt%* | *Wt% Sigma* | *Atomic %* |
| --- | --- | --- | --- | --- | --- | --- | --- |
| *B* | K series | 7.492 |  | 1.00 | 2.38 | 1.01 | 29.32 |
| *W* | L series | 2.186 |  | 1.00 | 94.72 | 1.17 | 68.67 |
| *Ir* | L series | 2.240 |  | 1.00 | 2.90 | 0.69 | 2.01 |
| *Total:* |  |  |  |  | 100.00 |  | 100.00 |

**Supplementary Fig. 3** TEM EDS analysis of (**a**) region A and (**b**) region B in Fig. 1b.


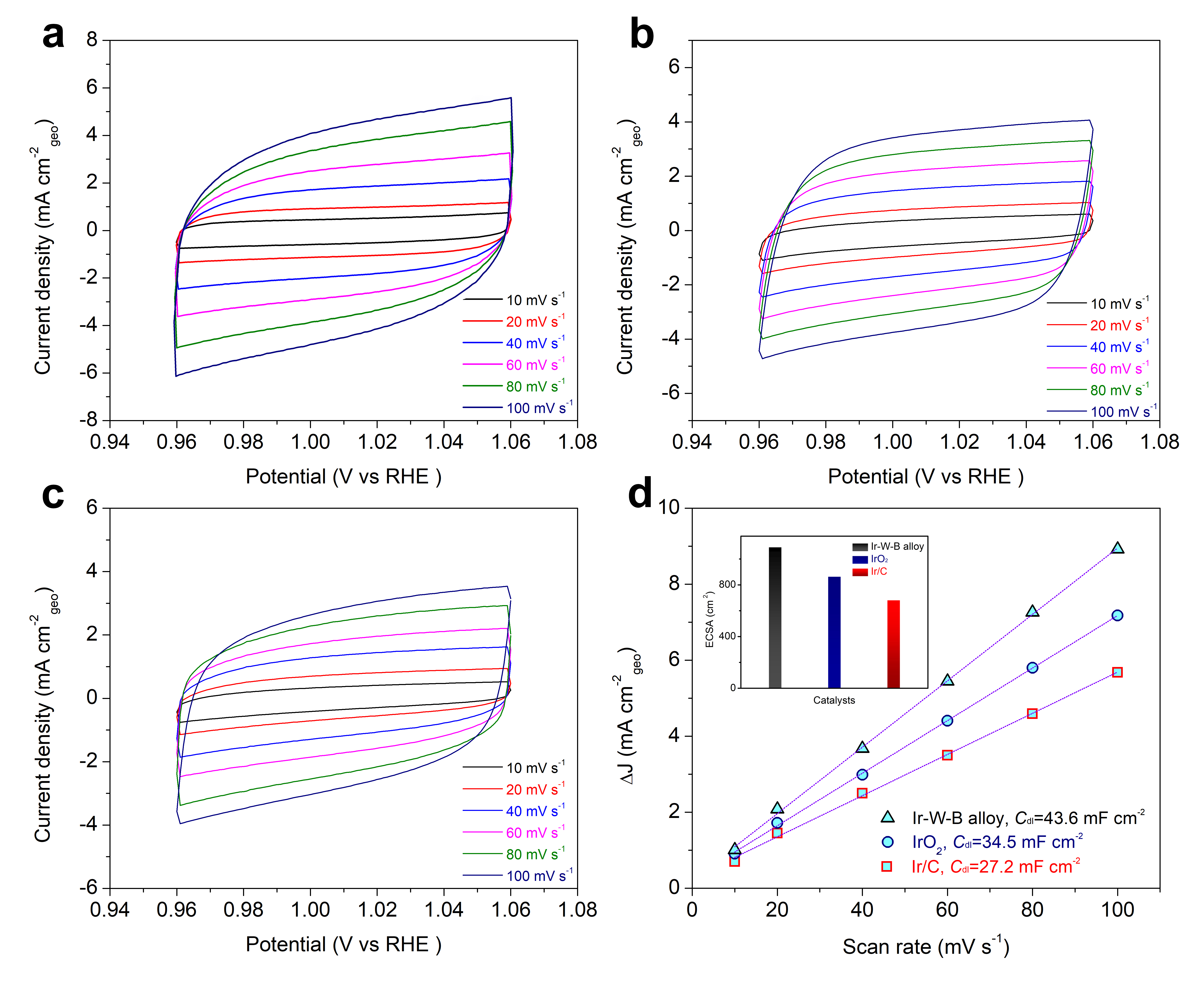


**Supplementary Fig. 4** Cyclic voltammograms (CVs) of the (**a**) Ir-W-B alloy, (**b**) IrO_2_, and (**c**) Ir/C catalysts, respectively. The CV curves were performed at different scan rates (10, 20, 40, 60, 80, and 100 mV s^-1^) from 0.96 to 1.06 V vs RHE. (**d**) The plots of current densities against scan rates. Δj is the difference between anodic and cathodic current density at a potential of 1.01 V vs RHE. The slope of the fitting line is twice of the electrochemical double-layer capacitance, which is proportional to the ECSA of the catalysts. The ECSA value (inset in Fig. 4d) was calculated based on the equation of ECSA=*C*_dl_/*C*_s_, where *C*_dl_ is the electrochemical double-layer capacitance and C_s_ is the specific capacitance. The *C*_S_ for a flat surface was generally found to be in the range of 20 to 60 μF cm^-2^. In this work, we assumed a value of 40 μF cm^-2^.

***
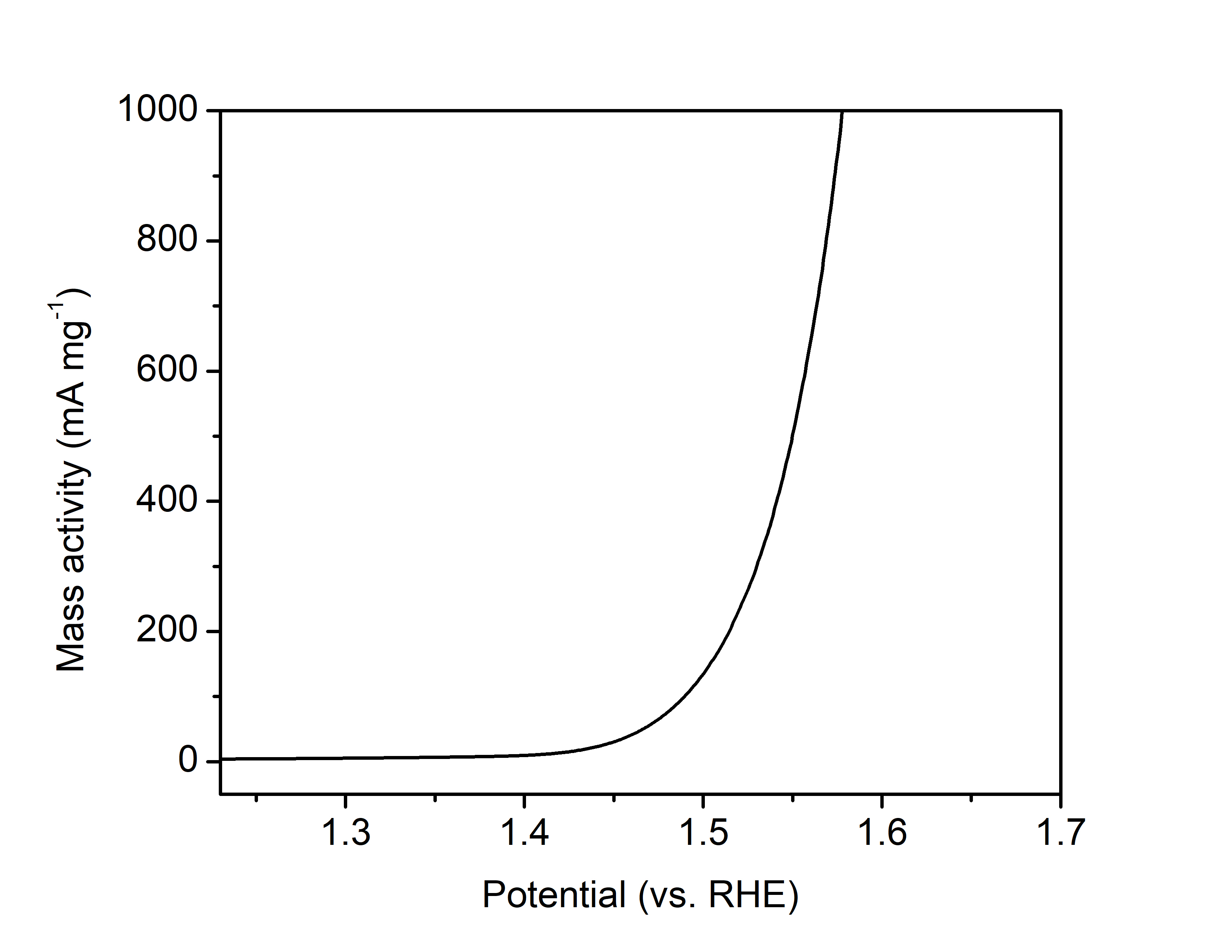
***

**Supplementary Fig. 5** The Ir mass normalized current density vs. potential of the Ir-W-B alloy catalyst.


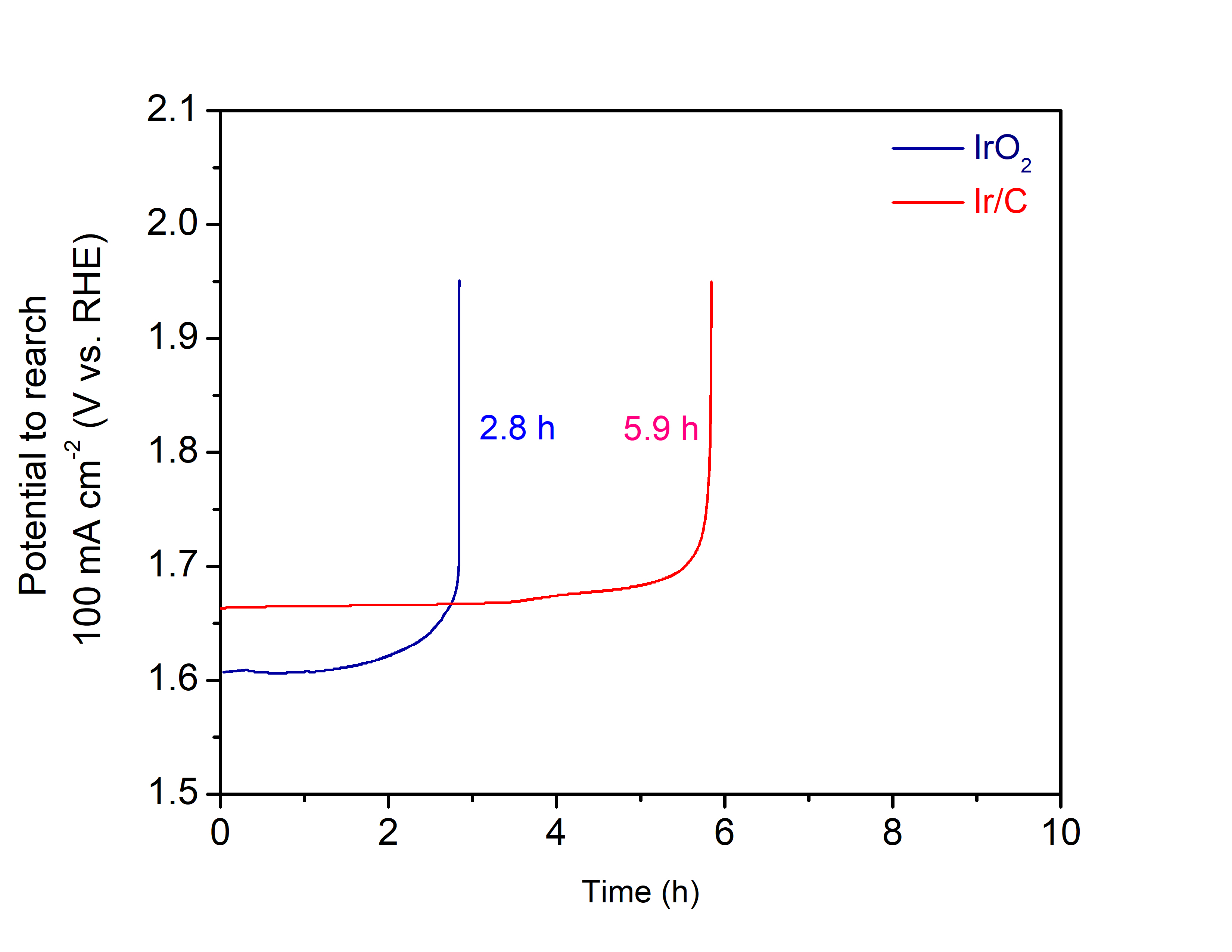


**Supplementary Fig. 6** Chronopotentiometry curves of commercial IrO_2_ and Ir/C catalysts in 0.5 M H_2_SO_4_ electrolyte at a current density of 100 mA cm^-2^_geo_. The efficacy loss is mainly due to the catalyst detachment resulting from the dissolution of the carbon support and the drastic attack of the continuous O2 bubbles.

**
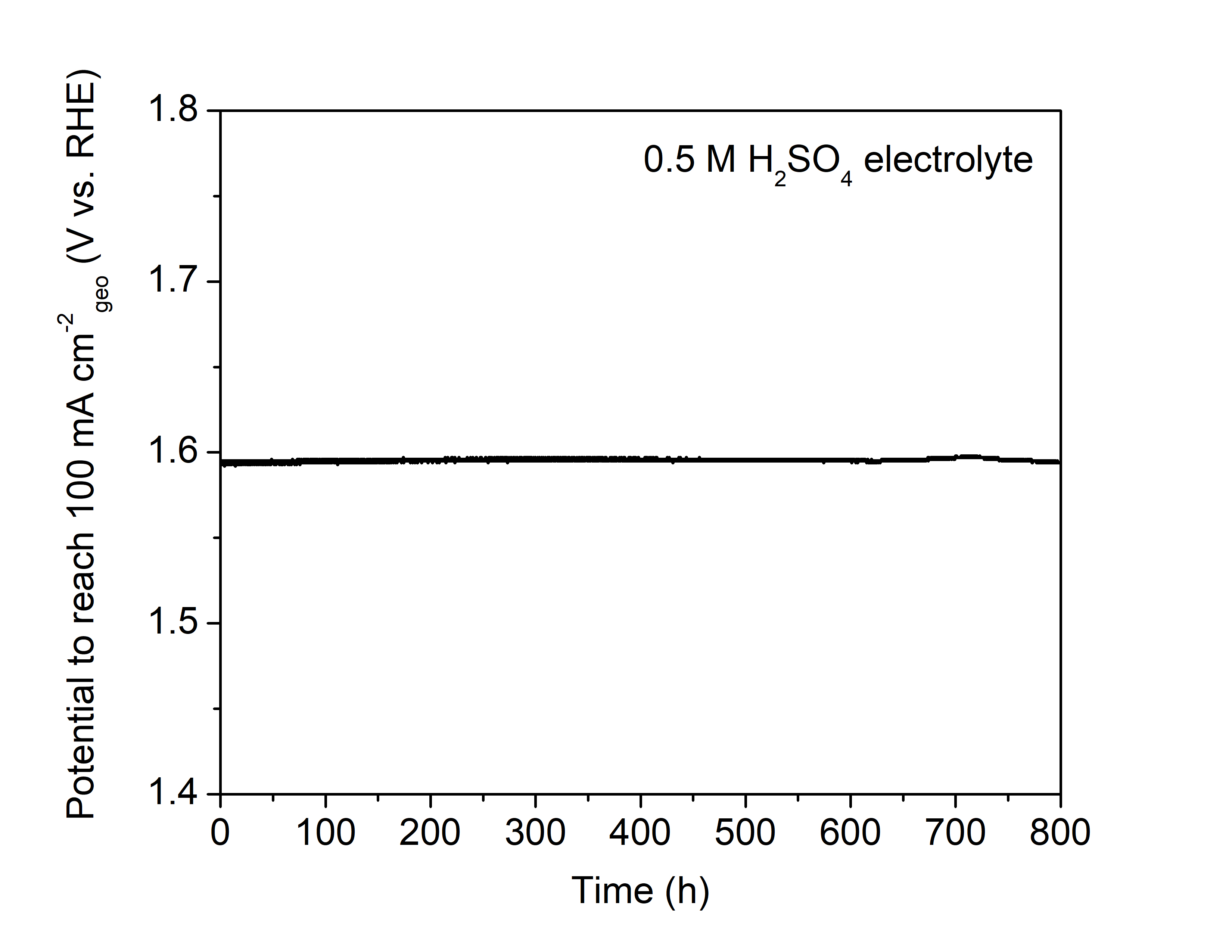
**

**Supplementary Fig. 7** Durability test of the W-Ir-B alloy catalyst in 0.5 M H_2_SO_4_ electrolyte at a current density of 100 mA cm^-2^_geo_.


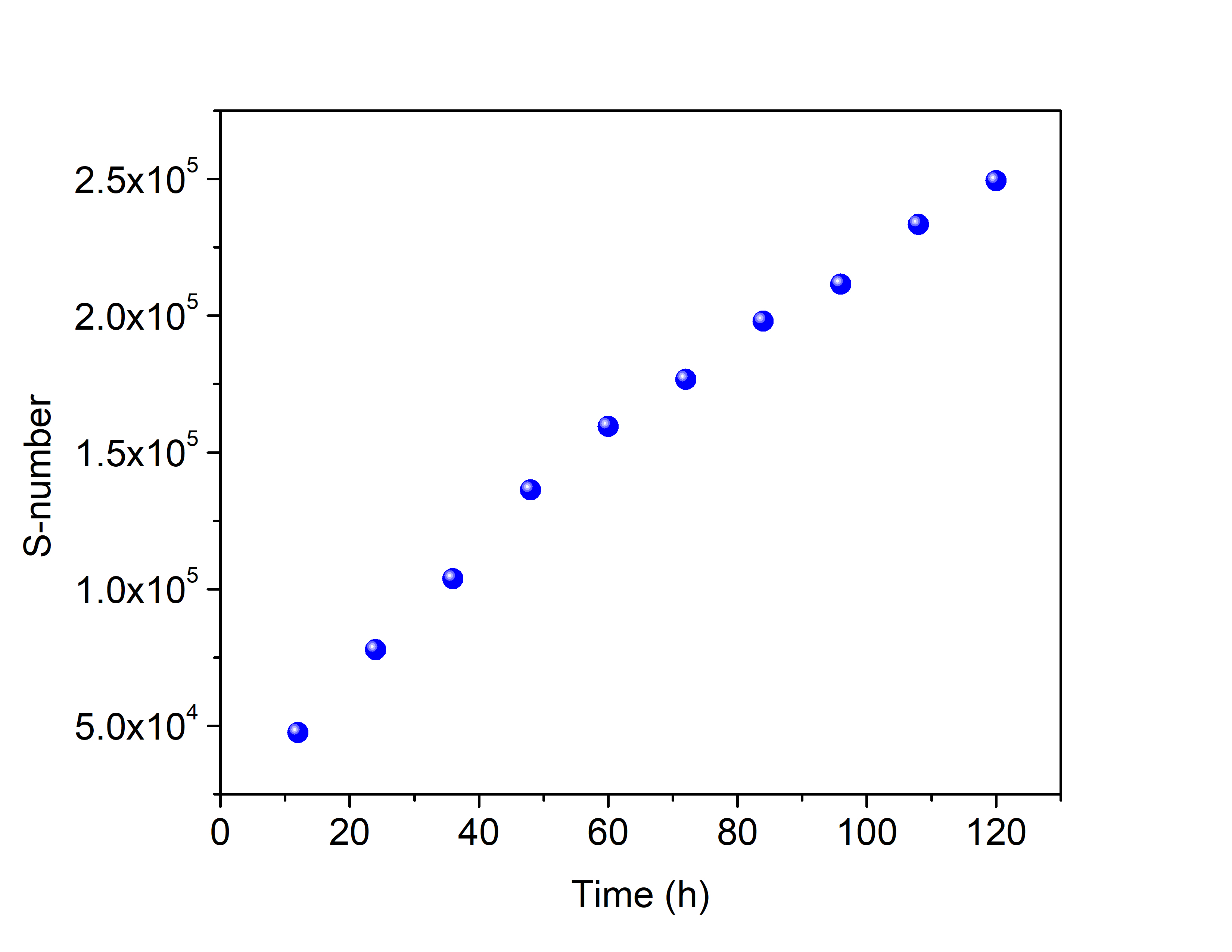


**Supplementary Fig. 8** Calculated S-number during the 120-h acidic OER under the current density of 100 mA cm^-2^_geo_.


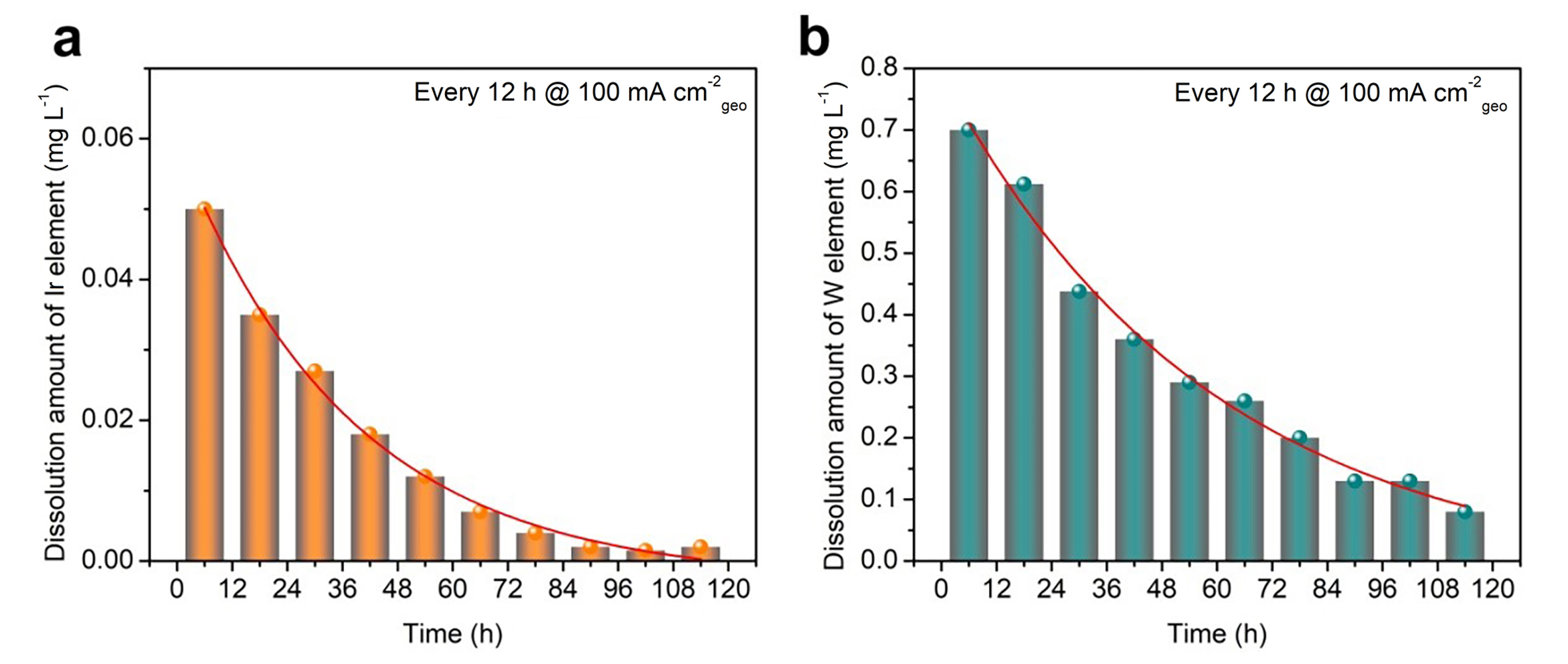


**Supplementary Fig. 9** Dissolution amount variation of the (**a**) Ir and (**b**) W elements for every 12 h during the 120-h acidic OER test.


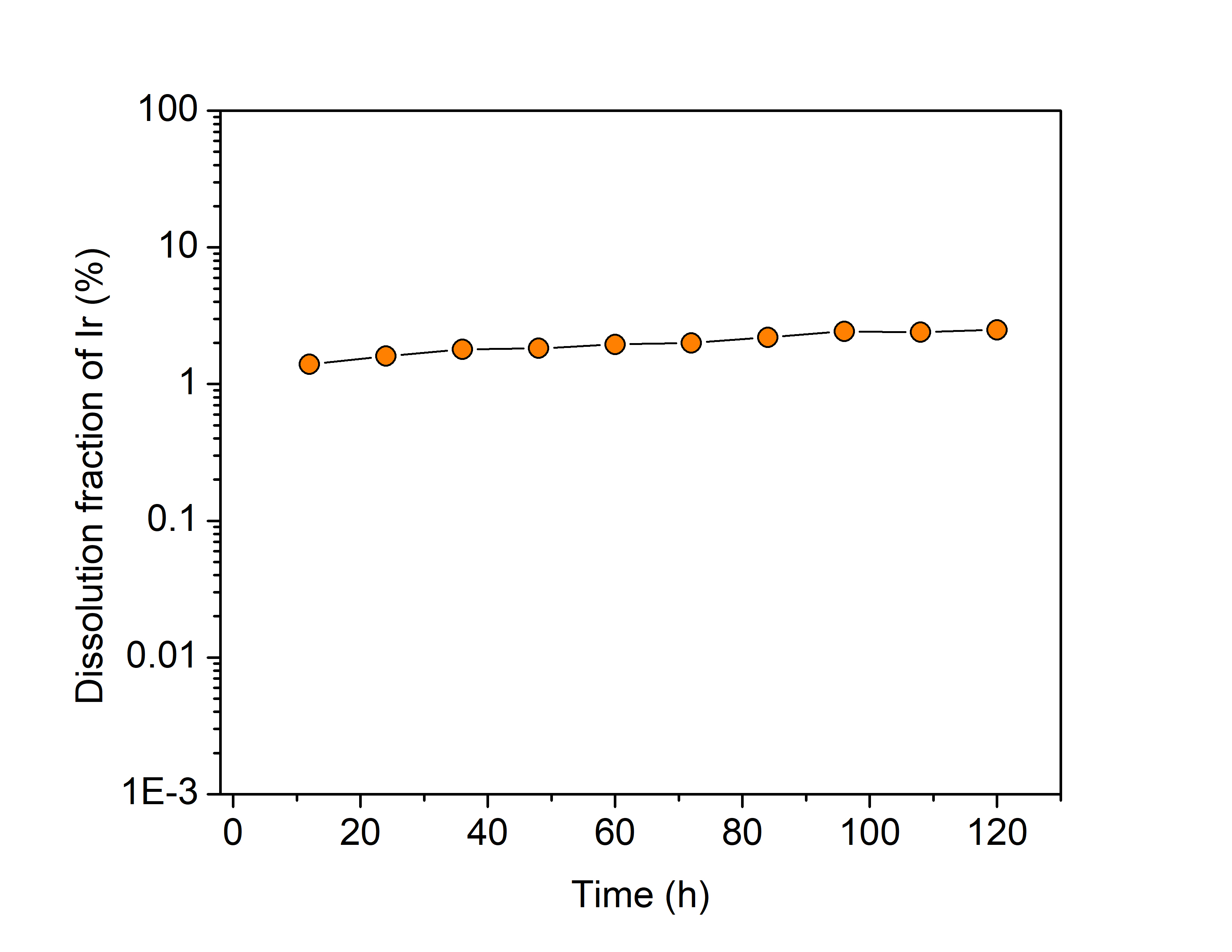


**Supplementary Fig. 10** Dissolution of Ir element in comparison with the initial surface Ir mass during the 120-h acidic OER test at 100 mA cm^-2^_geo_.


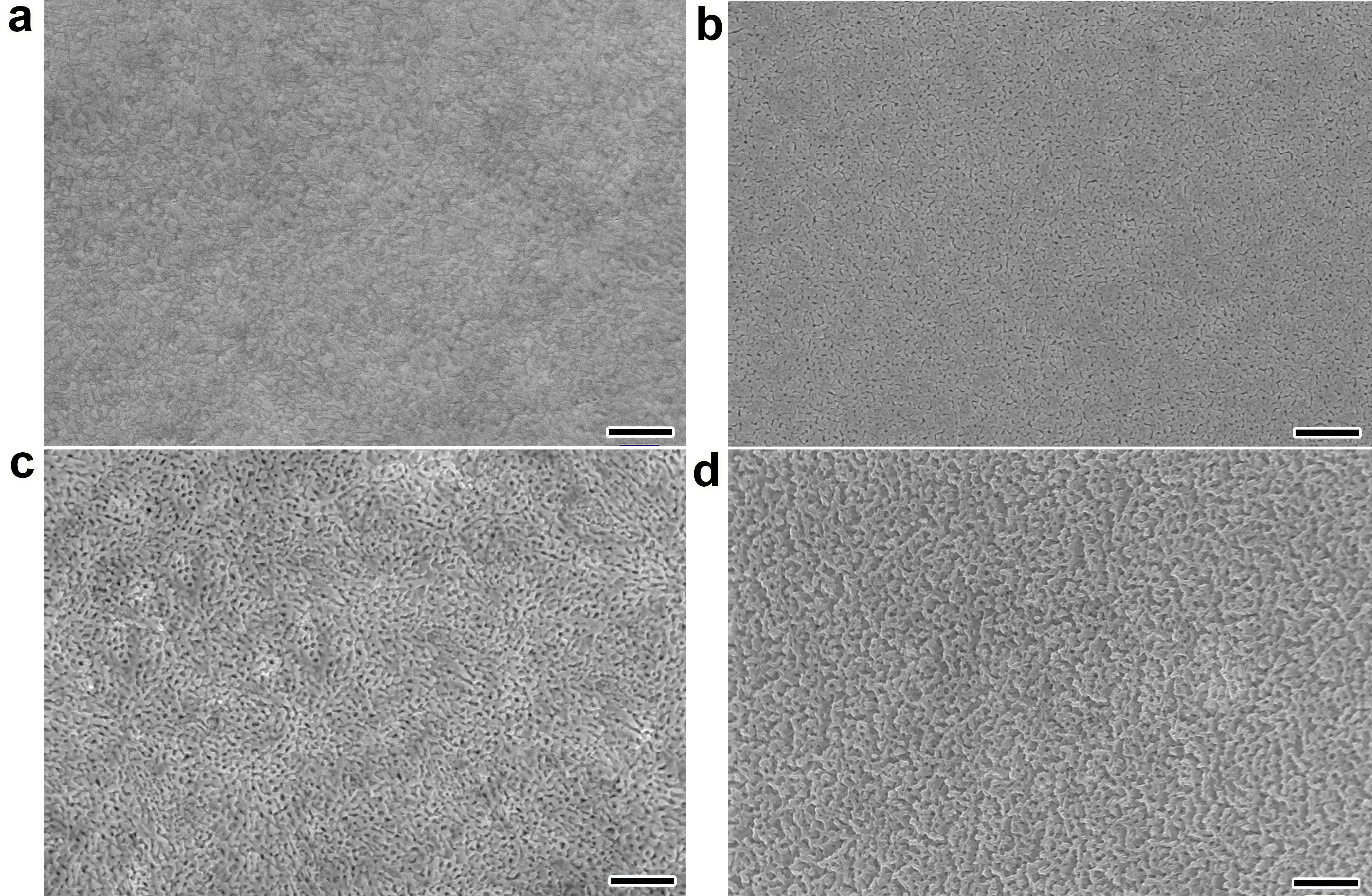


**Supplementary Fig. 11** Surface structure evolution of the W-Ir-B alloy catalyst during the acidic OER: (**a**) 0 min; (**b**) 5 min; (**c**) 10 min; (**d**) 30 min. Scale bar: 1 μm.


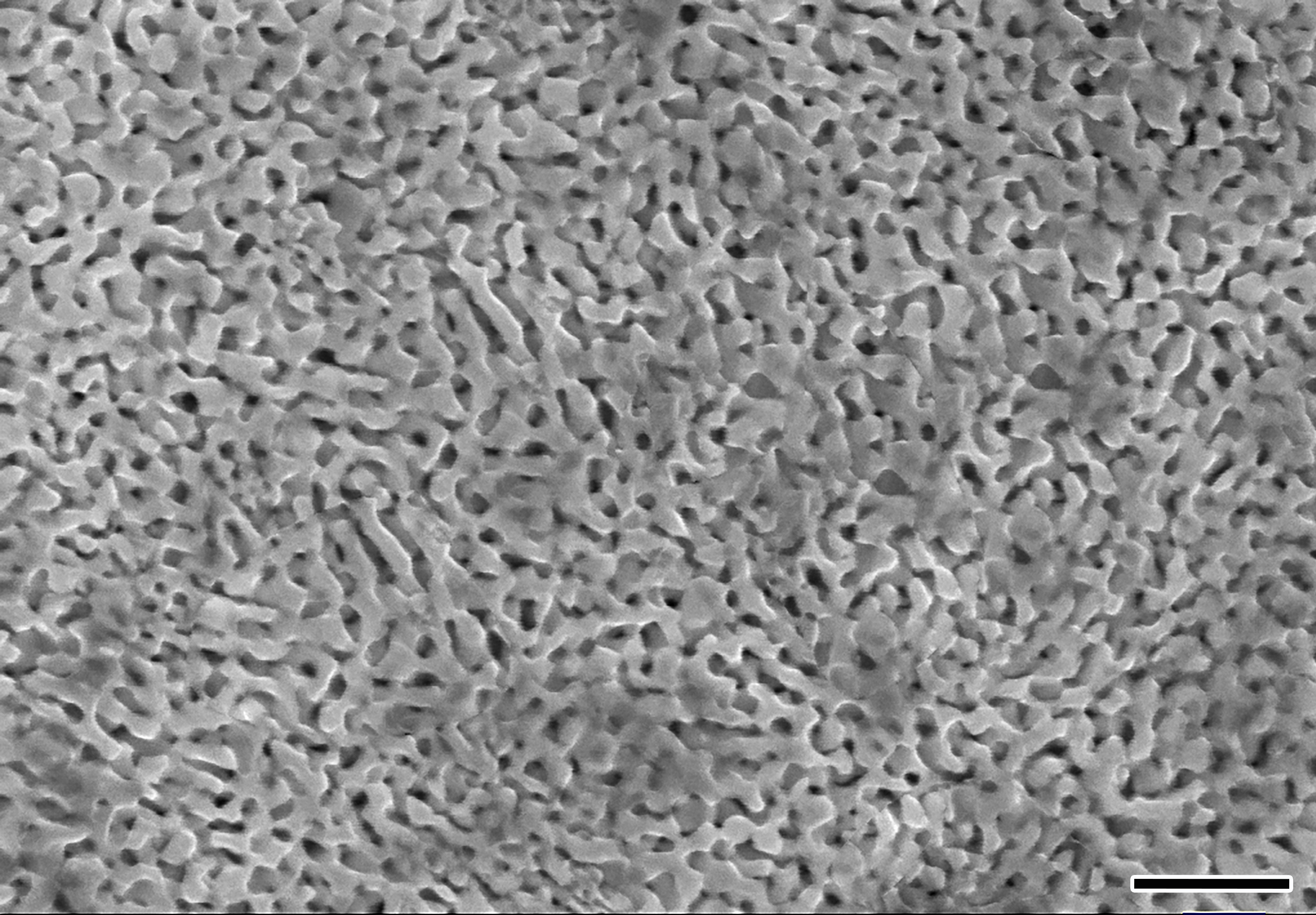


**Supplementary Fig. 12** Top-view SEM images of the W-Ir-B alloy catalyst after the 2-h OER test. Scale bar: 500 nm.

**
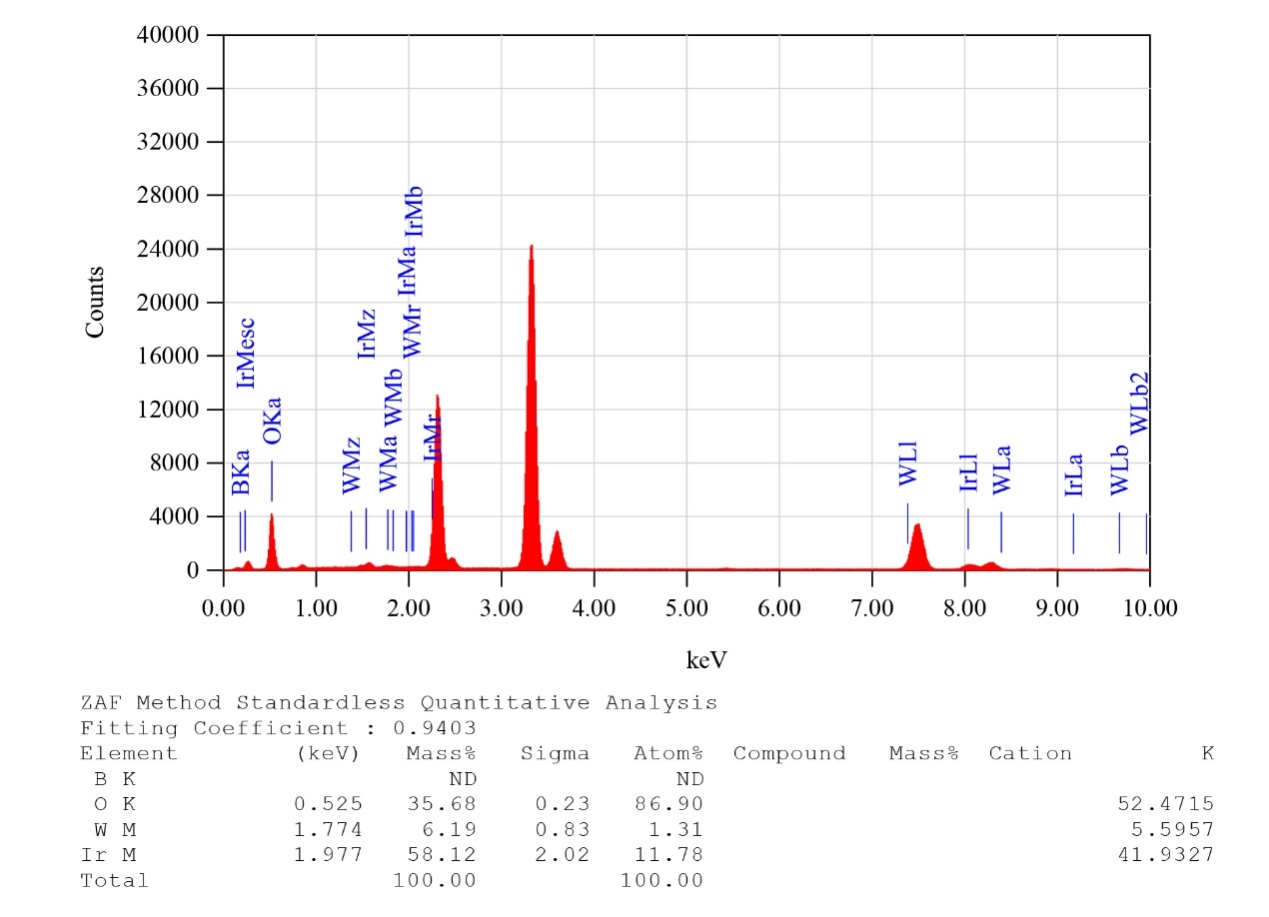
**

**Supplementary Fig. 13** TEM EDS analysis for the surface active oxides.

**
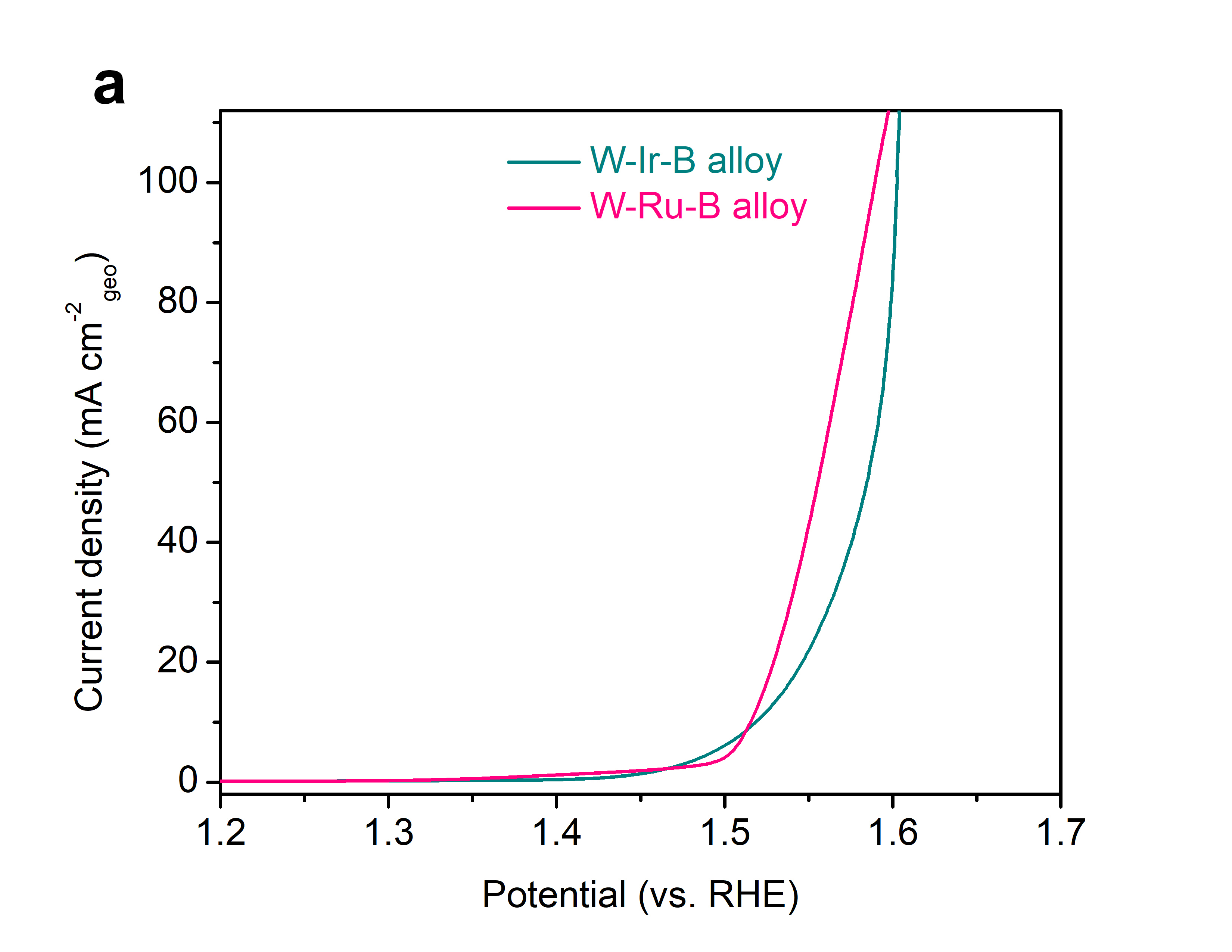
**

**
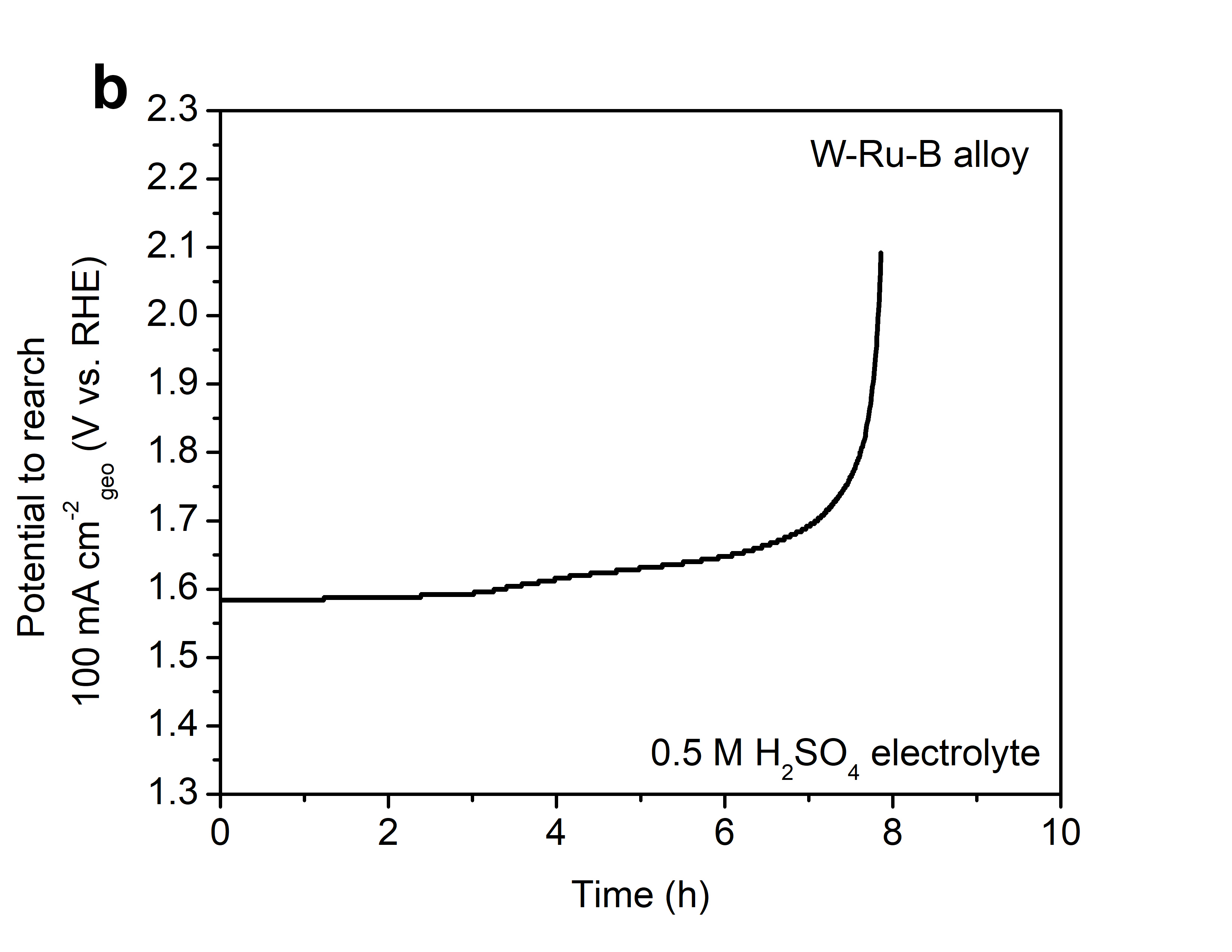
**

**Supplementary Fig. 14** (**a**) OER polarization curves of the W-Ir-B and W-Ru-B alloy catalysts in 0.5 M H_2_SO_4_ electrolyte. (**b**) Chronopotentiometry curves of the W-Ru-B alloy catalyst at a current density of 100 mA cm^-2^_geo_.

**
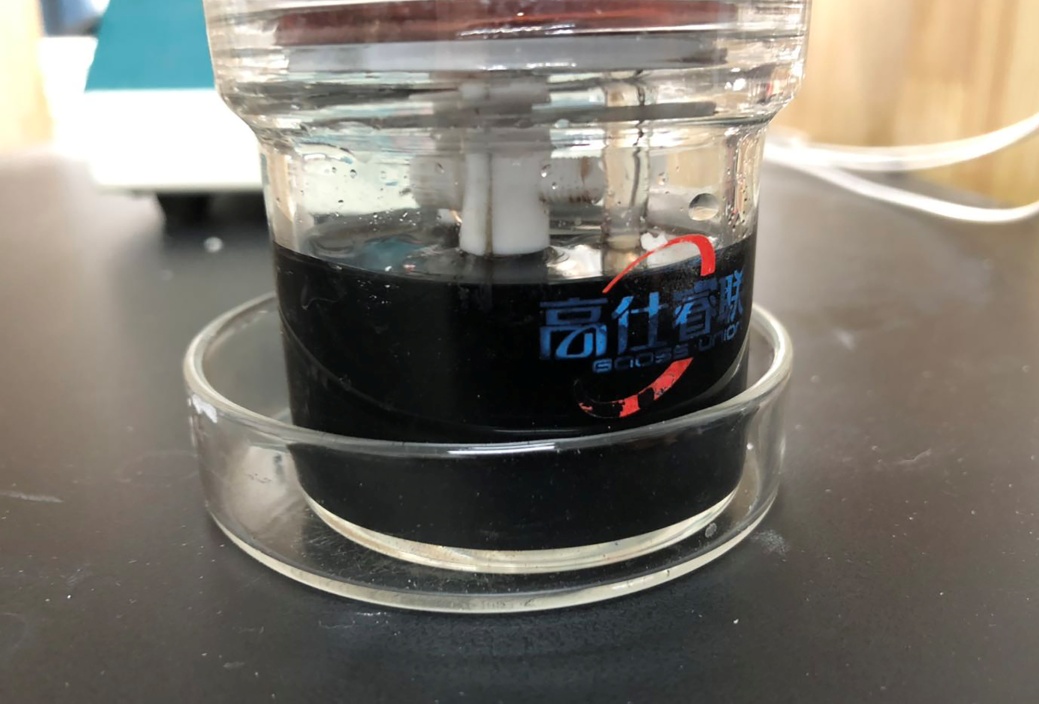
**

**Supplementary Fig. 15** Electrolyte color of the W-Ru-B alloy catalyst after the 6-h OER durability test at the current density of 100 mA cm^-2^_geo_.

**
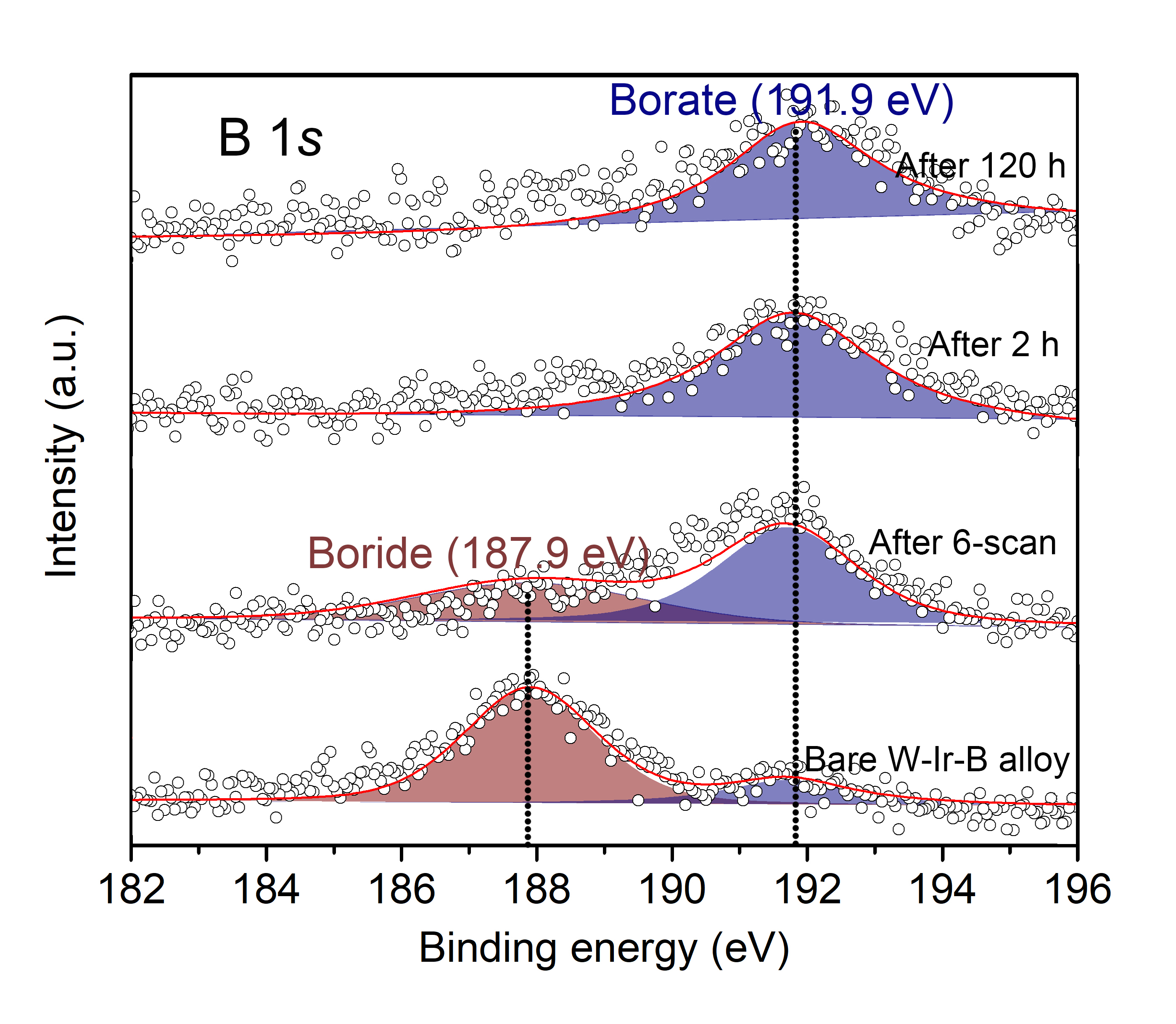
**

**Supplementary Fig. 16** XPS analyses for B 1*s* of the bare W-Ir-B alloy catalyst, the OER activated alloy catalyst, the alloy catalyst after 2-h OER test, and the alloy catalyst after 120-h OER test.


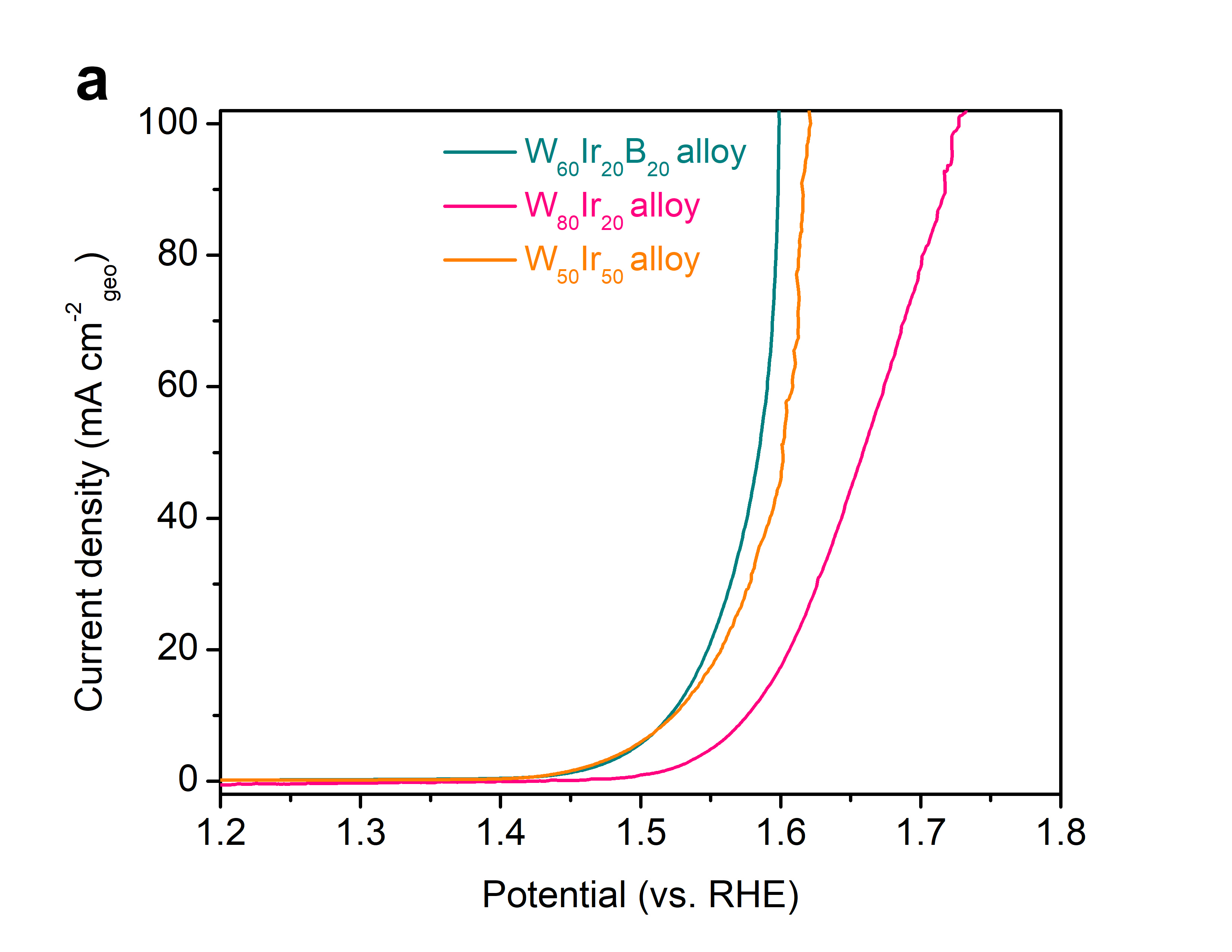

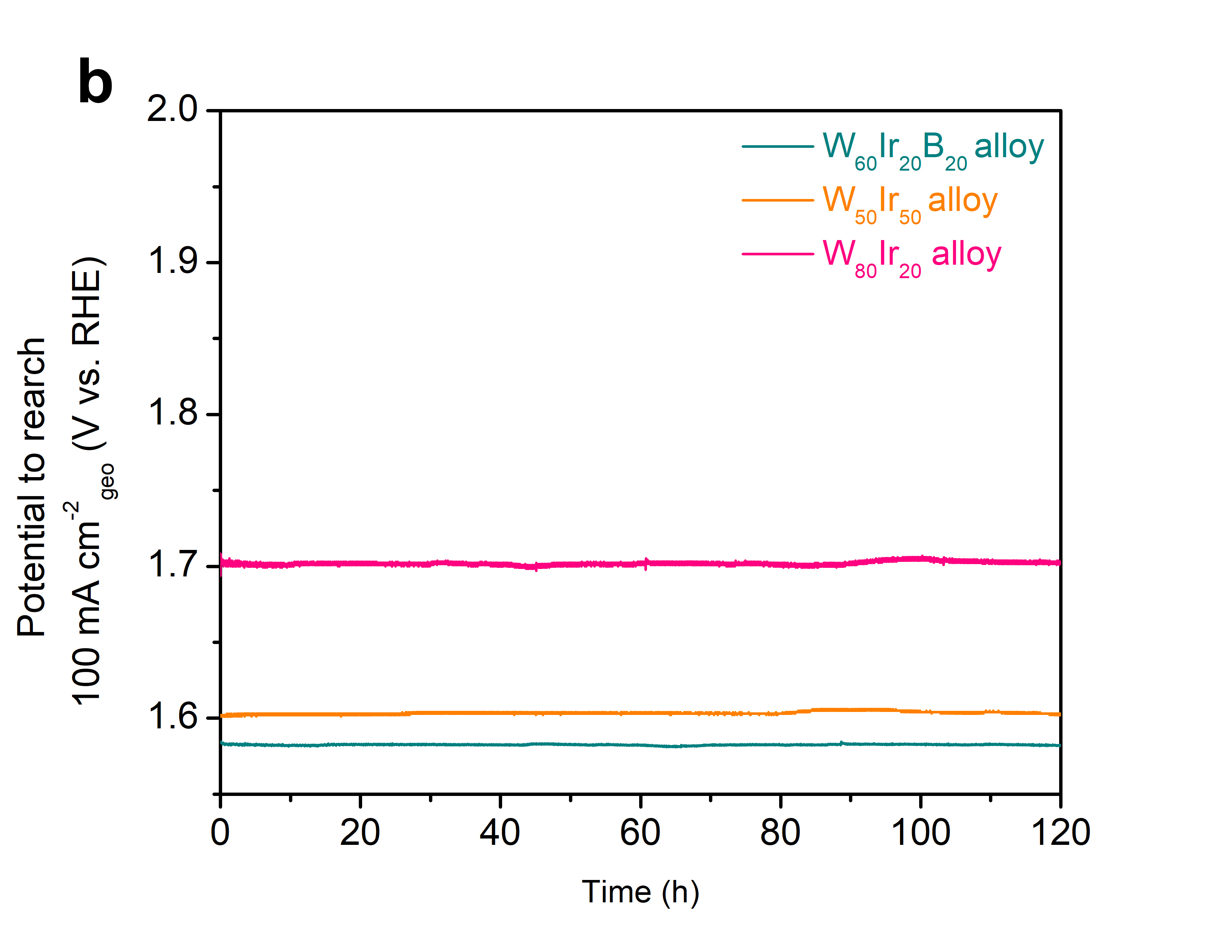


**Supplementary Fig. 17** (**a**) OER polarization curves of the W_60_Ir_20_B_20_, W_80_Ir_20,_ and W_50_Ir_50_ alloy catalysts in 0.5 M H_2_SO_4_ electrolyte. (**b**) 120-h OER durability tests of the three alloy catalysts at a current density of 100 mA cm^-2^_geo_.


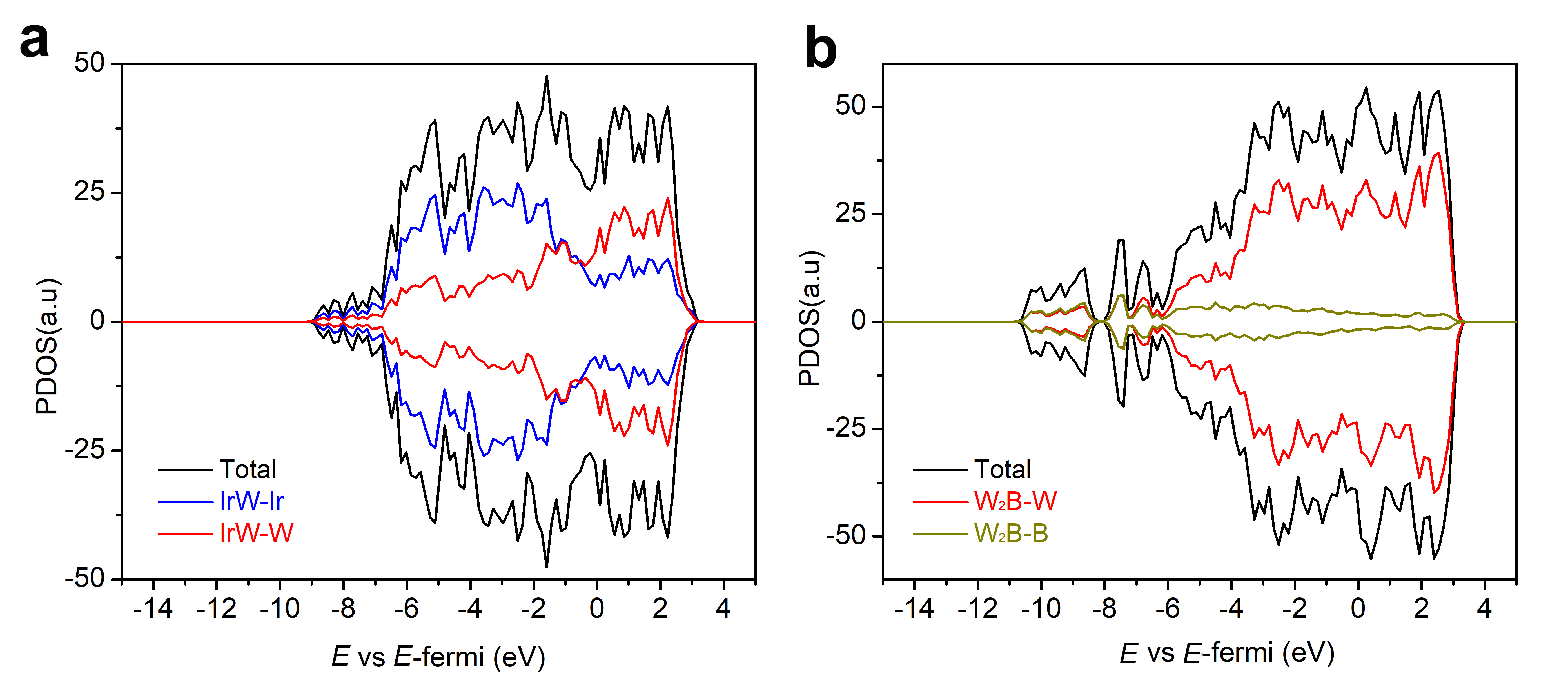


**Supplementary Fig. 18** Computed partial density of states (PDOS) of (**a**) the IrW (002) surface and (**b**) the W_2_B (211) surface.


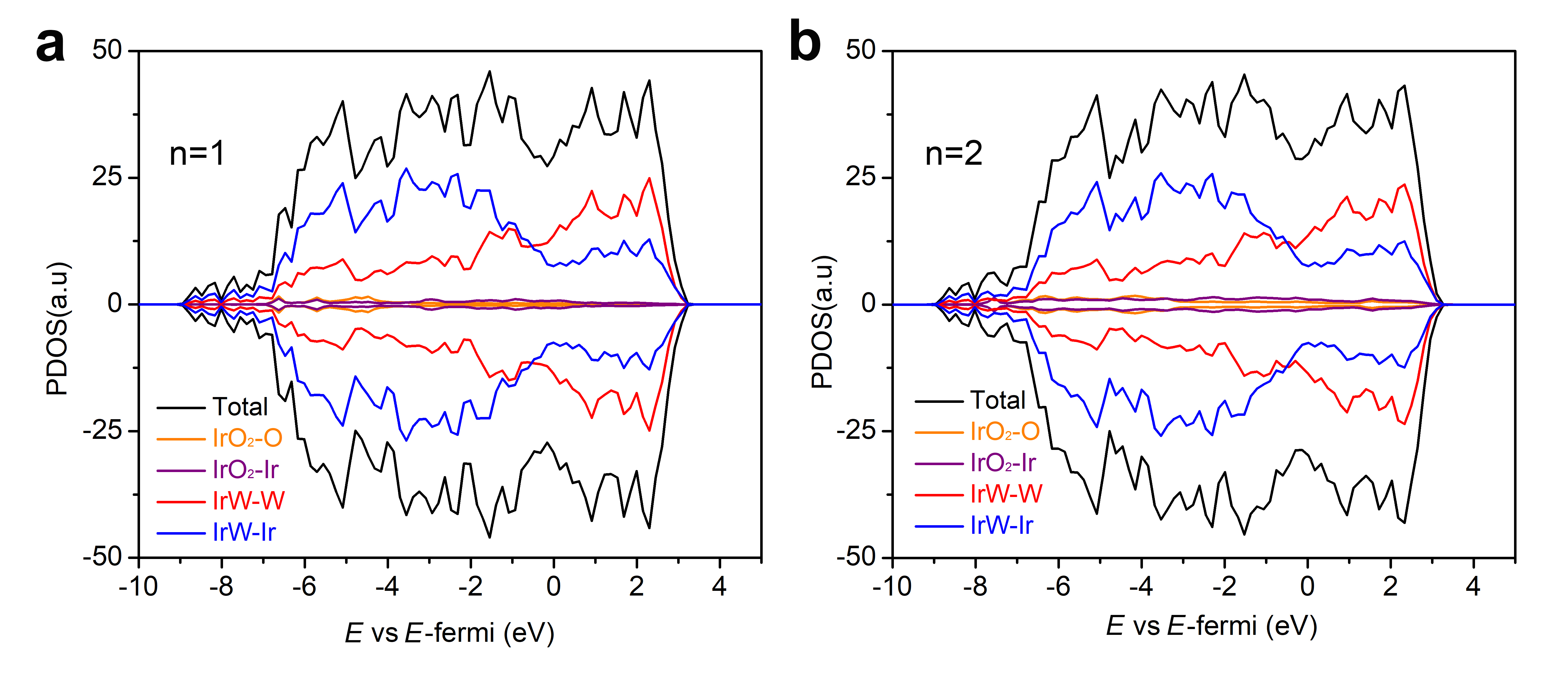


**c**


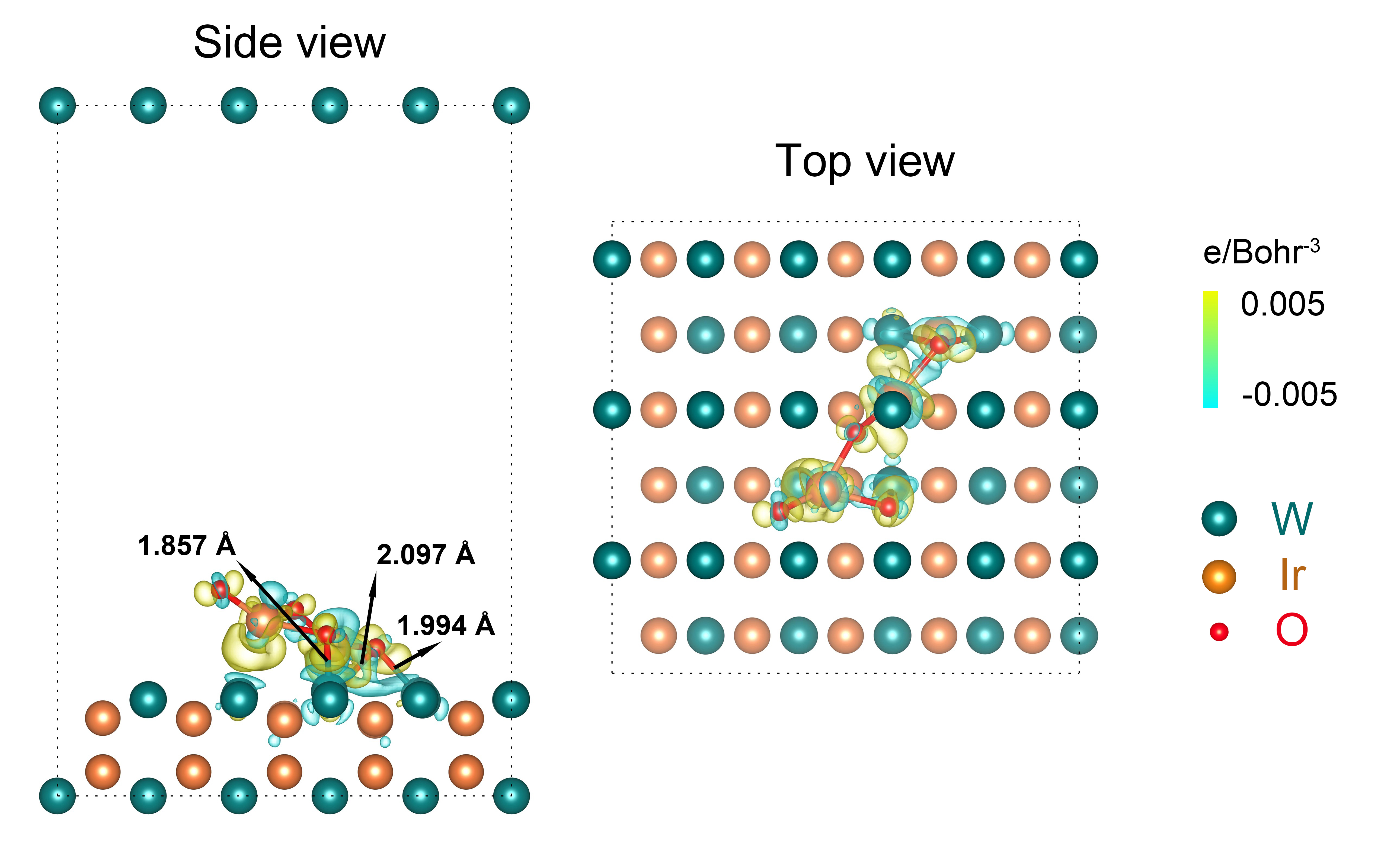


**Supplementary Fig. 19** Computed PDOS of The (IrO_2_)_n_ clusters adsorbed on the IrW (002) surface: (**a**) n=1; (**b**) n=2. (**c**) Computed differential charge densities of the (IrO_2_)_2_ clusters adsorbed to the IrW (002) surface. Yellow and blue bubbles represent the positive and negative charge with an iso-value of 0.005 e Å^-3^, respectively.

The calculation formula of the differential charge density is:

$$\Delta\rho= \rho_{\mathrm{AB}} - \rho_{A}- \rho_{B}$$

$\rho_{A}$ represents the charge density of the optimized structure after the IrO_2_ adsorption on the IrW (002) surface, $\rho_{A}$ and $\rho_{B}$ represent the charge density of the IrW (002) surface and the IrO_2_ clusters, respectively. By subtracting them, we can get the charge density change after the IrO_2_ adsorption on the IrW (002) surface.

**Supplementary Table 1** Comparison of overpotential and mass activity of the Ir-W-B alloy catalyst with previously reported Ir-based catalysts in acidic media.

| Materials | Ir loading  (μg cm^-2^) | Overpotential@ 10 mA cm^-2^_geo_  (mV) | J_m_@1.53 V vs. RHE  (mA mg_Ir_ ^-1^) | Ref. |
| --- | --- | --- | --- | --- |
| Ir-W-B alloy | 78.9 | 291 | 518 | This work |
| IrNiO_x_ | 10.2 | / | 676 | ^1^ |
| IrO_x_ | 10.2 | / | 325 |  |
| La_2_LiIrO_6_ | 250 (catalyst) | / | <100 | ^2^ |
| SrIrO_3_ | / | ~290 | / | ^3^ |
| Ba_2_NdIrO_6_ | 15 (catalyst) | 380 | / | ^4^ |
| IrO_x_-network | 10 | ~330 | 817±119 | ^5^ |
| Li-IrO_x_ | 50 (catalyst) | 300 | 100 | ^6^ |
| Pt-Ir-Pd nanocage | 16.8 | 372 | 200 | ^7^ |
| IrO_2_ nanoneedles | 250 | 313 | ~55 | ^8^ |
| IrNiCu nanoframe | 20 | 300 | 460 | ^9^ |
| 6H-SrIrO_3_ | 52.7 | 248 | 75 | ^10^ |

**Supplementary Table 2** Comparison of the OER performance and stability for the recently reported electrocatalysts in acidic media.

| **Materials** | **substrate** | **Electrolyte** | **Overpotential at 10 mA cm^-2^_geo_ (mV)** | **Tafel slope**  **(mV dec^-1^)** | **Test condition** | **Stability (h)** | **Ref.** |
| --- | --- | --- | --- | --- | --- | --- | --- |
| W_60_Ir_20_B_20_ | Self-supported | 0.5 M H_2_SO_4_ | 291 | 78 | 100  mA cm^-2^_geo_ | >800 | This work |
| IrO_x_/SrIrO_3_ | SrTiO_3_ | 0.5 M H_2_SO_4_ | 270-290 | / | 10 mA cm^-2^_geo_ | 30 | ^3^ |
| Ir  nanosheet | Glassy carbon | 0.5 M H_2_SO_4_ | 240 | 49 | 10 mA cm^-2^_geo_ | >8 | ^11^ |
| Co_3_O_4_@C | Carbon paper | 0.5 M H_2_SO_4_ | 370 | 82 | 100 mA cm^-2^ | 86.8 | ^12^ |
| IrOOH Nanosheet | Ti platelet | 0.1 M HClO_4_ | 344 | 58 | 10 mA cm^-2^_geo_ | <6 | ^13^ |
| Nanoporous Ir_70_Ni_15_Co_15_ | Self-supported | 0.1 M HClO_4_ | 220 | 44.1 | 10 mA cm^-2^_geo_ | 10 | ^14^ |
| Ir_73.9_W_26.1_ ND | Glassy carbon | 0.1 M HClO_4_ | 300 | 56.6 | 10 mA cm^-2^_geo_ | 8 | ^15^ |
| Ir/Co_4_N | Glassy carbon | 0.5 M H_2_SO_4_ | 310 | 62 | 10 mA cm^-2^_geo_ | 2 | ^16^ |
| IrNi nanocluster | Glassy carbon | 0.5 M H_2_SO_4_ | 280 | / | 10 mA cm^-2^_geo_ | 10 | ^17^ |
| Ir_6_Ag_9_ nanotubes | Glassy carbon | 0.5 M H_2_SO_4_ | 285 | 61.1 | 5 mA cm^-2^_geo_ | 36 | ^18^ |
| Ir single atoms | Glassy carbon | 0.5 M H_2_SO_4_ | 250 | 58.2 | 10 mA cm^-2^_geo_ | 12 | ^19^ |
| Nanoporous AlNiCoIrMo | Self-supported | 0. 5 M H_2_SO_4_ | 233 | 55.2 | 10 mA cm^-2^_geo_ | 50 | ^20^ |
| NiIr Nanocage | Glassy carbon | 0.05 M H_2_SO_4_ | 301 | 46.6 | 1 mA cm^-2^_geo_ | 10 | ^21^ |
| Ir@IrO_x_ | Glassy carbon | 0.05 M H_2_SO_4_ | 282 | 69.1 | 10 mA cm^-2^_geo_ | 24 | ^22^ |
| IrO_2_/CNT | Glassy carbon | 0.5 M H_2_SO_4_ | 293 | 67 | 10 mA cm^-2^_geo_ | 10 | ^23^ |
| IrO_x_ | carbon cloth | 0.5 M H_2_SO_4_ | 185 | 35.5 | 50 mA cm^-2^_geo_ | 50 | ^24^ |
| Ir/Graphite foam | Self-supported | 0.5 M H_2_SO_4_ | 290 | 46 | 10 mA cm^-2^_geo_ | 10 | ^25^ |
| Ir_0.5_W-900R | Glassy carbon | 0.1 M HClO_4_ | 290~300 | 42 | 10 mA cm^-2^_geo_ | 100 | ^26^ |

**Reference**

1. Nong, H.N. *et al.* A unique oxygen ligand environment facilitates water oxidation in hole-doped IrNiO_x_ core-shell electrocatalysts. *Nat. Catal.* **1**, 841-851 (2018).

2. Grimaud, A*. et al.* Activation of surface oxygen sites on an iridium-based model catalyst for the oxygen evolution reaction. *Nat. Energy* **2**, 1-10 (2016).

3. Seitz, L.C*. et al.* A highly active and stable IrO_x_/SrIrO_3_ catalyst for the oxygen evolution reaction. *Science* **353**, 1011-1014 (2016).

4. Diaz-Morales, O*. et al.* Iridium-based double perovskites for efficient water oxidation in acid media. *Nat. Commun.* **7**, 1-6 (2016).

5. Jensen, A.W*. et al.* Self-supported nanostructured iridium-based networks as highly active electrocatalysts for oxygen evolution in acidic media. *J.Mater. Chem. A* **8**, 1066-1071 (2020).

6. Gao, J*. et al.* Breaking long-range order in iridium oxide by alkali ion for efficient water oxidation. *J. Am. Chem. Soc.* **141**, 3014-3023 (2019).

7. Zhu, J*. et al.* Pt-Ir-Pd Trimetallic nanocages as a dual catalyst for efficient Ooxygen reduction and evolution reactions in acidic media. *Adv. Energy Mater.* **10**, 1904114 (2020).

8. Lim, J*. et al.* Ultrathin IrO_2_ nanoneedles for electrochemical water oxidation. *Adv. Funct. Mater.* **28**, 1704796 (2018).

9. Park, J., Sa, Y.J., Baik, H., Kwon, T., Joo, S.H. & Lee, K. Iridium-based multimetallic nanoframe@nanoframe structure: An efficient and robust electrocatalyst toward oxygen evolution reaction. *ACS Nano* **11**, 5500-5509 (2017).

10. Yang, L*. et al.* Efficient oxygen evolution electrocatalysis in acid by a perovskite with face-sharing IrO_6_ octahedral dimers. *Nat. Commun.* **9**, 1-9 (2018).

11. Jiang, B*. et al.* Mesoporous metallic iridium nanosheets. *J. Am. Chem. Soc.* **140**, 12434-12441 (2018).

12. Yang, X*. et al.* Highly acid-durable carbon coated Co_3_O_4_ nanoarrays as efficient oxygen evolution electrocatalysts. *Nano Energy* **25**, 42-50 (2016).

13. Weber, D*. et al.* IrOOH nanosheets as acid stable electrocatalysts for the oxygen evolution reaction. *J. Mater. Chem. A* **6**, 21558-21566 (2018).

14. Zhao, Y*. et al.* 3D nanoporous iridium-based alloy microwires for efficient oxygen evolution in acidic media. *Nano Energy* **59**, 146-153, (2019).

15. Lv, F*. et al.* Iridium-tungsten alloy nanodendrites as pH-universal water-splitting electrocatalysts. *ACS Central Sci.* **4**, 1244-1252 (2018).

16. Tackett, B.M*. et al.* Reducing iridium loading in oxygen evolution reaction electrocatalysts using core-shell particles with nitride cores. *ACS Catal.* **8**, 2615-2621 (2018).

17. Pi, Y., Shao, Q., Wang, P., Guo, J. & Huang, X. General formation of monodisperse IrM (M= Ni, Co, Fe) bimetallic nanoclusters as bifunctional electrocatalysts for acidic overall water splitting. *Adv. Funct. Mater.* **27**, 1700886 (2017).

18. Zhu, M., Shao, Q., Qian, Y. & Huang， X. Superior overall water splitting electrocatalysis in acidic conditions enabled by bimetallic Ir-Ag nanotubes. *Nano Energy* **56**, 330-337 (2019).

19. Luo, F*. et al.* Robust and stable acidic overall water splitting on Ir single atoms. *Nano Lett.* **20**, 2120-2128 (2020).

20. Jin, Z*. et al.* Nanoporous Al-Ni-Co-Ir-Mo high-entropy alloy for record-high water splitting activity in acidic environments. *Small* **15**, 1904180 (2019).

21. Wang, C., Sui, Y.M., Xu, M., Liu, C., Xiao, G.J. & Zou, B. Synthesis of Ni-Ir nanocages with improved electrocatalytic performance for the oxygen evolution reaction. *ACS Sustainable Chem. Eng.* **5**, 9787-9792 (2017).

22. Shan, J*. et al.* Charge-redistribution-enhanced nanocrystalline Ru@IrO_x_ electrocatalysts for oxygen evolution in acidic media. *Chem* **5**, 445-459 (2019).

23. Guan, J., Li, D., Si, R., Miao, S., Zhang, F. & Li, C. Synthesis and demonstration of subnanometric iridium oxide as highly efficient and robust water oxidation catalyst. *ACS Catal.* **7**, 5983-5986 (2017).

24. Jiang, H*. et al.* Self-supported iridium oxide nanostructures for electrocatalytic water oxidation in acidic media. *J. Phys. Chem. C* **124**, 2-8 (2019).

25. Zhang, J*. et al.* Iridium nanoparticles anchored on 3D graphite foam as a bifunctional electrocatalyst for excellent overall water splitting in acidic solution. *Nano Energy* **40**, 27-33 (2017).

26. Gao, J., Huang, X., Cai, W., Wang, Q., Jia, C. & Liu, B. Rational design of an iridium-tungsten composite with an iridium-rich surface for acidic water oxidation. *ACS Appl. Mater. Interfaces* **12**, 25991-26001 (2020).
